# Imetelstat-mediated alterations in fatty acid metabolism to induce ferroptosis as a therapeutic strategy for acute myeloid leukemia

**DOI:** 10.1038/s43018-023-00653-5

**Published:** 2023-10-30

**Authors:** Claudia Bruedigam, Amy H. Porter, Axia Song, Gerjanne Vroeg in de Wei, Thomas Stoll, Jasmin Straube, Leanne Cooper, Guidan Cheng, Vivian F. S. Kahl, Alexander P. Sobinoff, Victoria Y. Ling, Billy Michael Chelliah Jebaraj, Yashaswini Janardhanan, Rohit Haldar, Laura J. Bray, Lars Bullinger, Florian H. Heidel, Glen A. Kennedy, Michelle M. Hill, Hilda A. Pickett, Omar Abdel-Wahab, Gunter Hartel, Steven W. Lane

**Affiliations:** 1 Cancer Program, QIMR Berghofer Medical Research Institute, Brisbane, Queensland Australia; 2 School of Biomedical Sciences, The University of Queensland, Brisbane, Queensland Australia; 3 Telomere Length Regulation Unit, Children’s Medical Research Institute, Faculty of Medicine and Health, University of Sydney, Westmead, New South Wales Australia; 4 Division of CLL, Department of Internal Medicine III, Ulm University, Ulm, Germany; 5 Faculty of Engineering, School of Mechanical, Medical and Process Engineering, Queensland University of Technology, Brisbane, Queensland Australia; 6 Department of Hematology, Oncology and Tumor Immunology, Charité University Medicine Berlin, Campus Virchow Klinikum, Berlin, Germany; 7 Hematology, Oncology, Stem Cell Transplantation and Palliative Care, University Medicine Greifswald, Greifswald, Germany; 8 Leibniz Institute on Aging, Jena, Germany; 9 Cancer Care Services, Royal Brisbane and Women’s Hospital, Brisbane, Queensland Australia; 10 Human Oncology & Pathogenesis Program, Memorial Sloan Kettering Cancer Center, New York, NY USA; 11 Statistics Unit, QIMR Berghofer Medical Research Institute, Brisbane, Queensland Australia

**Keywords:** Acute myeloid leukaemia, Drug development, Cancer

## Abstract

Telomerase enables replicative immortality in most cancers including acute myeloid leukemia (AML). Imetelstat is a first-in-class telomerase inhibitor with clinical efficacy in myelofibrosis and myelodysplastic syndromes. Here, we develop an AML patient-derived xenograft resource and perform integrated genomics, transcriptomics and lipidomics analyses combined with functional genetics to identify key mediators of imetelstat efficacy. In a randomized phase II-like preclinical trial in patient-derived xenografts, imetelstat effectively diminishes AML burden and preferentially targets subgroups containing mutant *NRAS* and oxidative stress-associated gene expression signatures. Unbiased, genome-wide CRISPR/Cas9 editing identifies ferroptosis regulators as key mediators of imetelstat efficacy. Imetelstat promotes the formation of polyunsaturated fatty acid-containing phospholipids, causing excessive levels of lipid peroxidation and oxidative stress. Pharmacological inhibition of ferroptosis diminishes imetelstat efficacy. We leverage these mechanistic insights to develop an optimized therapeutic strategy using oxidative stress-inducing chemotherapy to sensitize patient samples to imetelstat causing substantial disease control in AML.

## Abstract (web summary)

Bruedigam et al. explore the mechanism of action and therapeutic benefit of telomerase inhibitor imetelstat in a collection of PDXs from patients with AML and demonstrate that, in combination with chemotherapy, it re-sensitizes treatment-resistant AML cells.

## Main

AML is an aggressive and lethal blood cancer with a 5-year overall survival rate of less than 45% for patients younger than 60 years of age and less than 10% for older patients, predominantly due to disease relapse after chemotherapy or targeted treatments. AML has been extensively classified based on biological features and advances in sequencing technologies have led to a comprehensive genetic classification strategy (European LeukemiaNet, ELN2017)^1,2^. Despite this improved understanding of the individual disease subtypes, targeted treatment algorithms have resulted in only modest clinical benefits to date^3^. The development of effective therapies to improve remission rates and prevent relapse remains a top priority for patients with AML.

Telomerase is an attractive target as it is highly expressed and reactivated in the majority of AML and absent in most cell types including healthy hematopoietic cells. We have previously shown that genetic depletion of telomerase eradicates leukemia stem cells, particularly upon enforced replication^4^. Despite promising preclinical evidence, the development of effective and specific telomerase inhibitors has been challenging. Imetelstat is a first-in-class covalently lipidated 13-mer thiophosphoramidate oligonucleotide that can competitively inhibit telomerase activity by binding to the telomerase RNA component TERC^5^. Imetelstat has shown clinical efficacy in essential thrombocythemia^6^, myelofibrosis^7^ and lower-risk myelodysplastic syndromes^8^. In myelodysplastic syndromes, clinical benefits are associated with reductions in telomerase activity and TERT expression^8^.

In addition to its canonical role as critical regulator of telomere length maintenance, telomerase fulfills important non-canonical roles contributing to stress elimination, regulation of Wnt/β-catenin, NF-κB and p65 signaling, as well as resistance to ionizing radiation^9^. Hence, the clinical activity of imetelstat may be driven by mechanisms independent of telomere shortening and potentially canonical telomerase activity.

Preclinical trials in patient-derived xenografts (PDXs) provide genetically diverse, tractable models to define the efficacy of drugs and to identify biomarkers of response and resistance in AML^10^. PDX-based trials also allow, within the same cohort, the evaluation of new combination therapies with agents that may enhance efficacy and also critically compare their additive value to current, established standard treatments.

In this study, we aimed to assess the preclinical efficacy of imetelstat in a large AML PDX resource that reflects the diversity of genetic abnormalities found in large patient cohorts. We utilized this AML PDX resource to identify biomarkers of resistance and response to imetelstat therapy and to test potentially synergistic combination therapies. To elucidate the mechanism of action of imetelstat in an unbiased manner, we performed genome-wide CRISPR/Cas9 editing allowing the identification of gene knockouts that confer resistance to imetelstat therapy. This study reveals that imetelstat is a potent inducer of ferroptosis that effectively diminishes AML burden and delays relapse following oxidative stress-inducing therapy.

## Results

### Generation of a comprehensive AML PDX resource

**Extended Data Fig. 1:**
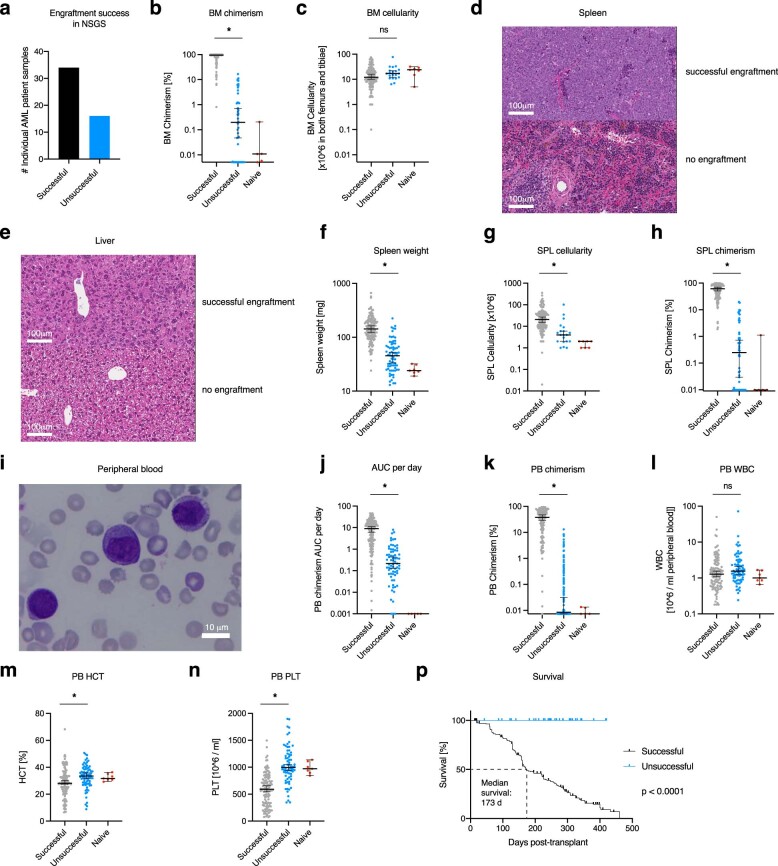
Generation of a comprehensive AML PDX Resource. **a**, The number of AML patient samples that either successfully (gray) or unsuccessfully (blue) generated AML PDX using NSGS^61^ recipients. *N* = 50 patient samples were tested for engraftment in total. **b-n**, AML disease parameters in successfully (*n* = 196) or unsuccessfully (*n* = 73) transplanted NSGS. Bone marrow donor chimerism (**b**) and cellularity (×10^6 harvested from both femurs and tibiae; **c**). Histologic analysis of spleen (**d**) and liver (**e**) morphology in all individual AML PDX models (*n* = 30). Additional images are provided in the supplement. Spleen weight (**f**), spleen cellularity (**g**), and splenic donor chimerism (**h**). Peripheral blood blast morphology analysis at takedown using Wright-Giemsa staining (**i**), donor chimerism area under the curve (AUC) per day (**j**), donor chimerism at takedown (**k**), and white blood cell counts at takedown (**l**). Hematocrit percentage (**m**) and platelets (x10^6/mL; **n**) from peripheral blood at takedown. Data from *n* = 7 naïve NSGS (40 weeks old) are displayed as reference. Data are presented as median ± 95% Confidence Interval [CI]. Statistical analyses were performed on log-transformed data using unpaired two-sided t-test. *P* = 5.79 × 10^−63^ (**b**), *P* = 1.13 × 10^−1^ (**c**), *P* = 9.18 × 10^−31^ (**f**), *P* = 3.59 × 10^−6^ (**g**), *P* = 7.98 × 10^−60^ (**h**), *P* = 1.65 × 10^−27^ (**j**), *P* = 1.34E × 10^−72^ (**k**), *P* = 7.19 × 10^−02^ (**l**), *P* = 2.16 × 10^−03^ (**m**), *P* = 3.18 × 10^−14^ (**n**). Asterisks (*) denote statistically significant comparisons with *P* < 5 × 10^−2^. **p**, AML-related survival analysis of successfully *versus* unsuccessfully generated AML PDX. Median survival was 173 days post-transplant in successfully generated AML PDX *versus* not reached for unsuccessfully generated AML PDX (follow-up > 365 days). Two-tailed *P* < 1 × 10^−4^ according to Gehan–Breslow-Wilcoxon; *n* = 196 (successfully), *n* = 73 (unsuccessfully) transplanted NSGS. Source data

To generate a representative AML PDX inventory, primary bone marrow or blood samples from 50 patients were tested for engraftment and development of AML in NOD/SCID/IL2gR−/−/hIL3,CSF2,KITLG (NSGS). The overall success rate for primary engraftment in NSGS was 70%, defined by bone marrow, spleen or peripheral blood donor chimerism of at least 20%, splenomegaly (spleen weight >70 mg), anemia (HCT < 35%) or thrombocytopenia (PLT < 400 × 10^6 ^ml^−1^), microscopically visible AML infiltration into the spleen or liver and peripheral blood blast morphology (Extended Data Fig. 1a–n). Successfully engrafted NSGS recipients developed AML with a median onset of 173 d post-transplant (Extended Data Fig. 1p).

**Fig. 1:**
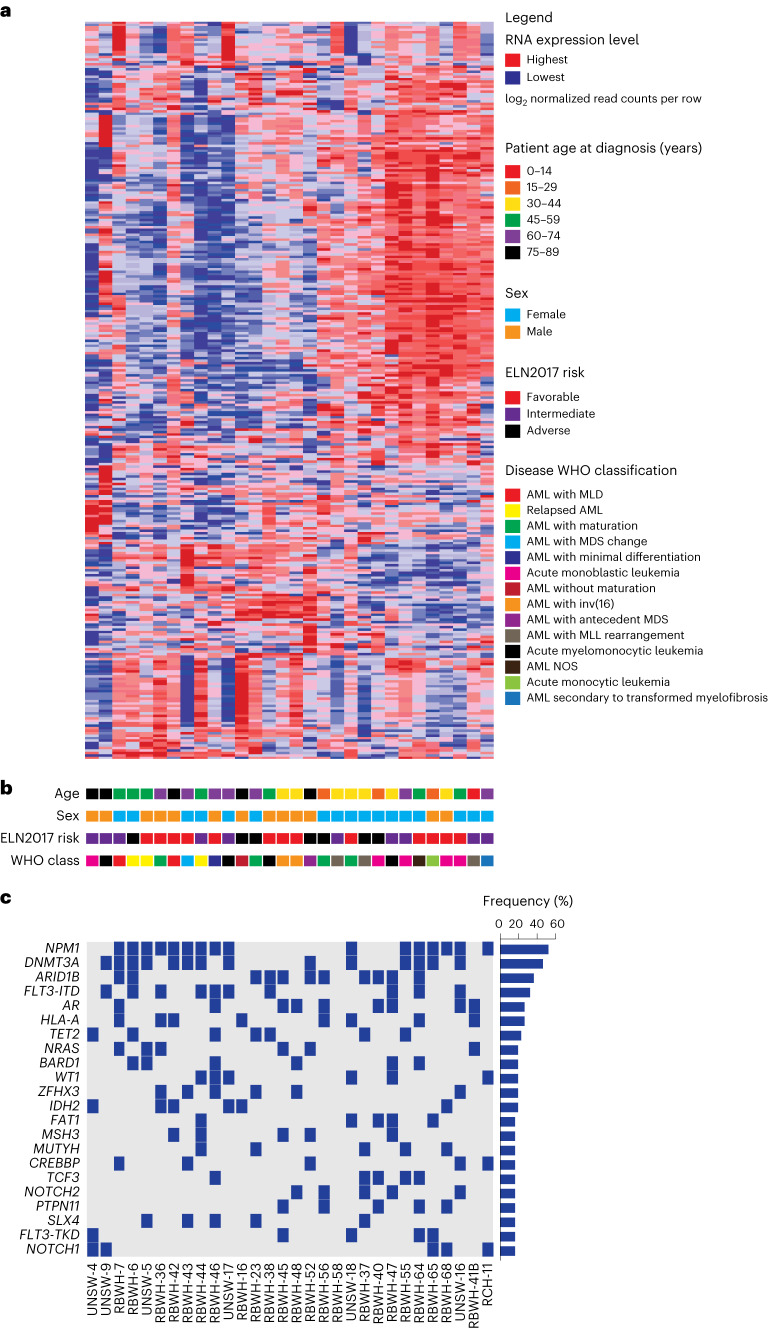
Integrative analysis of samples from patients with AML. **a**, Unsupervised hierarchical clustering analysis on the expression of 300 transcripts with the greatest variance-to-mean ratios among 30 individual AMLs from our repository that can successfully generate AML PDX. MLD, multi-lineage dysplasia; MDS, myelodysplastic syndromes; MLL, mixed-lineage leukemia; NOS, not otherwise specified. **b**, Key clinical characteristics of patients from whom AML samples were derived including age at diagnosis, sex, ELN2017 prognostic risk group and WHO class of disease. **c**, OncoPrint of the most frequently detected mutations in AMLs by targeted next-generation sequencing of 585 genes associated with hematological malignancies (the MSKCC HemePACT assay)^31^. Source data

From the individual samples from patients with AML that successfully engrafted in NSGS, 30 were randomly selected and characterized based on clinical parameters, including patient age, sex, ELN2017 risk, World Health Organization (WHO) disease classification and molecular profiles obtained by transcriptional and mutational sequencing (Fig. 1a–c). All ELN2017 prognostic risk (favorable, intermediate and adverse) and age categories were represented; 17 samples were from female and 13 samples from male AML patient donors (Fig. 1b). Oncogenic mutations were most frequently detected in *NPM1*, *DNMT3A* and *FLT3* loci and overall, this AML PDX resource recapitulated the genetic abnormalities that are observed in large clinical AML cohorts^2^ (Fig. 1c).

### A phase II-like preclinical trial of imetelstat in AML PDX

**Fig. 2:**
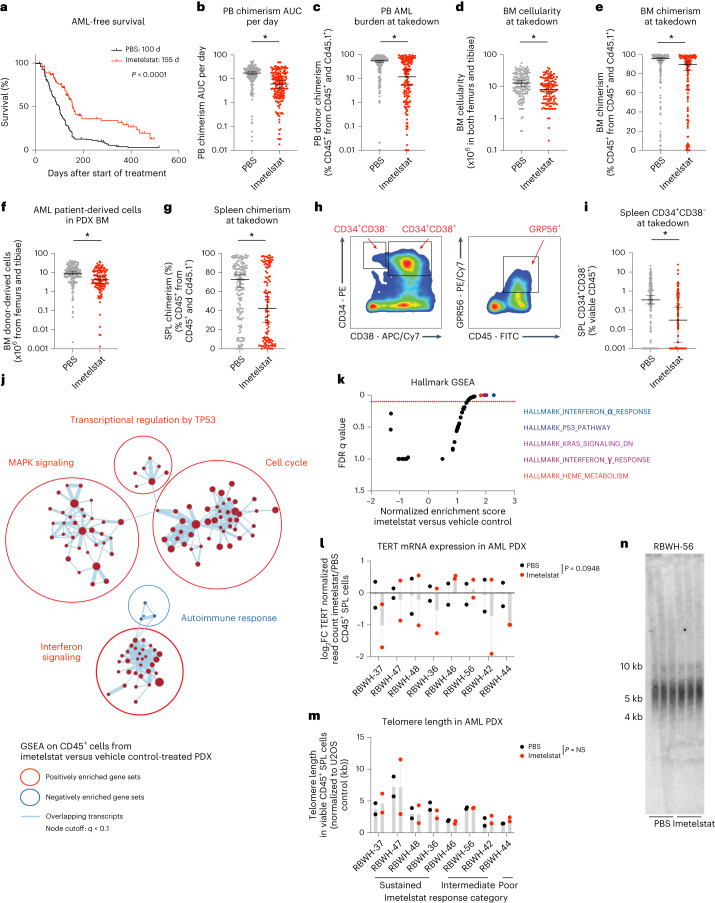
The efficacy of imetelstat in a randomized phase II-like preclinical trial in AML PDX. **a**, Two-tailed Kaplan–Meier survival analysis of vehicle control (PBS; *n* = 180) or imetelstat-treated (*n* = 180) AML PDX. *P* < 1 × 10^−4^ according to Gehan–Breslow–Wilcoxon. **b**–**g**, Analysis of AML disease parameters. Peripheral blood (PB) donor chimerism area under the curve (AUC) per day (**b**), end point PB donor chimerism (**c**), bone-marrow (BM) cellularity (**d**), BM chimerism (**e**), the number of AML donor-derived cells in PDX BM (**f**) and splenic (SPL) donor chimerism (**g**). **h**,**i**, Flow cytometric analysis of AML surface marker expression CD34, CD38 and GPR56. Gating strategy (**h**). The percentage of CD34^+^CD38^−^ viable CD45^+^ SPL singlets (**i**). Data are presented as median ± 95% confidence interval (CI) (**b**–**g**,**i**). Statistical analysis was performed on log-transformed data using an unpaired two-sided *t*-test, considering detection limits at 1 × 10^−3^. *P* = 2.21 × 10^−10^ (**b**), *P* = 7.79 × 10^−8^ (**c**), *P* = 1.37 × 10^−4^ (**d**), *P* = 7.32 × 10^−3^ (**e**), *P* = 8.82 × 10^−5^ (**f**), *P* = 1.83 × 10^−3^ (**g**), *P* = 7.44 × 10^−5^ (**i**). Asterisks (*) denote statistically significant comparisons with *P* < 5 × 10^−2^. **j**,**k**, GSEA on RNA-seq data from sorted viable hCD45^+^ cells collected from imetelstat or PBS-treated AML PDXs. *n* = 16 AML PDXs per treatment group. Cytoscape nodes represent gene sets with a cutoff of *q* < 0.1 (**j**); GSEA on hallmark signatures with the top five enriched signatures highlighted in color (**k**). **l**–**n**, TERT messenger RNA (mRNA) expression results obtained from RNA-seq analysis described as above (**l**). FC, fold change. Telomere length in viable CD45^+^ SPL cells from imetelstat versus PBS-treated AML PDXs measured by qPCR (**m**) and confirmed by telomeric restriction fragment analysis (**n**). Statistical analysis (**l**,**m**) was based on paired two-tailed *t*-tests comparing AML PDXs treated with imetelstat (*n* = 16) or PBS (*n* = 16). *P* = 9.48 × 10^−2^ (**l**), *P* > 5 × 10^−2^ (**m**). Data are presented as mean ± s.e.m. NS, not significant. Source data

**Extended Data Fig. 2:**
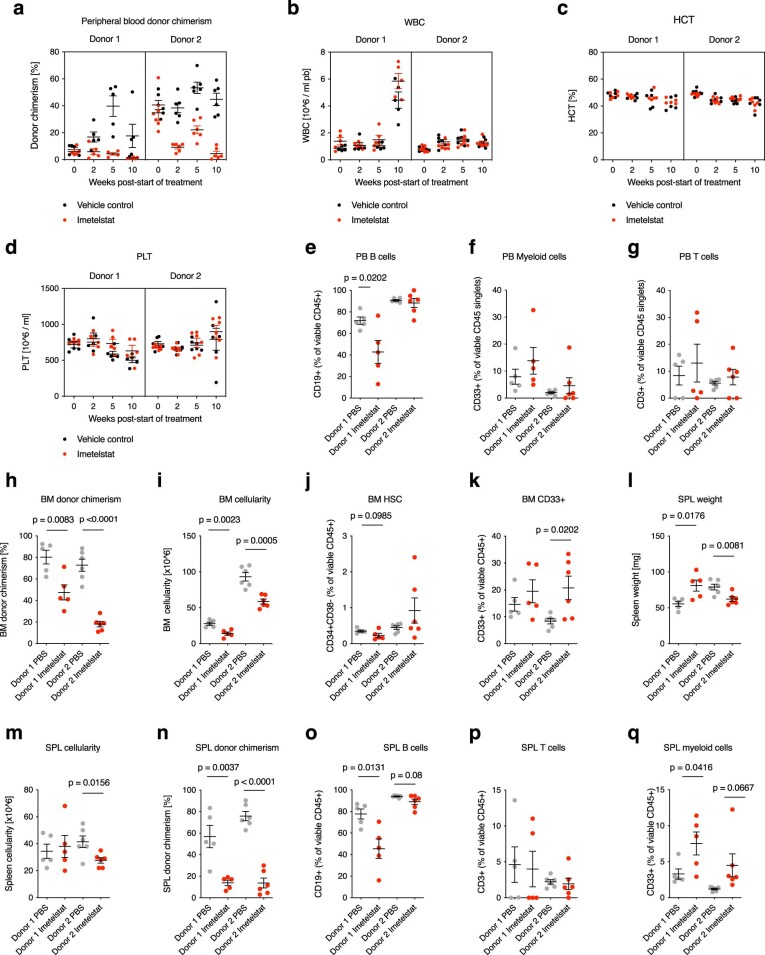
The effect of imetelstat on normal hematopoiesis. Humanized *in vivo* models of hematopoiesis were generated by transplanting viable CD34+ mononuclear cells isolated from cord blood samples from two independent donors into NSG recipients (donor 1: 56,000 cells per NSG, *n* = 5 NSG per treatment group; donor 2: 212,500 cells per NSG, *n* = 6 NSG per treatment group). Recipients were treated for 10 weeks with imetelstat (15 mg/kg body weight) or vehicle control three times per week starting one month after transplantation. **a-d**, Peripheral blood time course analysis of donor chimerism (**a**), white blood cell counts (WBC; **b**), hematocrit (HCT, **c**), and platelet levels (PLT; **d**). **e-g**, Flow cytometry analysis of peripheral blood for B cell surface marker expression (CD19; **e**), myeloid surface marker expression (CD33; **f**), and T cell surface marker expression (CD3; **g**) at 10 weeks post-start of treatment. **h-k**, Bone marrow analysis of cord blood recipients at 10 weeks post-start of treatment: donor chimerism (**h**), cellularity (**i**), hematopoietic stem cell population percentage (CD34+CD38- %; **j**), and myeloid population percentage (CD33+; **k**). **l-q**, Analysis of cord blood recipient’s spleens at 10 weeks post-start of treatment: spleen weight (**l**), cellularity (**m**), donor chimerism (**n**), B cell population (CD19+ %; **o**), T cell population (CD3+ %; **p**), and myeloid cell population (CD33+ %; **q**). **a-q**, Data are presented as mean±SEM. **e-q**, Statistics based on unpaired two-sided t-test comparing imetelstat with PBS-treated groups within each donor: *P* = 2.02 × 10^−2^ (donor 1; **e**), *P* = 8.3 × 10^−3^ (donor 1; **h**), *P* < 1 × 10^−3^ (donor 2; **h**), *P* = 2.3 × 10^−3^ (donor 1; **i**), *P* = 5 × 10^−4^ (donor 2; **i**), *P* = 9 × 10^−2^ (donor 1; **j**), *P* = 2.02 × 10^−2^ (donor 2; **k**), *P* = 1.76 × 10^−2^ (donor 1; **l**), *P* = 8.1 × 10^−3^ (donor 2; **l**), *P* = 1.56 × 10^−2^ (donor 2; **m**), *P* = 3.7 × 10^−3^ (donor 1; **n**), *P* < 1 × 10^−4^ (donor 2; **n**), *P* = 1.31 × 10^−2^ (donor 1; **o**), *P* = 8 × 10^−2^ (donor 2; **o**), *P* = 4.16 × 10^−2^ (donor 1; **q**), *P* = 6.67 × 10^−2^ (donor 2; **q**). Source data

To test the preclinical efficacy of imetelstat in AML, the characterized 30 individual samples from patients with AML were each transplanted into 12 NSGS recipients (*n* = 360 PDXs in total). Once AML burden was detected, PDXs were randomized and treated with imetelstat or vehicle control (PBS) until disease onset or a survival benefit of at least 30 d was reached. Median survival was significantly prolonged in imetelstat compared to PBS-treated PDXs (155 d versus 100 d after start of treatment, *P* < 0.0001; Fig. 2a). AML burden measured as peripheral blood donor chimerism per day was significantly lower in imetelstat compared to vehicle-treated recipients (Fig. 2b). Moreover, end point peripheral blood donor chimerism, bone-marrow cellularity and donor chimerism as well as the absolute number of AML patient-derived cells were significantly reduced in recipients treated with imetelstat when compared to vehicle control (Fig. 2c–f). Furthermore, imetelstat treatment significantly reduced splenic AML donor chimerism (Fig. 2g). We next assessed AML surface marker expression associated with leukemia-initiating activity^11–13^ (Fig. 2h). Imetelstat significantly diminished the CD34^+^CD38^−^ leukemic stem cell-enriched splenic AML cell population (Fig. 2i). In normal human hematopoiesis using two independent CD34-enriched cord blood xenografts in NSG recipients, the effects of imetelstat were predominantly seen in B lymphocytes with relative preservation of the myeloid and stem cell populations (Extended Data Fig. 2a–q).

**Extended Data Fig. 3:**
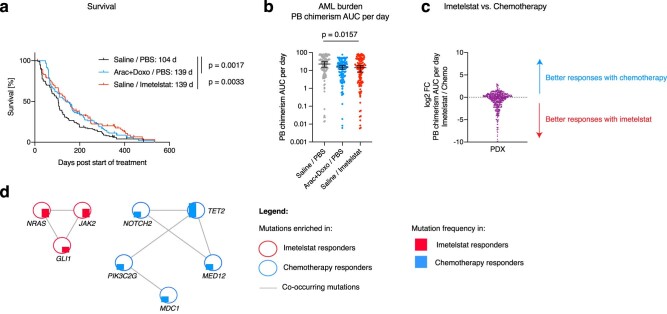
Comparative analysis of AML PDX responses to imetelstat versus standard induction chemotherapy. **a**, Median survival was 104 (vehicle control (Saline / PBS) – treated PDX; black; *n* = 120 PDX) versus 139 (chemotherapy-treated PDX; Arac+Doxo; blue; *P* = 1.7 × 10^−3^; *n* = 120 PDX) and 139 (imetelstat-treated PDX; Saline / Imetelstat; red; *P* = 3.3 × 10^−3^; *n* = 120 PDX) according to Gehan–Breslow-Wilcoxon (two-sided Kaplan–Meier analysis). **b**, AML burden quantified as peripheral blood donor chimerism per day. Statistics based on ordinary One-way-ANOVA adjusted for multiple comparisons. *N* = 120 PDX per treatment group. *P* = 1.57 × 10^−2^ (vehicle versus imetelstat). **c**, Imetelstat versus chemotherapy response calculation as log2FC of peripheral blood chimerism area under the curve per day in AML PDX. The red arrow indicates samples defined as preferential imetelstat responders, and conversely, the blue arrow indicates PDX defined as preferential chemotherapy responders. **d**, Cytoscape visualization of genes with mutations identified exclusively in preferential imetelstat responders (red) or chemotherapy responders (blue) at baseline. Source data

We next aimed to compare imetelstat responses to those obtained with standard induction chemotherapy (cytarabine plus anthracycline) in AML PDX from 20 individual samples from patients with AML in an independent cohort using NOD.Rag1−/−Il2Rg−/−/ hIL3,CSF2,KITLG (NRGS) recipients^14^. Imetelstat matched the similar benefit conveyed by standard chemotherapy (139 d) comparative to 104 d in the vehicle control group and this was accompanied by significant reductions in peripheral blood AML burden (Extended Data Fig. 3a,b); however, the individual samples from patients with AML could be classified into either preferential imetelstat or preferential chemotherapy responders (Extended Data Fig. 3c). Preferential responses to imetelstat when compared to standard induction chemotherapy were associated with baseline mutations in *NRAS*, *JAK2* or *GLI1* (Extended Data Fig. 3d).

We next assessed the transcriptional consequences of imetelstat therapy in a cohort of PDX from eight randomly chosen individual samples from patients with AML in vivo (*n* = 4 sustained (RBWH-37, −47, −48 and −36), *n* = 3 intermediate (RBWH-46, −56 and −42) and *n* = 1 poor (RBWH-44) responders to imetelstat). Gene expression signatures annotated as interferon signaling, cell cycle, transcriptional regulation by TP53 and MAPK signaling were significantly enriched in AML donor cells from imetelstat-treated compared to vehicle control-treated PDX (Fig. 2j,k). TERT messenger RNA expression levels were trend-wise reduced in AML donor cells derived from imetelstat-treated compared to vehicle-treated PDX spleens (Fig. 2l). Notably, telomere lengths were similar between imetelstat-treated compared to vehicle-treated groups (Fig. 2m,n).

### A CRISPR/Cas9 screen to identify key effectors of imetelstat

**Fig. 3:**
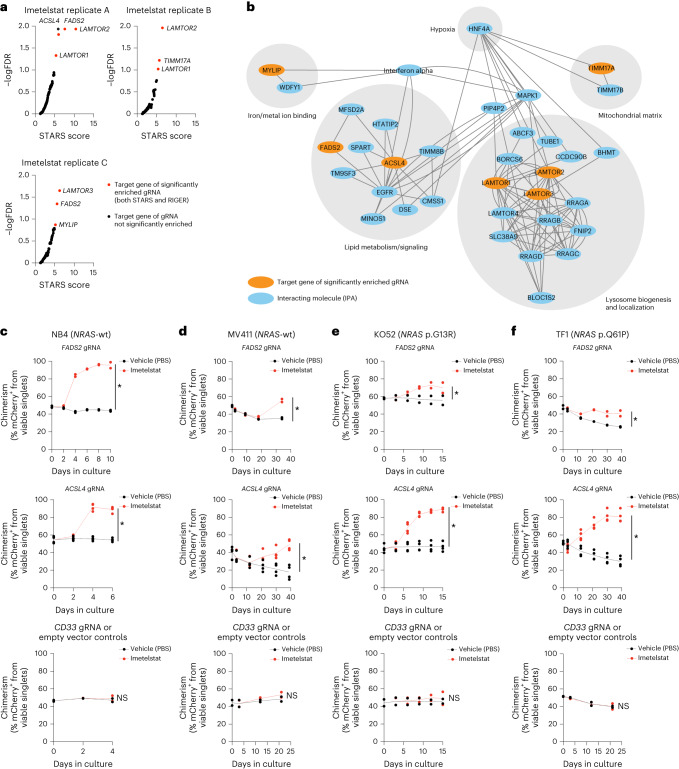
Identification of key mediators of imetelstat efficacy using genome-wide CRISPR/Cas9 editing. Brunello CRISPR/Cas9 positive enrichment screen in NB4 cells. **a**, gRNA enrichment analysis using STARS and RIGER gene-ranking algorithms in *n* = 3 independent imetelstat-treated biological replicates. Red circles indicate significantly enriched targets (STARS false discovery rate (FDR) < 0.15 and RIGER score >2.0). **b**, Cytoscape visualization of the ingenuity pathway analysis (IPA)-derived interaction network connecting the identified significantly enriched gRNA targets. **c**–**f**, Competition assays of imetelstat- (red) versus vehicle control (PBS; black)-treated Cas9-expressing NB4 (**c**), MV411 (**d**), KO52 (**e**) and TF1 (**f**) cultures transduced with *n* = 2 independent sgRNAs targeting *FADS2* (top), *n* = 4 independent sgRNAs targeting *ACSL4* (middle) and *n* = 2 controls (empty vector and gRNA targeting *CD33*). Three technical replicates per condition from two independent experiments were pooled. Asterisks (*) denote statistically significant comparisons based on distinct 95% CI on mCherry chimerism AUC between imetelstat and PBS-treated cultures. 95% CI (lower limit, upper limit): NB4 *FADS2* PBS (444.4, 456.7) versus imetelstat (771.9, 795.9); *ACSL4* PBS (320.1, 344.4) versus imetelstat (428.3, 459.6); editing controls PBS (186.0, 197.8) versus imetelstat (185.4, 203.4) (**c**). MV411 *FADS2* PBS (1,253, 1,302) versus imetelstat (1,421, 1,525); *ACSL4* PBS (847.8, 1,160) versus imetelstat (1,262, 1,564); editing controls PBS (896.2, 1,030) versus imetelstat (943.8, 1,073) (**d**). KO52 *FADS2* PBS (812.3, 907.0) versus imetelstat (949.8, 1,023); *ACSL4* PBS (648.9, 743.3) versus imetelstat (1,020, 1,095); editing controls PBS (635.3, 727.6) versus imetelstat (654.9, 760) (**e**). TF1 *FADS2* PBS (1,280, 1,325) versus imetelstat (1,585, 1,721); *ACSL4* PBS (1,381, 1,644) versus imetelstat (2,486, 2,788); editing controls PBS (905.9, 982.8) versus imetelstat (888.2, 993.3) (**f**). Source data

**Extended Data Fig. 4:**
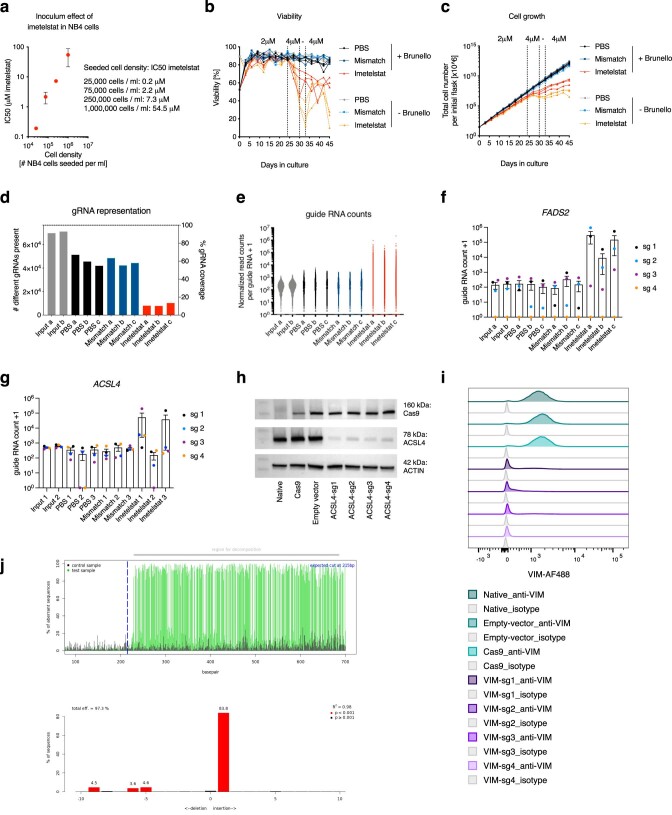
Identification of key mediators of imetelstat efficacy using a genome-wide CRISPR/Cas9 screen. **a**, Identification of an imetelstat inoculum effect by quantification of IC50 values in NB4 cultures seeded at different densities: IC50 = 0.2 microM (25,000 cells per ml); IC50 = 1.5 microM (75,000 cells per ml); IC50 = 7.2 microM (250,000 cells / ml); IC50 = 31.3 microM (1,000,000 cells / ml). Dots represent mean IC50 values ± SEM from *n* = 2 independent experiments. **b**,**c**, Viability (**b**) and cell growth analysis (**c**) of Brunello-library-transduced NB4 cells cultured in the presence of vehicle control (PBS), mismatch control (MM1), or imetelstat compared to non-transduced NB4 cells over a time course of 45 days. The respective concentrations are indicated in the panel. *N* = 3 independent biological replicates per genotype and treatment condition. **d**,**e**, Next generation sequencing of DNA isolated from Brunello library-transduced NB4 cells harvested at day 45 in culture treated with vehicle control (PBS; black bars), mismatch control (MM1; blue bars), or imetelstat (red bars), and compared to DNA isolated from Brunello library-transduced NB4 cells before treatment as input control (gray bars): **d**, The number of different guide RNAs present (left y-axis) and percentage of guide RNA coverage (right y-axis); **e**, the read counts obtained from each guide RNA. **f**,**g**, Read counts obtained from *n* = 4 independent *FADS2* (**f**) or *n* = 4 independent *ACSL4* (**g**) targeting guide RNAs in the input, vehicle (PBS), mismatch (MM1) or imetelstat-treated Brunello-transduced NB4 cultures. Data are presented as mean ± SEM. **h-j**, Confirmation of efficient CRISPR/Cas9 - generated knockdowns in human AML cell lines by ACSL4 western blotting (**h;** additional biological replicates are provided as supplement), flow cytometric analysis of intracellular VIM expression (**i**), and *FADS2* gene editing by tracking of indels by decomposition analysis using TIDE analysis tool (http://shinyapps.datacurators.nl/tide/) (**j**). Source data

To investigate the mechanism of action of imetelstat in AML in an unbiased manner, we applied the Brunello guide RNA (gRNA) library^15^ as a positive selection screen to identify gene knockouts that confer resistance to imetelstat. We used NB4 cells as these demonstrated highest sensitivity to imetelstat when compared to 13 other human hematopoietic cell lines (Extended Data Fig. 7a). Half-maximum inhibitory concentration (IC_50_) values strongly depended on cell density, demonstrating the presence of an imetelstat inoculum effect (Extended Data Fig. 4a)^16^. Cas9-expressing NB4 cells transduced with the Brunello library or untransduced controls were cultured in the presence of imetelstat concentrations that resulted in substantial cell death (IC98) of the untransduced control cultures but allowed the enrichment of imetelstat-resistant cells in Brunello-transduced cultures over a time course of 45 d in culture (Extended Data Fig. 4b). Vehicle or mismatch control-treated NB4 cells grew exponentially throughout the course of treatment (Extended Data Fig. 4c). Specific guide RNAs were selectively enriched in Brunello-transduced imetelstat-resistant compared to vehicle-treated and input control cultures (Extended Data Fig. 4d–g). Combined RIGER and STARS gene-ranking algorithms identified seven significant hits: fatty acid desaturase 2 (FADS2), acyl-CoA synthetase long-chain family member 4 (ACSL4), translocase of inner mitochondrial membrane 17A (TIMM17A), late endosomal/lysosomal adaptor, MAPK and MTOR activator 1–3 (LAMTOR1, LAMTOR2, LAMTOR3) and myosin regulatory light-chain interacting protein (MYLIP; Fig. 3a). Ingenuity pathway analysis indicated close functional relationships between the seven hits in regulating lipid metabolism, iron/metal ion binding, mitochondrial matrix and lysosome biogenesis and localization (Fig. 3b).

We next aimed to validate the most significant hits identified (FADS2 and ACSL4) using single guide RNA (sgRNA)-mediated editing in the *NRAS* wild-type expressing NB4 and MV411 and the *NRAS*-mutant KO52 (p.G13R) and TF1 (p.Q61P) AML cell lines. Editing was confirmed by TIDE analysis^17^ and reduced protein levels (Extended Data Fig. 4h–j).

We performed competition assays to confirm that loss-of-function editing of *FADS2* or *ACSL4* confers competitive growth advantage under imetelstat pressure in all AML cell lines analyzed (Fig. 3c–f). The observed effects were target-specific as a competitive outgrowth under imetelstat pressure was not observed when CD33 (predicted to have neutral effects on cell functions^18^) knockouts or empty vector controls were used (Fig. 3c–f).

These results demonstrate that loss-of-function editing of *FADS2* or *ACSL4* confers competitive growth advantage under imetelstat pressure, identifying ACSL4 and FADS2 as mediators of imetelstat efficacy in AML.

### Imetelstat is a potent inducer of ferroptosis

**Fig. 4:**
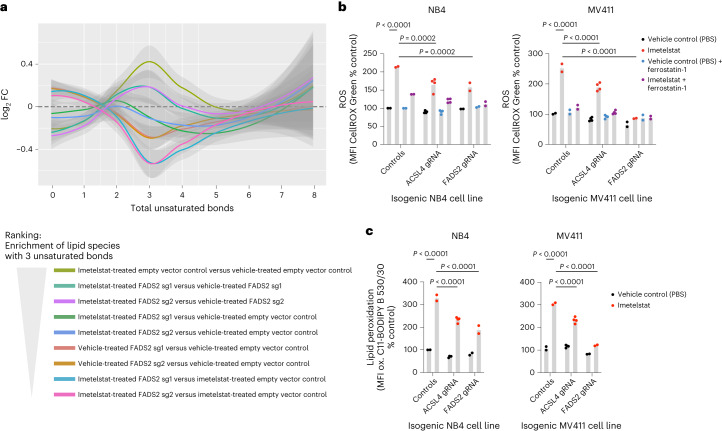
Imetelstat is a potent inducer of ferroptosis. **a**, Lipid desaturation analysis of *FADS2*-edited (FADS2-sg1 and FADS2-sg2) or non-edited (empty vector control) NB4 cells treated with imetelstat (4 μM at a seeding density of 2.5 × 10^5^ cells per ml culture) or vehicle control (PBS) for 24 h. The graph depicts the median log_2_FC of the number of total unsaturated bonds in lipid species in the respective comparisons outlined in the legend. Shading represents the 95% CI. *n* = 3 replicates from distinct cell passages and independent experiments. **b**,**c**, CellROX Green (**b**) and C11-BODIPY (**c**) analysis in *ACSL4*-edited (*n* = 4 independent gRNAs), *FADS2*-edited (*n* = 2 independent gRNAs) or non-edited (*n* = 2 independent replicates, Cas9, empty vector) NB4 or MV411 cell lines treated with imetelstat (4 μM) or PBS. Time points of analysis were 24 h (NB4) and day 4 (MV411). Three technical replicates per condition were pooled. Data are presented as mean ± s.e.m. One-way analysis of variance (ANOVA) was used and adjusted for multiple comparisons. NB4, *P* < 1 × 10^−4^ (non-edited + PBS versus non-edited + imetelstat), *P* = 2 × 10^−4^ (non-edited + imetelstat versus *ACSL4-*edite*d* + imetelstat), *P* = 2 × 10^−4^ (non-edited + imetelstat versus *FAD2S-*edited + imetelstat); MV411, *P* < 1 × 10^−4^ (non-edited + PBS versus non-edited + imetelstat), *P* < 1 × 10^−4^ (non-edited + imetelstat versus *ACSL4*-edited + imetelstat), *P* < 1 × 10^−*4*^ (non-edited + imetelstat versus *FADS2-*edited + imetelstat) (**b**). NB4, *P* < 1 × 10^−4^ (non*-*edited + PBS versus non-edited + imetelstat), *P* < 1 × 10^−4^ (non-edited + imetelstat versus *ACSL4-*edited + imetelstat), *P* < 1 × 10^−4^ (non-edited + imetelstat versus *FADS2*-edited + imetelstat); MV411, *P* < 1 × 10^−4^ (non-edited + PBS versus non-edited + imetelstat), *P* < 1 × 10^−4^ (non-edited + imetelstat versus *ACSL4-*edited + imetelstat), *P* < 1 × 10^−4^ (non-edited + imetelstat versus *FADS2-*edited + imetelstat) (**c**). A repeat experiment was performed that replicated the results. Source data

**Extended Data Fig. 5:**
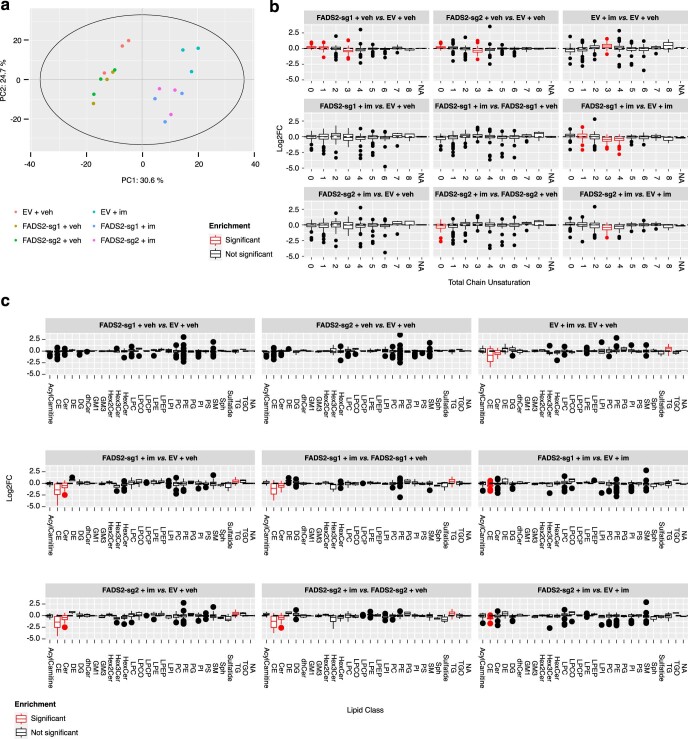
Lipidomics analysis of imetelstat-treated *FADS2*-edited or non-edited NB4 cells. Targeted lipidomics analysis on 593 lipid species and their desaturation levels. **a**, PCA plot on normalized peak areas of lipid species in *FADS2*-edited (FADS2-sg1, FADS2-sg2) or non-edited (empty-vector control) NB4 cultures supplemented for 24h with either 4 microM imetelstat or vehicle control. **b**,**c**, Differential enrichment analysis using lipidR tool of unsaturated bonds in all measured lipid species (**b**), and lipid species according to lipid classes (**c**). Red box plots denote significantly different comparisons with *P* < 5 × 10^−3^. *N* = 3 independent replicates per condition from distinct cell passages. Lipid classes: Acylcarnitines (AcylCarnitine), Cholesteryl ester (CE), Ceramide (Cer), Desmosterol (DE), Diacylglycerol (DG), Dihydroceramide (dhCer), GM1 ganglioside (GM1), GM3 ganglioside (GM3), Dihexosylceramide (Hex2Cer), Trihexosylcermide (Hex3Cer), Monohexosylceramide (HexCer), Lysophosphatidylcholine (LPC), Lysoalkylphosphatidylcholine (LPCO), Lysoalkenylphosphatidylcholine (LPCP), Lysophosphatidylethanolamine (LPE), Lysoalkenylphosphatidylethanolamine (LPEP), Lysophosphatidylinositol (LPI), Phosphatidylcholine (PC), Phosphatidylethanolamine (PE), Phosphatidylglycerol (PG), Phosphatidylinositol (PI), Phosphatidylserine (PS), Sphingomyelin (SM), Sphingosine (Sph), Sulfatide (Sulfatide), Triacylglycerol (TG), Alkyldiacylglycerol (TGO). NA: Molecules which could not be parsed by lipidr (for example ubiquinone, free cholesterol). Source data

*ACSL4* and *FADS2* encode key enzymes regulating polyunsaturated fatty acid (PUFA)-containing phospholipid synthesis. FADS2 is a key enzyme in a lipid metabolic pathway that converts the essential fatty acids linoleate (18:2n6) and α-linolenate (C18:3n3) into long-chain PUFAs^19^. Targeted lipidomics analysis on 593 lipid species and their desaturation levels^20^ demonstrated clear effects of imetelstat treatment and *FADS2* editing on the cellular lipidome, with imetelstat-treated empty vector control AML cells showing greatest difference to vehicle-treated empty vector control and *FADS2*-edited cells. (Extended Data Fig. 5a). Moreover, we found a significant enrichment of phospholipids containing fatty acids with three unsaturated bonds in imetelstat-treated compared to vehicle control-treated NB4 cells and this enrichment of lipid desaturation was diminished by *FADS2* editing (Fig. 4a and Extended Data Fig. 5b). Moreover, imetelstat increased the levels of phospholipids with triglycerides and reduced the levels of phospholipids containing cholesteryl esters and ceramides when compared to vehicle control in an FADS2-dependent manner (Extended Data Fig. 5c). Taken together, these data demonstrate imetelstat-induced PUFA phospholipid synthesis in an FADS2-dependent manner.

ACSL4 has been previously identified as key regulator of ferroptosis^21^. Ferroptosis is a form of cell death that is driven by an imbalance between the production of reactive oxygen species (ROS) during lipid peroxidation and the antioxidant system and may involve autophagic processes depending on the trigger^22^. A hallmark of ferroptosis is lipid peroxidation, the oxidation of PUFA-containing phospholipids that occurs via a free radical chain reaction mechanism^22^. Cancer therapies can enhance ferroptosis sensitivity via lipid remodeling that increases levels of peroxidation-susceptible PUFA-containing phospholipids^23^.

**Fig. 5:**
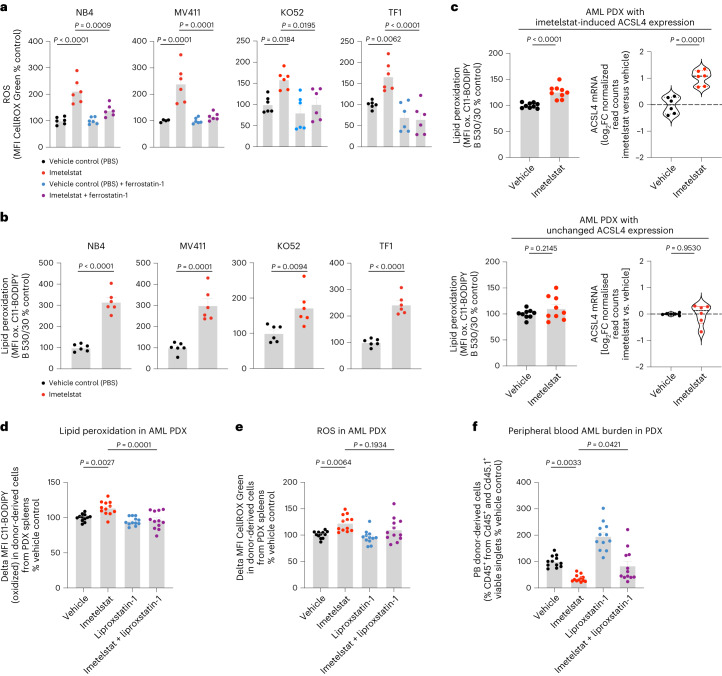
Lipid ROS scavenging diminishes imetelstat efficacy. **a**,**b**, CellROX Green (**a**) and C11-BODIPY (**b**) flow cytometry on NB4, MV411, KO52 and TF1 treated with imetelstat (4 μM) or vehicle control (PBS). *n* = 6 replicates pooled from two experiments. Time points of analysis were 24 h (NB4) and day 4 (MV411), day 8 (KO52) and day 5 (TF1). Data are presented as mean ± s.e.m. **a**, One-way ANOVA was used and adjusted for multiple comparisons. NB4, *P* < 1 × 10^−4^ (NB4 PBS versus imetelstat), *P* = 9 × 10^−4^ (imetelstat versus imetelstat + ferrostatin); MV411, *P* = 1 × 10^−4^ (*P*BS versus imetelstat), *P* = 1 × 10^−4^ (imetelstat versus imetelstat + ferrostatin); KO52, *P* = 1.84 × 10^**−**2^ (PBS versus imetelstat), *P* = 1.95 × 10^−2^ (imetelstat versus imetelstat + ferrostatin); TF1, *P* = 6.2 × 10^−3^ (PBS versus imetelstat), *P* < 1 × 10^−4^ (imetelstat versus imetelstat + ferrostatin). **b**, An unpaired two-sided *t*-test was used. NB4, *P* < 1 × 10^−4^; MV411, *P* = 1 × 10^−4^; KO52, *P* = 9.4 × 10^−3^; TF1, *P* < 1 × 10^−4^. **c**, C11-BODIPY and ACSL4 messenger RNA (mRNA) analysis on sorted viable CD45^+^ splenic cells from imetelstat- compared to PBS-treated PDXs from the preclinical trial presented in Fig. 2. C11-BODIPY data (*n* = 9 PDXs from three individual AML samples with three PDXs per patient sample) are presented as mean ± s.e.m. ACSL4 mRNA data (*n* = 6 PDXs from the same three individual AML samples with two PDXs per patient sample) are presented as violin plots. Statistics are based on an unpaired two-sided *t*-test: *P* < 1 × 10^−4^ (MFI C11-BODIPY, top), *P* = 2.145 × 10^−1^ (MFI C11-BODIPY, bottom); *P* = 1 × 10^−4^ (ACSL4, top), *P* = 9.53 × 10^−1^ (ACSL4, bottom). **d**–**f**, AML PDX treated with vehicle, liproxstatin-1, imetelstat or a combination of liproxstatin-1 with imetelstat for 2 weeks. *n* = 12 PDX per treatment group. C11-BODIPY (**d**) and CellROX (**e**) flow cytometry on splenic CD45^+^ singlets. PB chimerism (**f**) at the end of treatment. Data are presented as mean ± s.e.m. (**d**–**f**). One-way ANOVA was used and adjusted for multiple comparisons. *P* = 2.7 × 10^−3^ (vehicle versus imetelstat), *P* = 1 × 10^−3^ (imetelstat versus imetelstat + liproxstatin-1) (**d**). *P* = 6.4 × 10^−3^ (vehicle versus imetelstat), *P* = 1.934 × 10^−1^ (imetelstat versus imetelstat + liproxstatin-1) (**e**). *P* = 3.3 × 10^−3^ (vehicle versus imetelstat), *P* = 4.21 × 10^−2^ (imetelstat versus imetelstat + liproxstatin-1) (**f**). Source data

**Extended Data Fig. 6:**
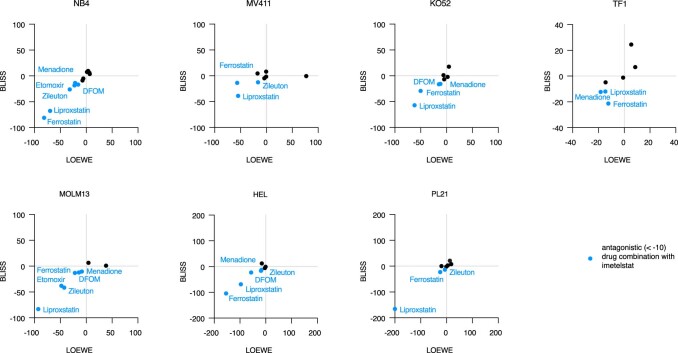
The effect of chemical perturbation of ferroptosis on imetelstat efficacy. Celltiter-based cell growth analysis in a panel of *n* = 7 imetelstat-sensitive cell lines NB4, MV411, KO52, TF1, MOLM13, HEL or PL21 that were supplemented with different concentrations of imetelstat combined with various pharmacological modulators of ferroptosis (that is ferrostatin-1, liproxstatin-1, DFOM, zileuton, menadione, etomoxir, RSL3, erastin). Synergism was estimated using the Synergyfinder 2.0 algorithm (http://synergyfinder.fimm.fi) on the viability data pooled from *n* = 2 independent experiments. BLISS and LOEWE synergy scores are plotted for each drug combination and cell line. Ferroptosis modulators highlighted in blue represent predicted antagonistic combinations with imetelstat with LOEWE *AND* BLISS scores < 10. Source data

To test whether imetelstat induces lipid peroxidation, we treated various AML cell lines with C11-BODIPY, a fluorescent fatty acid probe that changes its emission spectrum from red to green upon oxidation. In all four AML cell lines tested, imetelstat treatment resulted in a significant increase in mean fluorescence intensity (MFI) of the oxidized fatty acid probe, demonstrating that imetelstat induces lipid peroxidation in AML cells in vitro (Fig. 5b). We next assessed whether also ROS levels were affected by imetelstat. Using CellROX Green to measure ROS production, we found that its MFI was increased by imetelstat and this increase was diminished when the lipid ROS scavenger ferrostatin-1 was added during the incubation step with CellROX Green, demonstrating that imetelstat increases predominantly lipid ROS levels in AML cell lines in vitro (Fig. 5a). Both lipid peroxidation and lipid ROS production were significantly diminished in *ACSL4* or *FADS2* loss-of function edited AML cell lines, demonstrating that imetelstat-induced lipid peroxidation and lipid ROS production are dependent on functional FADS2 and ACSL4 in vitro (Fig. 4b,c). Pharmacological inhibition of ferroptosis using the lipid ROS scavengers ferrostatin-1 and liproxstatin-1 diminished imetelstat efficacy in all AML cell lines tested (Extended Data Fig. 6). Moreover, the iron chelator deferoxamine mesylate, the 5-lipoxygenase inhibitor zileuton and menadione diminished imetelstat-induced cell death in a substantial proportion of AML cell lines tested (Extended Data Fig. 6).

In AML PDXs in vivo, imetelstat-induced lipid peroxidation was associated with increased *ACSL4* expression (Fig. 5c). To investigate whether lipid ROS and lipid peroxidation are essential for imetelstat’s mechanism of action in AML PDXs in vivo, we treated AML PDXs with either vehicle control, imetelstat (15 mg kg^−1^ three times per week), liproxstatin-1 (15 mg kg^−1^ twice daily) or the combination of both imetelstat and liproxstatin-1 for 2 weeks. Imetelstat-driven lipid peroxidation and ROS production were prevented by liproxstatin treatment (Fig. 5d,e). In vivo liproxstatin treatment diminished imetelstat efficacy in PDXs as measured by peripheral blood AML burden (Fig. 5f).

Taken together, these data provide evidence that imetelstat is a potent inducer of ferroptosis through ACSL4- and FADS2-mediated alterations in PUFA metabolism, excessive lipid peroxidation and oxidative stress.

### Lipophagy precedes imetelstat-induced ferroptosis

**Fig. 6:**
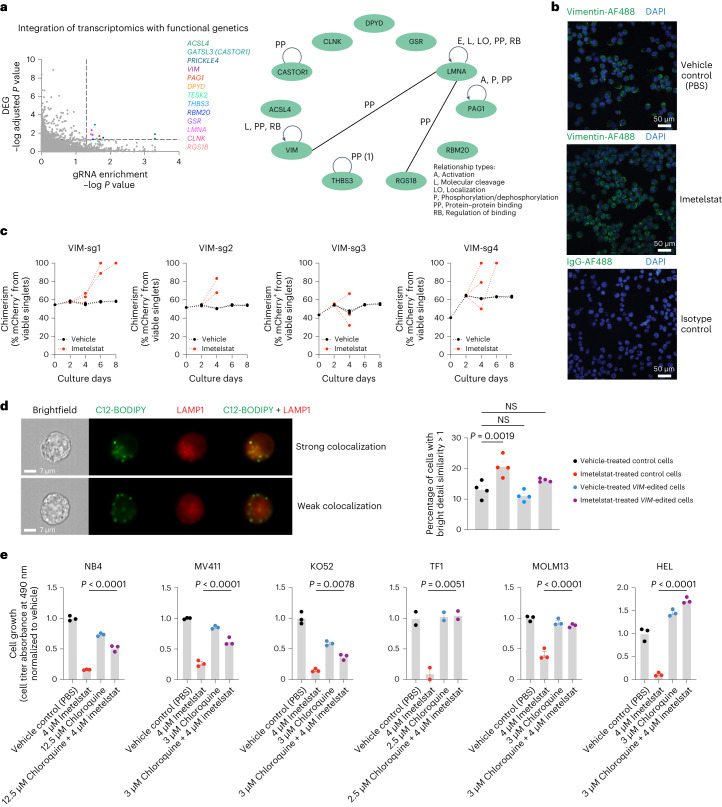
Integrative analysis of transcriptomics and functional genetics. **a**, Integration of RNA-seq and CRISPR screen data using relaxed cutoffs (differential gene expression analysis-derived adjusted *P* < 0.05 and gRNA enrichment analysis-derived RIGER *P* < 0.05). Thirteen genes (colored dots) passed these cutoff criteria, of which 11 were annotated in ingenuity pathway analysis (IPA) (right). A common regulatory module for VIM, LMNA and RGS18 is highlighted through connecting lines. DEG, differentially expressed gene. **b**, Confocal microscopy of VIM protein in NB4 cells treated with vehicle control (PBS) or imetelstat for 24 h. Representative images of *n* = 6 biological replicates. DAPI, 4,6-diamidino-2-phenylindole. **c**, *VIM*-editing in NB4 using *n* = 4 independent sgRNAs. Competition assays of mCherry^+^ *VIM*-edited cells grown in the presence of mCherry-unedited control NB4 cells, treated with imetelstat (red) or vehicle (PBS) control (black). Plots show data from one representative experiment. Two independent repeats were performed. **d**, Imaging flow cytometry of lipophagy using C12-BODIPY and LAMP1 in *n* = 4 independent *VIM*-edited (VIM-sg1, VIM-sg2, VIM-sg3 and VIM-sg4) or *n* = 4 independent editing-control (native, Cas9, empty vector or CD33-sg2) NB4 cell lines. Recovery examples of cells showing strong colocalization of C12-BODIPY and LAMP1 indicative of lipophagy activity (top) or cells with weak colocalization indicating insignificant lipophagic flux. Quantification of the percentages of cells with strong colocalization defined as bright detail similarity score >1. Data are presented as mean ± s.e.m. Statistics are based on a one-way ANOVA adjusted for multiple comparisons to PBS-treated editing controls. Editing controls + PBS versus editing controls + imetelstat, *P* = 1.9 × 10^−3^; editing controls + PBS versus *VIM*-edited + PBS, *P* = 4.955 × 10^−1^; editing controls + PBS versus VIM-edited + imetelstat, *P* = 2.128 × 10^−1^. Comparisons were considered NS when *P* > 5 × 10^−2^. Data are from one experiment representative of four independent experiments. This experiment was repeated three times with similar results. **e**, Chloroquine and imetelstat combination treatments in AML cell lines. Data are presented as mean ± s.e.m. One-way ANOVA was used and adjusted for multiple comparisons. NB4 (*n* = 3 replicates), *P* < 1 × 10^−4^; MV411 (*n* = 3 replicates), *P* < 1 × 10^−4^; KO52 (*n* = 3 replicates), *P* = 8 × 10^−3^; TF1 (*n* = 2 replicates), *P* = 5.1 × 10^−3^; MOLM13 (*n* = 3 replicates), *P* < 1 × 10^−4^; HEL (*n* = 3 replicates), *P* < 1 × 10^−4^. Each experiment was repeated once with similar results. Source data

By integrating transcriptomics and functional genetics, we aimed to investigate the mechanism by which imetelstat induces ferroptosis. We performed an overlay of the in vivo AML PDX RNA-seq datasets from imetelstat and vehicle-treated mice with the Brunello library CRISPR/Cas9 knockout screen data (cutoff criteria of RNA-seq adjusted *P* value < 0.05 and RIGER *P* < 0.05) and identified 11 imetelstat target candidates (Fig. 6a). Two of them, VIM (vimentin) and LMNA (lamin A/C), which are part of a common regulatory module (Fig. 6a), have recently been identified as telomeric G-quadruplex-binding proteins^24^.

**Extended Data Fig. 7:**
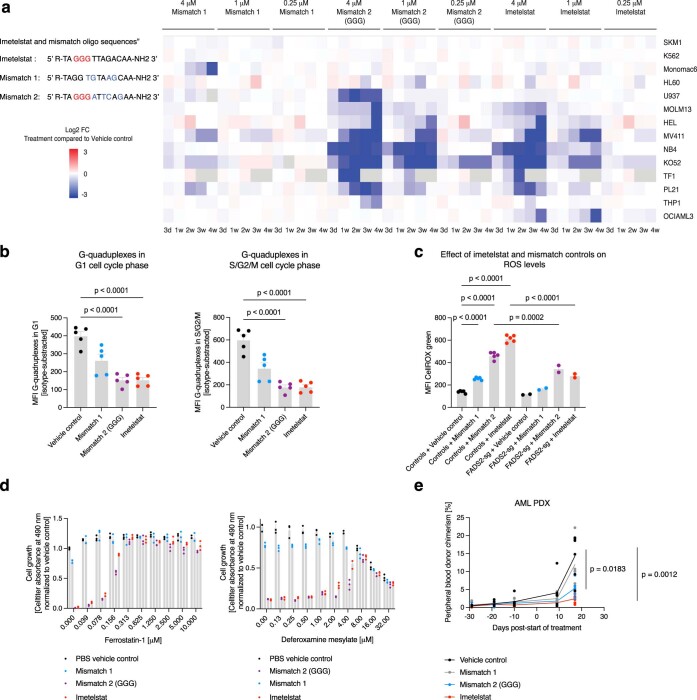
The effect of imetelstat’s triple G-containing mismatch control on AML. **a**, Oligonucleotide sequences of imetelstat, mismatch 1 (MM1), and mismatch 2 (GGG; MM2). Celltiter analysis of 14 human hematopoietic cell lines treated with different concentrations (0.25 microM, 1 microM, 4 microM) of MM1, MM2, or imetelstat over a 4-week period. Data pooled from three technical replicates per condition from *n* = 2 independent experiments. Log2 fold changes of cell growth (celltiter-assay) of drug-treated conditions compared to vehicle controls are presented as heatmap. **b**, Median fluorescent intensities of anti-DNA G-quadruplex antibody (1H6) in editing-control (*n* = 5 biological replicates, that is native, Cas9, empty vector, CD33-sg1, CD33-sg2) NB4 cell lines treated with PBS, MM1, MM2, or imetelstat, and gated on G1 (left), or S/G2/M cell cycle phases (right). Each dot represents the mean of three technical replicates from one representative out of three independent experiments. **c**, CellROX measurement of *n* = 5 editing controls or *n* = 2 *FADS2*-edited (FADS2-sg1, FADS2-sg2) NB4 treated with PBS, MM1, MM2, or imetelstat. Each dot represents the mean of three technical replicates per cell line from one representative out of four independent experiments. **b,c**, One-way-ANOVA analysis adjusted for multiple comparisons: *P* < 1 × 10^−4^ (PBS versus MM2 in G1 and S/G2/M; **b**), *P* < 1 × 10^−4^ (PBS versus imetelstat in G1 and S/G2/M; **b**), *P* < 1 × 10^−4^ (editing controls+ PBS versus MM1; **c**), *P* < 1 × 10^−4^ (editing controls + PBS versus MM2; **c**), *P* < 1 × 10^−4^ (editing controls + PBS versus imetelstat; **c**), *P* = 2 × 10^−4^ (MM2-treated editing controls versus FADS2-edited cells; **c**), *P* < 1 × 10^−4^ (imetelstat-treated editing controls versus FADS2-edited cells; **c**). **d**, Celltiter analysis on NB4 treated with PBS, MM1, MM2 or imetelstat, and in combination with ferrostatin-1 (left) or DFOM (right). *N* = 3 technical replicates per condition from one representative out of two independent experiments in total. One-way-ANOVA adjusted for multiple comparisons: *P* < 1 × 10^−4^ (PBS versus MM2), *P* < 1 × 10^−4^ (PBS versus imetelstat). **e**, Peripheral blood AML burden in AML PDX (RCH-11) treated with PBS (*n =* 6), MM1, (*n* = 5), MM2 (*n* = 5), or imetelstat (*n* = 6). One-way-ANOVA adjusted for multiple comparisons on day 17: *P* = 1.83 × 10^−2^ (PBS versus MM2), *P* = 1.2 × 10^−3^ (PBS versus imetelstat). Source data

Recent independent work demonstrated the capacity of imetelstat to form G-quadruplex structures in vitro and this capacity is attributed to the presence of a triple G-repeat (GGG) in its sequence^25^. These insights prompted us to obtain an additional mismatch control harboring a similar triple G-repeat, but containing enough mismatches to prevent efficient binding to telomerase (Extended Data Fig. 7a). Using an antibody raised against (T4G4)2 intermolecular G-quadruplex DNA structures^26–28^, we found that imetelstat or GGG-containing mismatch but not mismatch 1 significantly interfered with endogenous DNA G-quadruplex structures (Extended Data Fig. 7b). In a panel of 14 human hematopoietic cell lines, GGG-containing mismatch control and imetelstat demonstrated similar efficacies in the majority of AML cell lines tested (Extended Data Fig. 7a). Moreover, GGG-containing mismatch was similarly effective as imetelstat in increasing ROS levels when compared to vehicle control (Extended Data Fig. 7c). Ferrostatin- or deferoxamine mesylate-mediated inhibition of ferroptosis rescued both imetelstat as well as GGG-mismatch-induced cell death (Extended Data Fig. 7d). We next compared the preclinical efficacy of imetelstat with GGG-mismatch and mismatch 1 in an *NRAS/KRAS*-mutant AML PDX model (RCH-11). In this model, GGG-mismatch was also effective in reducing AML burden (Extended Data Fig. 7e).

In addition to binding telomeric G-quadruplexes, vimentin has long been established as structural component of lipid droplets regulating their biogenesis and stability.

Lipid droplets can undergo selective autophagy (lipophagy) that can result in the induction of ferroptosis^29^. We hypothesized that imetelstat-induced PUFA phospholipid synthesis, oxidation and ferroptosis can result from lipophagy. Vimentin was highly expressed at protein level in AML cells in vitro (Fig. 6b) and loss-of-function editing of vimentin resulted in a modest competitive growth advantage of AML cells under imetelstat pressure (Fig. 6c). We next assessed lipophagy using C12-BODIPY, a fluorescent fatty acid probe for lipid droplets, in conjunction with the late endosomal marker LAMP1 (ref. ^30^). Imaging flow cytometry revealed significantly increased colocalization of lipid droplets with the late endosomal marker LAMP1, indicating increased lipophagy (Fig. 6d). To test whether pharmacological inhibition of lipophagy can prevent imetelstat-induced ferroptosis, we cultured AML cells in the presence of imetelstat combined with chloroquine, which inhibits lysosomal hydrolases by increasing the pH and thus lipophagy. Notably, in all AML cell lines tested, chloroquine diminished imetelstat-induced cell death (Fig. 6e).

These results provide evidence for a role of lipophagy-induced ferroptosis in imetelstat’s mechanism of action in AML via impaired lipid droplet homeostasis due to G-quadruplex mediated interference with the structural components of lipid droplets.

### Oxidative stress signatures distinguish sustained responders

**Extended Data Fig. 8:**
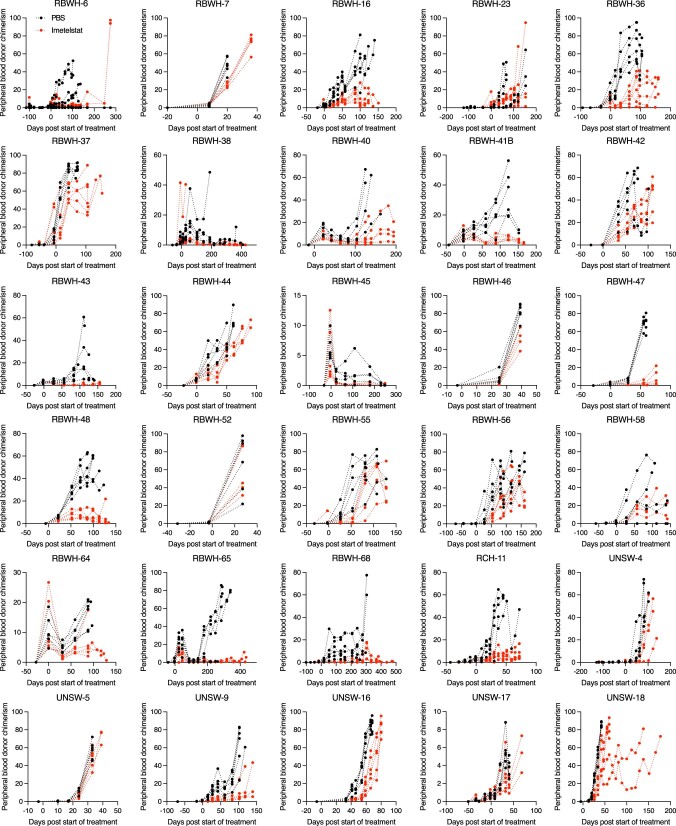
Individual AML PDX responses to imetelstat. Peripheral blood AML donor chimerism in the thirty individual PDX models. *N* = 6 NSGS per treatment group per individual AML patient sample. Kinetics are presented from each individual replicate. Source data

**Extended Data Fig. 9:**
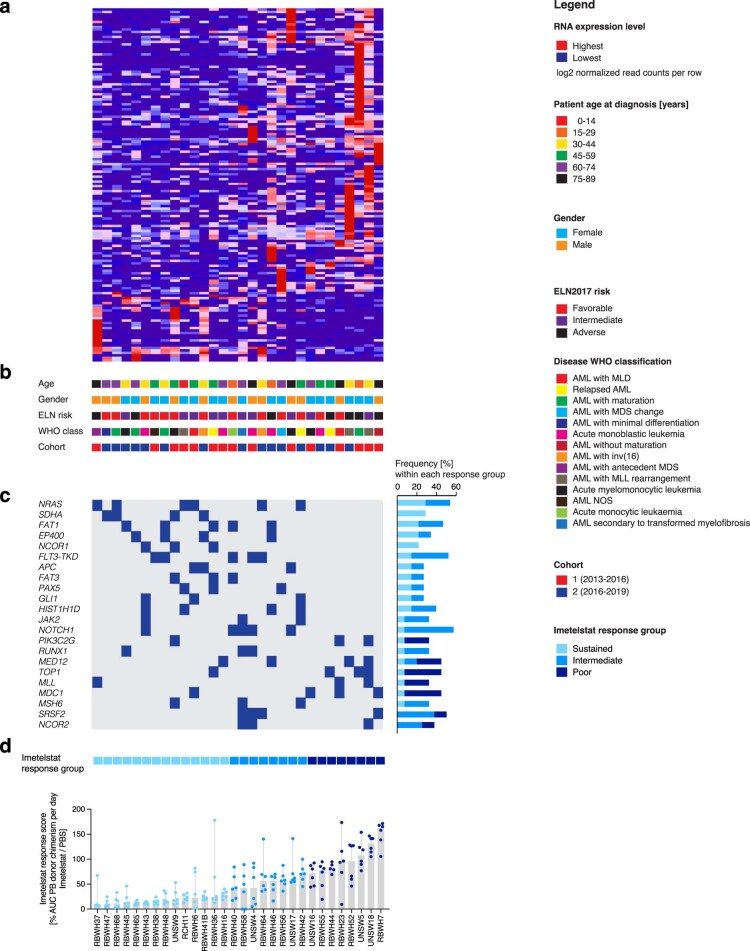
The molecular landscapes underlying imetelstat responses in AML PDX. **a**, Unsupervised hierarchical clustering analysis on the expression of differentially expressed transcripts in sustained versus poor responders to imetelstat determined using glmFit function (R) with a cutoff of adjusted *P* value < 0.25 among 30 individual AMLs from our PDX repository. **b**, Key clinical characteristics of patients from whom AML samples were derived including age at diagnosis, gender, ELN2017 prognostic risk group, and WHO class of disease. **c**, OncoPrint of differentially detected mutations in AMLs from sutained versus poor responders to imetelstat at baseline by targeted next generation sequencing of 585 genes associated with hematologic malignancies (the MSKCC HemePACT assay)^62^. **d**, Imetelstat response classification: AML burden quantified as area under the curve of peripheral blood donor chimerism per day of imetelstat / vehicle (PBS)-treated AML PDX generated from each individual AML patient sample. *N* = 30 AML patient samples with *n* = 6 PDX per treatment group. Source data

**Extended Data Fig. 10:**
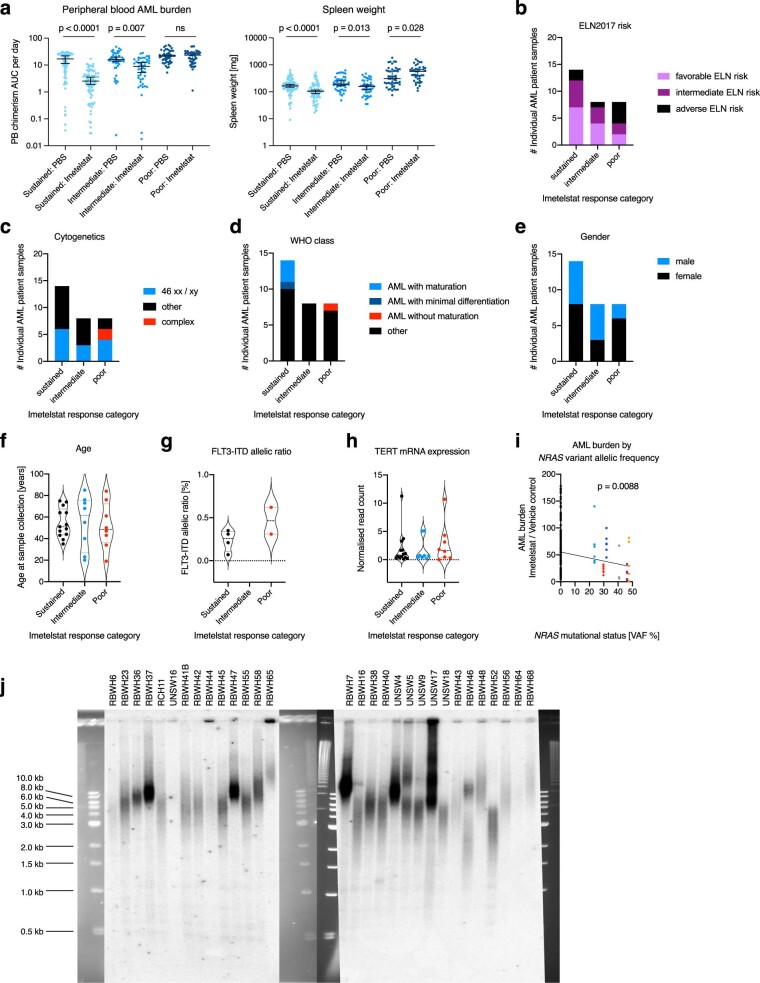
Imetelstat response classification in AML PDX. **a**, Imetelstat response classification: AML burden quantified as peripheral blood donor chimerism per day and spleen weights from imetelstat vs. vehicle control-treated PDX by imetelstat response group. *N* = 84 (sustained responders per treatment group each); *n* = 48 (intermediate or poor responders per treatment group each). Solid lines represent the median from each group ± 95% confidence interval. Statistics based on unpaired two-sided t-test on log-transformed data: *P =* 1.32 × 10^−11^ (PB AML burden in sustained responders: PBS vs. imetelstat), *P* = 6.68 × 10^−3^ (PB AML burden in intermediate responders: PBS vs. imetelstat), *P* = 8.82 × 10^−5^ (spleen weight in sustained responders: PBS vs. imetelstat), *P* = 1.34 × 10^−2^ (spleen weight in interdediate responders: PBS vs. imetelstat), *P* = 2.8 × 10^−2^ (spleen weight in poor responders: PBS vs. imetelstat). **b-h**, Representation of clinical and molecular parameters in AML patient samples either characterized as sustained, intermediate, or poor responders to imetelstat: ELN2017 risk (**b**), cytogenetics (**c**), WHO disease classification (**d)**, gender (**e**), AML patient age at sampling (**f**), FLT3-ITD allelic ratio (**g**), and TERT mRNA expression levels at baseline from RNA-seq analysis (**h**). Results were not statistically different (that is according to two-tailed Fisher’s exact test for **b-e**; one-way-ANOVA for **f-h**). *N* = 14 sustained, n = 8 intermediate, n = 8 poor responders to imetelstat. **i**, AML burden in imetelstat-treated normalized to vehicle control-treated PDX in relation to *NRAS* mutational status: *NRAS* wild-type (wt; *n* = 144), mutant *NRAS* (mut; *n* = 36). Simple linear regression indicates the slope being significantly different from 0 with *p* = 8.8 × 10^−3^. **j**, Telomeric restriction fragment analysis of genomic DNA isolated from viable AML patient cells at baseline from all 30 individual AML patient samples included in the preclinical trial of imetelstat in AML PDX. Source data

We next aimed to identify biomarkers of imetelstat response and resistance. Improved survival in imetelstat-treated AML PDXs correlated with significantly reduced engraftment and disease burden; however, there were clear differences in the magnitude and duration of individual responses (Extended Data Fig. 8). To understand determinants of imetelstat response, we allocated each individual AML patient sample into either sustained, intermediate or poor imetelstat response categories based on the individual effect of imetelstat on AML burden measured in peripheral blood over time (Extended Data Figs. 9 and 10a). All ELN2017 prognostic risk categories were represented in each imetelstat response group, suggesting that the effects observed were not solely explained by favorable disease (Extended Data Fig. 10b). In addition, cytogenetics, sex, age, *FLT3-ITD* allelic ratio and TERT messenger RNA expression levels at baseline seemed similar among imetelstat response groups (Extended Data Fig. 10c–h).

**Fig. 7:**
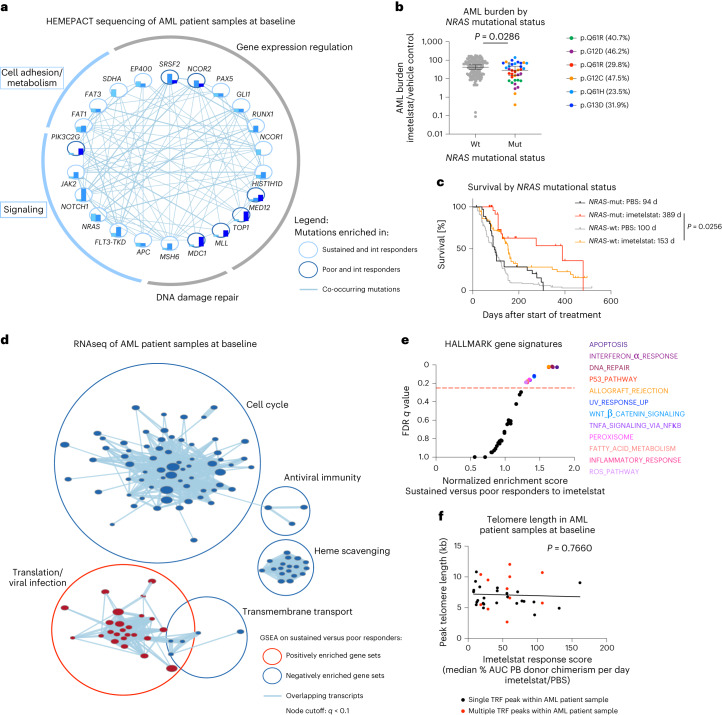
Mutant *NRAS* and oxidative stress gene expression signatures associate with sustained responses to imetelstat. Segregation of samples from patients with AML into sustained, intermediate and poor imetelstat response groups based on PB AML burden with *n* = 14 (sustained), *n* = 8 (intermediate) and *n* = 8 (poor). **a**, Cytoscape visualization of the frequencies of genes with oncogenic mutations (based on the COSMIC database^79^) in sustained (turquoise), intermediate (light blue) and poor (dark blue) responders to imetelstat. Connecting lines represent co-occurring mutations within the same AML patient sample. **b**, AML burden in imetelstat-treated normalized to vehicle control-treated PDXs in relation to *NRAS* mutational status. *NRAS* wild-type (wt; *n* = 144 PDXs) and mutant *NRAS* (mut; *n* = 36 PDXs). Statistics were conducted according to a two-sided *t*-test on log-transformed data: *P* = 2.86 × 10^−2^. **c**, Two-tailed survival analysis of PBS and imetelstat-treated AML PDXs divided into groups based on their *NRAS* mutation status. Median survival was 94 (PBS-treated *NRAS*-mut; *n* = 36 PDXs), 389 (imetelstat-treated *NRAS*-mut; *n* = 36 PDXs), 100 (PBS-treated *NRAS*-wt; *n* = 144) and 153 (imetelstat-treated *NRAS*-wt; *n* = 144) days from start of treatment. *P* = 2.56 × 10^−2^ comparing imetelstat-treated *NRAS*-mut to imetelstat-treated *NRAS*-wt PDX according to Gehan–Breslow–Wilcoxon. **d**, Cytoscape visualization of GSEA results on RNA-seq data from individual AML patient samples at baseline comparing sustained with poor responders to imetelstat (*n* = 14 sustained responders; *n* = 8 poor responders; node cutoff, *q* < 0.1). Red circles represent gene sets positively enriched in sustained versus poor responders to imetelstat. Blue circles represent negatively enriched gene sets in sustained versus poor responders to imetelstat. **e**, Hallmark GSEA on RNA-seq data comparing sustained versus poor responders to imetelstat at baseline. The red dotted line represents the cutoff considered for significant enrichment at FDR = 0.25. **f**, Simple linear regression analysis of baseline telomere length versus imetelstat response in PDXs. *n* = 30 AML patient samples. *P* = 7.66 × 10^−1^; *F* = 8.998 × 10^−1^; degrees of freedom numerator, degrees of freedom denominator = 1, 34; slope 95% CI (−2.219 × 10^−1^, 1.648 × 10^−1^); *y* intercept 95% CI (6.023, 8.449); *x* intercept 95% CI (367.9, +infinity). *R*^2^ = 2.639 × 10^−3^. Source data

We next aimed to identify genetic biomarkers of response and resistance to imetelstat therapy by analyzing the data from individual samples from patients with AML at baseline that were generated by genomic sequencing using a comprehensive panel of 585 genes frequently mutated in hematological malignancies^31^ (Extended Data Fig. 9c). Oncogenic mutations in genes annotated in signaling or cell adhesion/metabolism were trend-wise more frequently observed in sustained compared to poor responders to imetelstat (Fig. 7a and Extended Data Fig. 9c).

Mutant *NRAS* was associated with enhanced responses to imetelstat therapy. This was evidenced by reduced AML burden and improvement in survival when compared to wild-type *NRAS* containing AML PDXs (Fig. 7b,c). Moreover, variant allelic frequencies of the relevant *NRAS* mutations inversely correlated with AML burden in imetelstat-treated AML PDXs (Extended Data Fig. 10i). Additionally, gene set enrichment analysis (GSEA) of the RNA-seq data obtained from the individual samples from patients with AML at baseline revealed that sustained responders had a positive enrichment of gene signatures associated with translation/viral infection and negative enrichment for gene signatures associated with cell cycle, antiviral immunity, transmembrane transport and heme scavenging compared to poor responders to imetelstat (Fig. 7d and Extended Data Fig. 9a). Hallmark signatures revealed significant enrichment of gene sets annotated as apoptosis, interferon-α response, DNA repair, TP53 pathway, peroxisome, fatty acid metabolism and ROS pathway in sustained compared to poor responders to imetelstat (Fig. 7e).

We next examined whether baseline telomere length could predict imetelstat response. Telomere length was determined by telomere restriction fragment (TRF) analyses and peak telomere lengths varied between 2.7 and 12 kb among individual AML patient samples (Fig. 7f and Extended Data Fig. 10j). Five out of the 30 AML patient samples contained multiple subclones with distinct telomere lengths (Fig. 7f and Extended Data Fig. 10j). Overall, there was no correlation between baseline telomere length and imetelstat response (Fig. 7f).

These data demonstrate that imetelstat is effective in a large proportion of AML PDXs. Furthermore, sustained responses to imetelstat are independent of baseline telomere length and are associated with marked improvements in survival, mutant *NRAS* and baseline molecular signatures annotated as oxidative stress.

### Oxidative stress induction sensitizes AML PDX to imetelstat

The finding that responses to imetelstat are associated with baseline molecular signatures annotated as oxidative stress and that the mechanism of action of imetelstat features ROS-mediated ferroptosis led to the hypothesis that oxidative stress induction can sensitize to imetelstat therapy.

**Fig. 8:**
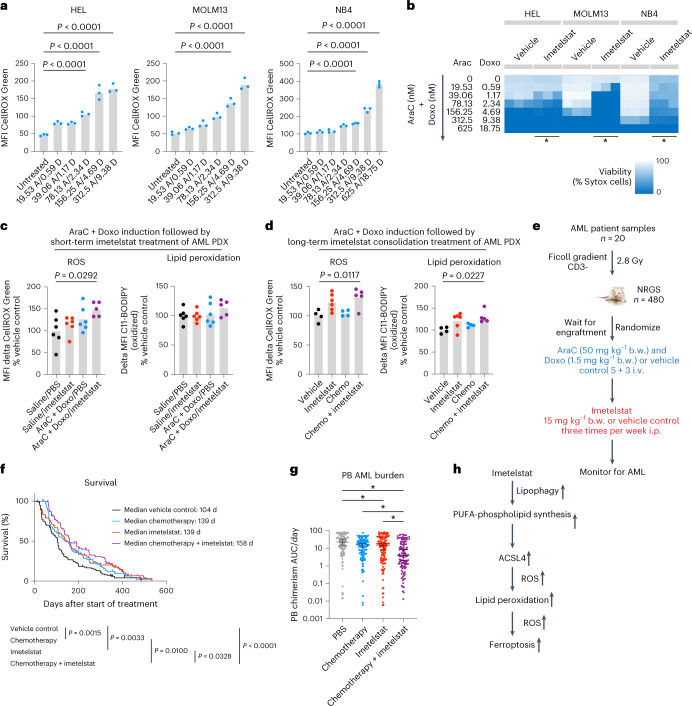
Oxidative stress induction with standard chemotherapy to sensitize AML cells to imetelstat. **a**, CellROX flow cytometry on AML cells treated with various concentrations of AraC + Doxo after 3 d in culture. *n* = 3 replicates from a representative experiment that was repeated independently showing similar results. Data are presented as mean MFI ± s.e.m. One-way ANOVA adjusted for multiple comparisons, *P* < 1 × 10^−4^ (HEL); *P* < 1 × 10^−4^ (MOLM13); *P* < 1 × 10^−4^ (NB4). AraC, cytarabine; Doxo, doxorubicin. **b**, Sytox flow cytometry on cultures after switching to imetelstat (4 μM). Heat maps represent viabilities (Sytox cell percentages). Asterisks (*) denote statistically significant differences (*P* < 5 × 10^−2^) between imetelstat-treated cells pretreated with 1.17 nM AraC + 39.06 nM Doxo (*n* = 3) versus imetelstat-treated controls that were not pretreated (*n* = 3) according to an unpaired two-sided *t*-test, *P* = 1.47 × 10^−2^ (HEL); *P* = 8.43 × 10^−6^ (MOLM13); *P* = 1.22 × 10^−6^ (NB4). **c**,**d**, C11-BODIPY and CellROX analysis on splenic CD45^+^ cells from PDXs (RBWH-44). Data are presented as mean ± s.e.m. One-way ANOVA adjusted for multiple comparisons. **c**, PDX received one dose of AraC + Doxo on day 1 followed by one dose of imetelstat on day 2 and were analyzed on day 3. AraC + Doxo + imetelstat (*n* = 5) versus vehicle (*n* = 6), *P* = 2.92 × 10^−2^ (MFI CellROX). **d**, PDX received a 5 + 3 AraC + Doxo cycle followed by imetelstat consolidation and were analyzed after 3 months. AraC + Doxo + imetelstat (*n* = 5) versus vehicle (*n* = 4), *P* = 1.117 × 10^−2^ (CellROX), *P* = 2.27 × 10^−2^ (C11-BODIPY). **e**–**g**, PDX trial on imetelstat consolidation following induction chemotherapy. Experimental scheme (**e**), survival (**f**) and PB AML burden (**g**). *n* = 120 PDXs per treatment group. i.v., intravenous; i.p., intraperitoneal; b.w., body weight. **f**, Two-tailed Kaplan–Meier analysis according to Gehan–Breslow–Wilcoxon, *P* = 1.5 × 10^−3^ (vehicle versus AraC + Doxo); *P* = 3.3 × 10^−3^ (vehicle versus imetelstat), *P* = 1 × 10^−2^ (AraC + Doxo versus AraC + Doxo + imetelstat); *P* = 3.28 × 10^−2^ (imetelstat versus imetelstat + AraC + Doxo); *P* < 1 × 10^−4^ (vehicle versus AraC + Doxo + imetelstat). **g**, One-way ANOVA adjusted for multiple comparisons on log-transformed data. Asterisks (*) denote statistically significant differences, *P* = 2.3 × 10^−2^ (vehicle versus imetelstat); *P* = 2.2 × 10^−3^ (AraC + Doxo versus chemotherapy + imetelstat); *P* = 4.14 × 10^−2^ (imetelstat versus AraC + Doxo + imetelstat), *P* < 1 × 10^−4^ (vehicle versus AraC + Doxo + imetelstat). **h**, Model demonstrating the working hypothesis on imetelstat-induced ferroptosis in AML derived from this study. Source data

Standard induction chemotherapy composed of cytarabine and an anthracycline is a potent inducer of ROS^32^. To test whether oxidative stress-inducing therapy can sensitize AML cells to imetelstat treatment, we pretreated AML cell lines with oxidative stress-inducing standard induction chemotherapy (cytarabine in combination with doxorubicin) and subsequently switched to imetelstat treatment. Standard induction chemotherapy significantly increased ROS levels in a dose-dependent manner that led to augmented cell death in AML cell lines (Fig. 8a,b).

In a pilot study using an *NRAS* wild-type AML PDX model (poor responder to imetelstat monotherapy; RBWH-44), a single dose of standard induction chemotherapy followed by a single dose of imetelstat resulted in significantly increased ROS levels in AML patient-derived cells in PDXs in vivo (Fig. 8c). At this early time point, lipid peroxidation was not significantly different between the treatment groups (Fig. 8c); however, after a complete cycle of induction chemotherapy followed by prolonged treatment with imetelstat consolidation therapy, both lipid peroxidation and ROS levels were significantly increased (Fig. 8d).

Finally, as a proof of concept in vivo, we sequentially administered oxidative stress-inducing standard induction chemotherapy before imetelstat in a diverse PDX cohort from 20 distinct AML patient samples (Fig. 8e). Combination therapy significantly prolonged survival when compared to imetelstat monotherapy (158 d versus 139 d; *P* = 0.0328), induction chemotherapy alone (158 d versus 139 d, *P* = 0.0100) or vehicle control (158 d versus 104 d, *P* < 0.0001; Fig. 8f). AML burden was significantly reduced in the combination therapy group when compared to either monotherapy or vehicle-treated control groups (Fig. 8g).

These data demonstrate that the rational sequencing of imetelstat and chemotherapy, using standard induction chemotherapy to induce oxidative stress and sensitize AML cells to imetelstat-induced lipid peroxidation and ferroptosis, results in significantly improved disease control of AML (Fig. 8h).

## Discussion

Imetelstat is a first-in class telomerase inhibitor with clinical efficacies in hematological myeloid malignancies, including essential thrombocythemia, myelofibrosis and lower-risk myelodysplastic syndromes^6–8^. The efficacy of imetelstat in AML and its mode of action have remained elusive to date. By developing and utilizing a comprehensive AML PDX resource and human cell lines for genomics, transcriptomics and lipidomics approaches combined with functional genetic and pharmacological validation experiments, we demonstrate that imetelstat is a potent inducer of ferroptosis that effectively diminishes AML burden and delays relapse following chemotherapy.

Ferroptosis is a recently discovered type of non-apoptotic regulatory cell death that relies on the balance of the production of ROS during lipid peroxidation and the antioxidant system and it is generally characterized by three hallmarks: (1) loss of peroxide repair capacity through GPX4; (2) availability of redox-active iron; and (3) oxidation of polyunsaturated fatty acid-containing phospholipids^22,33^. The experiments performed in this study have revealed evidence for imetelstat directly affecting the third hallmark of ferroptosis; the increased synthesis and subsequent oxidation of PUFA phospholipids. In AML PDXs in vivo, imetelstat-induced lipid peroxidation is associated with significantly increased *ACSL4* expression. In human AML cell lines, imetelstat treatment significantly increased lipid ROS levels that preceded massive cell death. Treatment with the lipid ROS scavengers ferrostatin-1 or liproxstatin-1 rescued imetelstat-induced cell death in all AML cell lines tested. In addition, pharmacological iron chelation using deferoxamine mesylate, 5-lipoxygenase inhibition using zileuton or menadione supplementation were able to prevent imetelstat-induced cell death in a substantial proportion of AML cell lines tested. In contrast to ferrostatin-1 and liproxstatin-1, higher concentrations of deferoxamine mesylate were detrimental for AML cells, suggesting that iron availability is crucial for AML cell survival at a level specific for each cell line. Iron metabolism is altered in AML at the cellular and systemic level and elevated iron levels help to maintain the rapid growth rate of AML cells by activating ribonucleotide reductase that catalyzes DNA synthesis in an iron-dependent manner^34^. Notably, imetelstat has shown efficacy in patients with pathology featuring ringed sideroblasts^7,8,35^, a cellular morphological abnormality that is defined by iron-laden granules in mitochondria surrounding the nucleus, further supporting the role of iron-dependent cell death.

Our functional genetic experiments using Brunello library CRISPR/Cas9 editing have provided further evidence that imetelstat restricts leukemic progression via ferroptosis, revealing a closely related functional network of seven genes. One of the identified targets, ACSL4, has previously been identified as a key regulator of ferroptosis sensitivity through the shaping of the cellular lipid composition^21^. We have functionally validated the most significantly enriched targets, FADS2 and ACSL4. The canonical role of FADS2 in fatty acid metabolism is the catalysis of the desaturation of linoleic and α-linolenic acid to long-chain PUFAs^36,37^. Lipidomics analysis has revealed increased levels of phospholipids containing triglycerides and also increased levels of phospholipids containing PUFAs with three unsaturated bonds in imetelstat-treated AML cells in an FADS2-dependent manner. Using a fluorescent sensor, we have confirmed that imetelstat stimulates lipid peroxidation. These data demonstrate imetelstat-induced alterations in fatty acid metabolism that promote the formation of substrates for lipid peroxidation. Of note, in some lung cancer cell lines, FADS2 activation is associated with ferroptosis suppression^38^. This dichotomy may be explained by the fact that in some cancer cells, FADS2 enables the desaturation of palmitate to sapienate (*cis*-6-C16:1) as part of an alternative desaturation pathway, thus potentially reducing the levels of monounsaturated fatty acids and ultimately PUFA-containing phospholipids as substrates for lipid peroxidation^39^.

G-quadruplexes are recognized by and regulate the activity of many proteins involved in telomere maintenance, replication, transcription, translation, mutagenesis and DNA recombination^40^. The recognition of G-quadruplexes can be dictated by R-loops that show a close structural interplay and can modulate responses involving DNA damage induction, telomere maintenance and alterations in gene expression regulation^41^. Notably, G-quadruplex/R-loop hybrid structures were detected in vitro in the human *NRAS* promoter and at human telomeres^42–45^. R-loop binders and epigenetic R-loop readers have been recently linked to altered fatty acid metabolism and ferroptosis^46–48^. Moreover, constitutively activated RAS/MAPK signaling downstream of mutant NRAS is associated with enhanced sensitivity to ferroptosis^33,49,50^; however, the activity of this pathway alone is unlikely to be the sole determinant of ferroptosis sensitivity^51,52^. Our integrative analysis of transcriptomics with functional genetics data has identified imetelstat target candidates that were recently discovered as G-quadruplex-binding proteins (VIM and LMNA)^29^. Moreover, VIM and LMNA have been characterized as proteins directly interacting with lipid droplets^53,54^. Recent independent work has provided evidence for a role of lipid droplets in ferroptosis. In hepatocytes, the degradation of intracellular lipid droplets via autophagy (lipophagy) promotes RSL3-induced ferroptosis by decreasing lipid storage that subsequently induces lipid peroxidation^29^. Our imaging flow cytometry analysis demonstrates significantly increased colocalization of markers for lipid droplets and late endosomes, proposing imetelstat-induced lipophagy as trigger for ferroptosis in AML.

Using a newly established, comprehensive AML patient-derived xenograft resource that reflects the overall genetic abnormalities found in large clinical cohorts, we demonstrated a proof of concept for the sequential administration of standard induction chemotherapy followed by imetelstat consolidation to induce oxidative stress and sensitize AML patient samples to imetelstat treatment. This approach was able to cause significant delay or prevention of AML relapse. The efficacy of sequential therapy suggests that imetelstat may be particularly useful in preventing relapse after chemotherapy, for example, as a maintenance therapy. Recently, maintenance therapy with oral CC486 has shown a survival benefit in AML; however, there is no survival plateau and therefore, most patients still relapse and die of their disease^55^. A substantial proportion of AML patient samples tested (14 out of 30 samples) were classified as sustained responders to imetelstat monotherapy and are characterized by genetic lesions in genes involved in cell adhesion, metabolism and signaling, with the most striking result obtained for *NRAS. NRAS* is the fourth most commonly observed gene with driver mutations in adult AML^2^. Moreover, AML cell clones harboring mutant *NRAS* arise in some patients relapsing on targeted therapies, particularly FLT3 inhibition (crenolanib^56^ and gilteritinib^57^) and BCL2 inhibition in some cases (venetoclax^58,59^). The demonstrated sustained responses to imetelstat in *NRAS*-mutant AML patient samples raise the possibility that imetelstat may be used as salvage therapy or possibly in combination with FLT3 inhibitors or venetoclax to prolong remission and prevent relapse.

In conclusion, imetelstat is a potent inducer of ferroptosis that effectively diminishes AML burden and delays relapse following oxidative stress-inducing chemotherapy.

Clinical trials will address the efficacy of imetelstat in AML and may focus on this compound as a consolidation strategy for preventing relapse or potentially together with targeted therapies to improve outcomes in patients with AML.

## Methods

Our research complies with all relevant ethical regulations, including QIMR Berghofer human research ethics committee protocol P1382 (HREC/14/QRBW/278) and QIMR Berghofer animal research ethics committee protocol A11605M. Animals were monitored daily and immediately killed based on the scoring criteria detailed below.

### Mouse monitoring

Animals were monitored daily and always immediately killed as soon as a cumulative clinical score of 3 or above was reached, based on weight loss (score 1, >10–20%; and score 2, >20% or >15% if maintained for >72 h), posture (score 1, hunching noted only at rest; and score 2, severe hunching), activity (score 1, mild to moderately decreased; and score 2, stationary unless stimulated, hind limb paralysis) and white cell count (score 1, 10–60 × 10^6 ^ml^−1^; and score 2, >60 × 10^6 ^ml^−1^).

### Mouse models

All mouse experiments were approved by the institutional (QIMR Berghofer) ethics committee protocol A11605M. NSG (NOD.Cg-Prkdcscid Il2rgtm1Wjz/SzJ), NSGS (NOD.Cg-Prkdcscid Il2rgtm1Wjl Tg[CMV-IL3,CSF2,KITLG]1Eav/MloySzJ) and NRGS (NOD.Cg-Rag1tm1Mom Il2rgtm1Wjl Tg[CMV-IL3,CSF2,KITLG]1Eav/J) were imported from Jackson Laboratories. All mice were kept pathogen-free in the animal facility of QIMR Berghofer. Mice received autoclaved Baytril-treated (100 mg l^−1^; Provet) water until 1–7 d before irradiation and after that, autoclaved Septrin-treated (12 ml l^−1^ pediatric suspension, 96 mg l^−1^ trimethoprim and 480 mg l^−1^ sulfamethoxazole; Arrow Pharmaceuticals) water. Please refer to the Supplementary Note for detailed animal housing and feeding conditions.

### Xenograft transplantation experiments

AML samples were obtained from patients, after informed consent in accordance with the Declaration of Helsinki. Ficoll density gradient was used to recover viable mononuclear cells. Viably frozen AML cells were thawed and CD3-depleted with biotinylated anti-human CD3 (SK7) and biotin-binder Dynabeads (Invitrogen) and subsequently injected via the lateral tail vein into 2.8 Gy irradiated (24 h before transplant) female NSGS or NRGS recipients (6–8 weeks old). For normal hematopoiesis studies, viable mononuclear cells were isolated from cord blood samples (provided by the Wesley-St Andrew’s Research Institute Tissue Bank with appropriate ethics approval) by Ficoll density gradient, CD3-depleted as above and subsequently enriched for CD34^+^ cells using the human CD34 MicroBead kit (130-046-702 MACS Miltenyi Biotec). A total of 56,000 cells (donor 1) or 212,500 cells (donor 2) were injected via the lateral tail vein per irradiated female NSG recipient (6–8 weeks old).

### Oligonucleotide sequences of imetelstat and mismatch controls

Imetelstat (GRN163L): 5′ R-TAGGGTTAGACAA-NH2 3′.

Mismatch 1 (GRN140833): 5′ R-TAGGTGTAAGCAA-NH2 3′.

Mismatch 2 (GRN142865): 5′ R-TAGGGATTCAGAA-NH2 3′.

### Drug treatment studies

NSG, NSGS or NRGS mice were treated with 15 mg kg^−1^ imetelstat (GRN163L), mismatch controls (mismatch 1 also referred to as MM1 or GRN140833; mismatch 2 also referred to as GGG-mismatch, MM2 or GRN142865) or vehicle control (PBS) via the i.p. route for the period of time specified in the respective experiment three times per week, at least every 72 h. For standard induction chemotherapy studies, cytarabine (AraC; 1 g in 10 ml isotonic water; Pfizer) and doxorubicin (Doxo; 50 mg in 25 ml saline; Pfizer) were freshly diluted with saline (sodium chloride 0.9% for irrigation; Baxter) to achieve a final concentration of 50 mg kg^−1^ body weight AraC or 1.5 mg kg^−1^ body weight Doxo in 200 μl total injection volume per recipient. Both AraC and Doxo were co-delivered i.v. (in the same syringe) on days 1 to 3, followed by i.v. injection of AraC alone on days 4 and 5, each in strict 24-h intervals. For chemotherapy plus imetelstat combination studies, the first imetelstat injection was administrated 1 d after the standard induction chemotherapy cycle was completed. For in vivo liproxstatin-1 treatment studies, liproxstatin-1 (SEL-S7699; Jomar Life Research) was dissolved in dimethylsulfoxide (7.9 mg in 400 μl) and then diluted with 2.44 ml 0.9% NaCl (saline). Liproxstatin-1 (15 mg kg^−1^) was administered by i.p. injection via a 27 G insulin needle twice daily for 2 weeks (200 μl per recipient).

### Blood analysis

Blood was collected into EDTA-coated tubes and analyzed on a Hemavet 950 (Drew Scientific). PB smears were stained with Wright-Giemsa according to the manufacturer’s protocol (BioScientific).

### Histology

Tissues were fixed in 10% neutral buffered formalin, embedded in paraffin and stained with hematoxylin and eosin. Images of histological slides were obtained on a ScanScope FL (Aperio).

### Flow cytometry analysis of AML PDX and cord blood transplants

For monitoring AML engraftment, 25–50 μl of PB were stained after red cell lysis (BD Pharm Lyse, BD Biosciences) with anti-human CD45-AF647 (H130) and anti-mouse CD45.1-PE (A20). For AML phenotyping, cell populations were purified from bone marrow (both femurs and tibiae) or SPL and after red blood cell lysis stained with anti-human CD45-FITC (H130), anti-mouse Cd45.1-PerCP/Cy5.5 (A20), anti-human CD34-PE (581), anti-human CD33-APC (WM53), anti-human CD38-APC/Cy7 (HIT2) and anti-human GPR56-PE/Cy7 (CG4). Flow cytometry analysis of lipid peroxidation was performed using C11-BODIPY 581/591 (Sapphire Bioscience) according to a previously published protocol^60^ and ROS were quantified using CellROX Green (Invitrogen) according to the manufacturer’s instructions, subsequent to cell surface marker staining. In all analyses, dead cells were discriminated by Sytox Blue (Invitrogen). All antibodies were used as 1:100 dilutions with a maximum concentration of 1 × 10^6^ cells per 100 μl. Washes and staining were performed in PBS + 2% FCS + 1 mM EDTA. Centrifugation steps were performed at 300*g* for 10 min at 4 °C. Flow cytometry analysis was performed on a FACS LSRFortessa (BD Biosciences). Post-acquisition analyses were performed with FlowJo software v.10.9.0 (Becton Dickinson & Company; BD). The Supplementary Note contains a detailed description of the flow cytometry analysis of cord blood transplants.

### Terminal restriction fragment analysis

TRFs were obtained from genomic DNA by complete digestion with the restriction enzymes HinfI and RsaI. TRFs were separated by pulsed-field gel electrophoresis. Gels were dried, denatured and subjected to in-gel hybridization with a γ-[32P]-ATP-labeled (CCCTAA)4 oligonucleotide probe. Gels were washed and the telomeric signal visualized by phosphorimage analysis. TRFs were processed by ImageJ 1.52a analysis software to quantitate mean telomere length.

### Telomere length qPCR

Samples were purified using the DNeasy Blood and Tissue kit (QIAGEN). DNA isolation was performed as described previously^61^, including degassing of buffers and supplementation with 50 μM of phenyl-tert-butyl nitrone to minimize oxidative damage. Telomere length was assessed using qPCR^62–64^. The Supplementary Note contains further details of the procedure.

### Cell lines

The Supplementary Note contains purchasing details of the human cell lines used in this study. All cell lines were authenticated by STR profiling at an early passage before the first culture experiment, performed by the QIMR Berghofer Analytical Core facility. None of the cell lines used has been known as misidentified cell lines according to v.12 of the cross-contamination database maintained by the International Cell Line Authentication Committee (https://iclac.org/databases/cross-contaminations/). All cell lines tested negative for *Mycoplasma* during regular monthly testing using the biochemical MycoAlert™ Mycoplasma Detection kit (Lonza) by QIMR Berghofer core facility.

### Cell culture and in vitro cell growth analysis

AML cell lines were cultured in RPMI with 10% fetal calf serum, 2 mM glutamine and 200 U ml^−1^ penicillin and 200 μg ml^−1^ streptomycin. All cell culture experiments were performed on low-density pre-cultures, passaged between 12–24 h before seeding at a density of ~1 × 10^5^ cells per ml, with a maximum density of 5 × 10^5^ cells per ml when cells were taken for experimental setup. AML cells were seeded into flat-bottom 96-well plates at a density of 2,500 cells per 100 μl. Every 48–72 h, 25 or 50 μl of the cultures were transferred into a new plate, depending on the density of each cell line in the control condition and supplemented with fresh medium containing imetelstat (GRN163L), mismatch controls or additional drugs of interest (ferrostatin-1 (Sigma-Aldrich), liproxstatin-1 (Sigma-Aldrich), deferoxamine mesylate (Hospira), zileuton (Sigma-Aldrich), menadione (Sigma-Aldrich), (+)-etomoxir sodium salt hydrate (Sigma-Aldrich), 1S,3R-RSL3 (Sigma-Aldrich) or erastin (Sigma-Aldrich)). Cells were analyzed with CellTiter 96 aqueous nonradioactive cell proliferation assay (MTS Systems) according to the manufacturer’s instructions (Promega). End point absorbance at 490 nm was detected using Biotek PowerWave and Gen5 data analysis software. Drug synergy scores were computed using the SynergyFinder v.2.0 algorithm (https://synergyfinder.fimm.fi/).

### Imaging flow cytometry

Lipophagy was detected by assessing colocalization of C12-BODIPY and LAMP1 using a previously published method with modifications^65^. In detail, 1 × 10^6^ cells were collected and washed in warm PBS (without FCS). Cells were then resuspended in warm RPMI (without FCS) containing 200 ng C12 FL BODIPY (Thermo Fisher Scientific) per ml and incubated for 30 min at 37 °C. Cells were then washed in 9 ml wash buffer (PBS + 2% FCS + 1 mM EDTA) and subsequently fixed and permeabilized using a FIX & PERM Cell Permeabilization kit (GAS-004; Invitrogen) and incubated with Alexa Fluor 647 anti-human CD107a (LAMP1; BioLegend; dilution 1:100) according to the manufacturer’s instructions. Cells were subsequently washed and resuspended in wash buffer with 0.2 mg ml^−1^ Hoechst 33342 (Invitrogen). The acquisition was performed using an Amnis ImageStream^X^ Mark II Imaging Flow and data were analyzed with IDEAS (Image Data Exploration and Analysis Software).

### Flow cytometry analysis of AML cell lines

Before staining, 2 × 10^5^ cells were washed with PBS with 2% FCS and 1 mM EDTA. For cell cycle and G-quadruplex analysis, cells were then fixed and permeabilized using a FIX & PERM Cell Permeabilization kit (GAS-004; Invitrogen) and incubated with anti-DNA G-quadruplex (G4) antibody, clone 1H6 (Merck Millipore; cat. MABE1126; dilution 1:100) for 30 min on ice, then washed, blocked for 25 min at room temperature in 1% BSA/2% FCS/PBS and subsequently stained with anti-IgG2b-FITC (1:100 dilution in 1%BSA/2%FCS/PBS) for 30 min on ice. Cells were resuspended in 2% FCS/PBS containing 0.2 mg ml^−1^ Hoechst 33342 (Invitrogen) before acquisition.

Flow cytometry analysis of lipid peroxidation was performed using C11-BODIPY 581/591 (Sapphire Bioscience) according to a previously published protocol^60^ and ROS were quantified using CellROX Green (Invitrogen) according to the manufacturer’s instructions. For both analyses, Sytox Blue 1.25 µM (S34857; Invitrogen) was used to distinguish between viable and dead cells. Flow cytometry analysis was performed on a FACS LSRFortessa (BD Biosciences). Post-acquisition analyses were performed with FlowJo software v.10.9.0 (BD).

### Western blotting

Cells were lysed on ice using m-PER lysis buffer (Thermo Fisher Scientific) supplemented with a protease and phosphatase inhibitor (Cell Signaling, 5872) and protein was quantified using Pierce BCA protein assay kit (Thermo Fisher Scientific). In total, 20–50 μg of protein extract was electrophoresed on a 4–15% SDS gradient gel (Mini-PROTEAN TGC Gel, Bio-Rad) and transferred to an activated PVDF membrane for 1 h at 4 °C. Unspecific binding sites were blocked in 5% BSA in Tris-buffered saline with 1% Tween-20 (TBS-T) for 1 h at 4 °C. Primary antibodies were incubated overnight at 4 °C in constant motion in 5% BSA–TBS-T. The mouse anti-Cas9 (S. pyogenes) antibody (Cell Signaling; cat. 14697, clone 7A9-3A3) and rabbit anti-ACSL4 antibody (Abcam; cat. ab155282; clone EPR8640) were used at 1:1,000 dilution in 5% BSA–TBS-T. The mouse anti-actin Ab-5 antibody (BD Biosciences; cat. 612656; clone C4/actin (RUO)) was used at 1:3,000 dilution in 5% BSA–TBS-T. The membrane was washed with TBS-T three times for 5 min before incubation with the secondary antibody for 1 h at room temperature. Polyclonal goat anti-rabbit immunoglobulins/HRP (Dako; cat. P0448) and polyclonal rabbit anti-mouse immunoglobulins/HRP (Dako; cat. P0260) were used at 1:4,000 dilution. After subsequent membrane washing, protein was detected using Immobilon chemiluminescent HRP substrate (WBKLS0500, Millipore) and imaged with the iBright CL1500 imaging system.

### Confocal microscopy

Cytospins were fixed and permeabilized with methanol:acetone (prechilled) at a ratio of 1:1 for 10 min at room temperature, then washed twice with cold PBS and once with room temperature PBS (5 min each). Cytospins were then incubated with 1% BSA–PBS at room temperature for 1 h, washed three times in PBS for 5 min each and then incubated in primary antibody (anti-human Vimentin XP rabbit monoclonal antibody Alexa Fluor 488 conjugate, Cell Signaling; cat. 9854; clone D21H3) or isotype control (rabbit monoclonal antibody IgG XP Isotype Control Alexa Fluor 488 conjugate, Cell Signaling; cat. 2975; clone DA1E) at a dilution of 1:400 in 1% BSA–PBS for 1 h at room temperature or O/N at 4 °C. Cytospins were then washed three times in PBS for 5 min each. Coverslips were mounted in pro-long DAPI Gold. Images were acquired on a Zeiss 780-NLO confocal microscope.

### CRISPR/Cas9 editing

The Brunello genome-wide gRNA library contains 76,441 gRNAs targeting 19,114 genes and was obtained from Addgene (cat. 73178)^66,67^. *Streptococcus* *Pyogenes* Cas9 and blasticidin resistance construct expressed from an EFS promoter (pFUGWb) was obtained from Addgene (lentiCas9-Blast, plasmid #52962)^68^. Lentivirus containing the Brunello library was generated and used to transduce NB4 cells. The Supplementary Note contains detailed descriptions of the CRISPR/Cas9 screen as well as sgRNA-mediated editing approaches.

### Mutational sequencing

Genomic alterations were profiled using the HemePACT assay (integrated mutation profiling of actionable cancer targets related to hematological malignancies)^31^. This assay uses solution phase hybridization-based exon capture and massively parallel DNA sequencing to capture all protein-coding exons and select introns of 585 actionable cancer related genes. Samples were molecularly barcoded, to allow optimal cost efficiency during the capture process as well as at the sequencing step. Then, 250 ng of genomic DNA was used for library construction. Pools of 12 samples equimolarly mixed were sequenced at the Genomics Core Laboratory at MSKCC, in one lane of a HiSeq 2500, using the SBS chemistry for paired-end 100/100 reads. The average coverage was greater than 400-fold, with a minimum of 99% of the targeted sequences covered 30-fold. Reads were aligned to the reference human genome (hg19) using the Burrows–Wheeler alignment tool^69^. Local realignment and quality score recalibration were conducted using the Genome Analysis Toolkit (GATK) according to GATK best practices^70^. Somatic alterations were identified (single-nucleotide variants, small insertions/deletions (indels) and copy number alterations). Single-nucleotide variants were identified using UnifiedGenotyper and mu Tect^71^. All samples were paired (AML/healthy) and candidate genomic alteration were reviewed manually in the Integrative Genomics Viewer^72^.

### RNA sequencing

RNA was isolated from a maximum of 0.5 × 10^6^ cells using the QIAGEN RNeasy Micro kit according to the manufacturer’s instructions. Total RNA (100 μg) was used for next-generation sequencing and prepared according to the NEBNext Ultra II RNA Library Prep kit for Illumina (New England Biolabs, NEB; cat. E7770S). The Supplementary Note contains further detailed descriptions of the method for library preparation. Libraries were sequenced using a high-output, single-end, 75 cycle (v.2) sequencing kit on the Illumina NextSeq 550 platform. Reads were trimmed for adaptor sequences using Cutadapt (v.1.11) and aligned using Spliced Transcripts Alignment to a Reference (STAR) (v.2.5.2a)^73^ to the GrCH37 assembly using the gene, transcript and exon features of Ensembl (release 70) gene model. Expression was estimated using RNA-seq by Expectation Maximization (RSEM) (v.1.2.30). Transcripts with zero read counts across all samples were removed before analysis. Normalization of read counts was performed by dividing by 1 million reads mapped to generate counts per million, followed by the trimmed mean of M-values method from the edgeR package (v.3.14.0)^74^. For the differential expression analysis, reads were filtered but not normalized, as edgeR performs normalization (library size and RNA composition) internally. For the differential expression (DE) analyses, the glmFit function was used to fit a negative binomial generalized log-linear model to the read counts for each transcript. Using the glmLRT function, we conducted transcript-wise likelihood ratio tests for each genotype comparison. Principal-component analysis was also performed on all DE transcripts with FDR < 0.05. GSEA of transcriptomics data was performed using GSEA (v.4.1.0) from the Broad Institute^75^. *P* values were generated from 1,000 gene set permutations, excluding gene sets with more than 3,000 genes or fewer than five genes against custom made gene sets and the Broad Institute’s Hallmark database.

### Lipidomics

Targeted lipidomics was performed on a 1290 Infinity II UHPLC coupled to a 6470 QQQ mass spectrometer via AJS ESI source (Agilent) in positive ionization mode, using a scheduled multiple reaction monitoring (MRM) method^76^. The MRM transition list contained 20 lipid classes and 593 lipid species (excluding internal standards CUDA and SPLASH Lipidomix). Skyline-daily software was used for lipid species assignment^77^. A lipid set enrichment analysis was performed by ranking fold changes, calculating enrichment scores and estimating the significance of enrichment using a permutation algorithm^78^. The Supplementary Note contains detailed descriptions of the procedure.

### Statistical analyses

Unless otherwise stated, statistical analyses were carried out using GraphPad Prism v.9.4.0. Microsoft Excel for Mac v.16.75.2 was used to re-calculate those *P* values < 0.0001 obtained from GraphPad Prism with higher precision. JMP Pro v.17 was used to calculate statistics related to the AML PDX trials, including PB donor chimerism AUC values and testing of normal distributions.

### Statistics and reproducibility

Study design was based on sample sizes that proved to be adequate in previous experiments using similar approaches and thus no statistical methods were used to predetermine sample sizes for this study^4,10^. The PDX trials were subject to randomization after transplantation by independent technicians without the involvement of the researcher who had performed the transplantations. The investigators were not blinded to allocation during experiments and outcome assessments; however, unbiased PDX monitoring and scoring were performed by independent technicians not intellectually involved in the study. For cell culture experiments, samples were allocated equally to ensure that covariates were identical between the compared groups. The investigators performing cell culture experiments were not blinded during allocation and outcome assessment. Data distribution was assumed to be normal, but this was not formally tested in each experiment.

### Reporting summary

Further information on research design is available in the Nature Portfolio Reporting Summary linked to this article.

### Supplementary information

Supplementary Figs Supplementary Figs. 1–4.

Reporting Summary

Supplementary Note Supplementary protocols.

Supplementary Tables Supplementary Tables 1–10

### Source data

Source Data Fig. 1 Statistical Source Data.

Source Data Fig. 2 Statistical Source Data.

Source Data Fig. 3 Statistical Source Data.

Source Data Fig. 4 Statistical Source Data.

Source Data Fig. 5 Statistical Source Data.

Source Data Fig. 6 Statistical Source Data.

Source Data Fig. 7 Statistical Source Data.

Source Data Fig. 8 Statistical Source Data.

Source Data Extended Data Fig. 1 Statistical Source Data.

Source Data Extended Data Fig. 2 Statistical Source Data.

Source Data Extended Data Fig. 3 Statistical Source Data.

Source Data Extended Data Fig. 4 Statistical Source Data.

Source Data Extended Data Fig. 4 Uncropped blots.

Source Data Extended Data Fig. 5 Statistical Source Data.

Source Data Extended Data Fig. 6 Statistical Source Data.

Source Data Extended Data Fig. 7 Statistical Source Data.

Source Data Extended Data Fig. 8 Statistical Source Data.

Source Data Extended Data Fig. 9 Statistical Source Data.

Source Data Extended Data Fig. 10 Statistical Source Data.

## Data Availability

RNA-sequencing data have been deposited in the Gene Expression Omnibus under accession codes GSE176522 and GSE176523. Targeted lipidomics data have been deposited in Panorama Public under a permanent link (https://panoramaweb.org/ImetelstatLipidomics.url). The following publicly available datasets generated by others have been used in this study: genome assembly GRCh37 in GenBank under accession code GCA_000001405.1 and COSMIC database v.80 (https://cancer.sanger.ac.uk/cosmic). Source data for Figs. 1–8 and Extended Data Figs. 1–10 have been provided as Source Data files. All other data supporting the findings of this study are available from the corresponding authors on reasonable request. Source data are provided with this paper.
