# Supplementary material for: Imetelstat-mediated alterations in fatty acid metabolism to induce ferroptosis as a therapeutic strategy for acute myeloid leukemia
Source: Nat Cancer. 2023 Oct 30;5(1):47–65. doi: 10.1038/s43018-023-00653-5 (PMC10824665; doi:10.1038/s43018-023-00653-5)
Supplement: Supplementary file 1 — Supplementary Figs. 1–4. [file 43018_2023_653_MOESM1_ESM.pdf]

nature cancer

Article

https://doi.org/10.1038/s43018-023-00653-5

Imetelstat-mediated alterations in fatty acid metabolism to induce ferroptosis as a therapeutic strategy for acute myeloid leukemia

In the format provided by the authors and unedited

SUPPLEMENTARY FIGURES

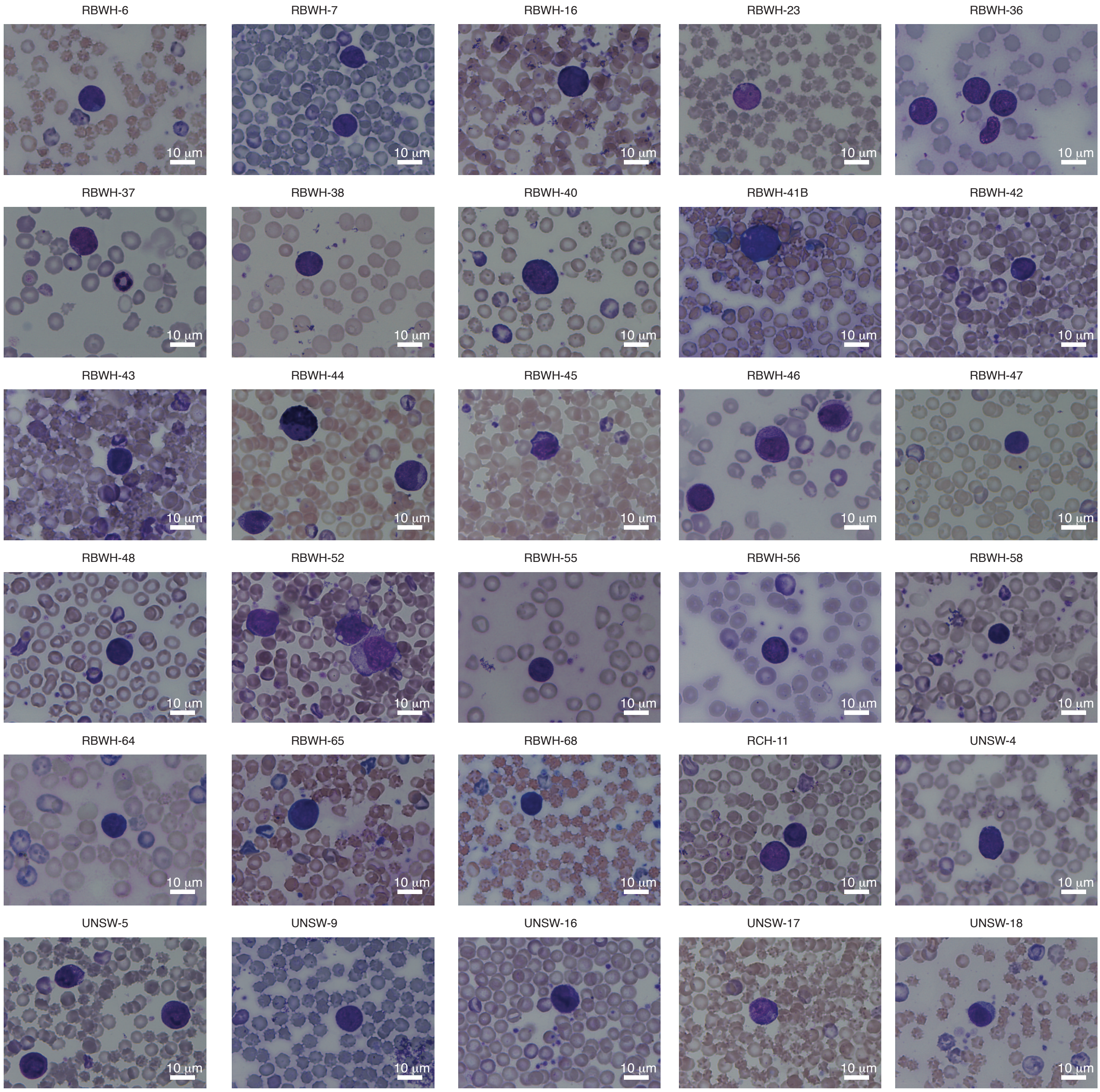

Supplementary Fig.1 | Peripheral blood blast morphology in successfully established AML PDX. Wright-Giemsa stainings were performed on peripheral blood smears from each of n = 30 individual AML PDX models.

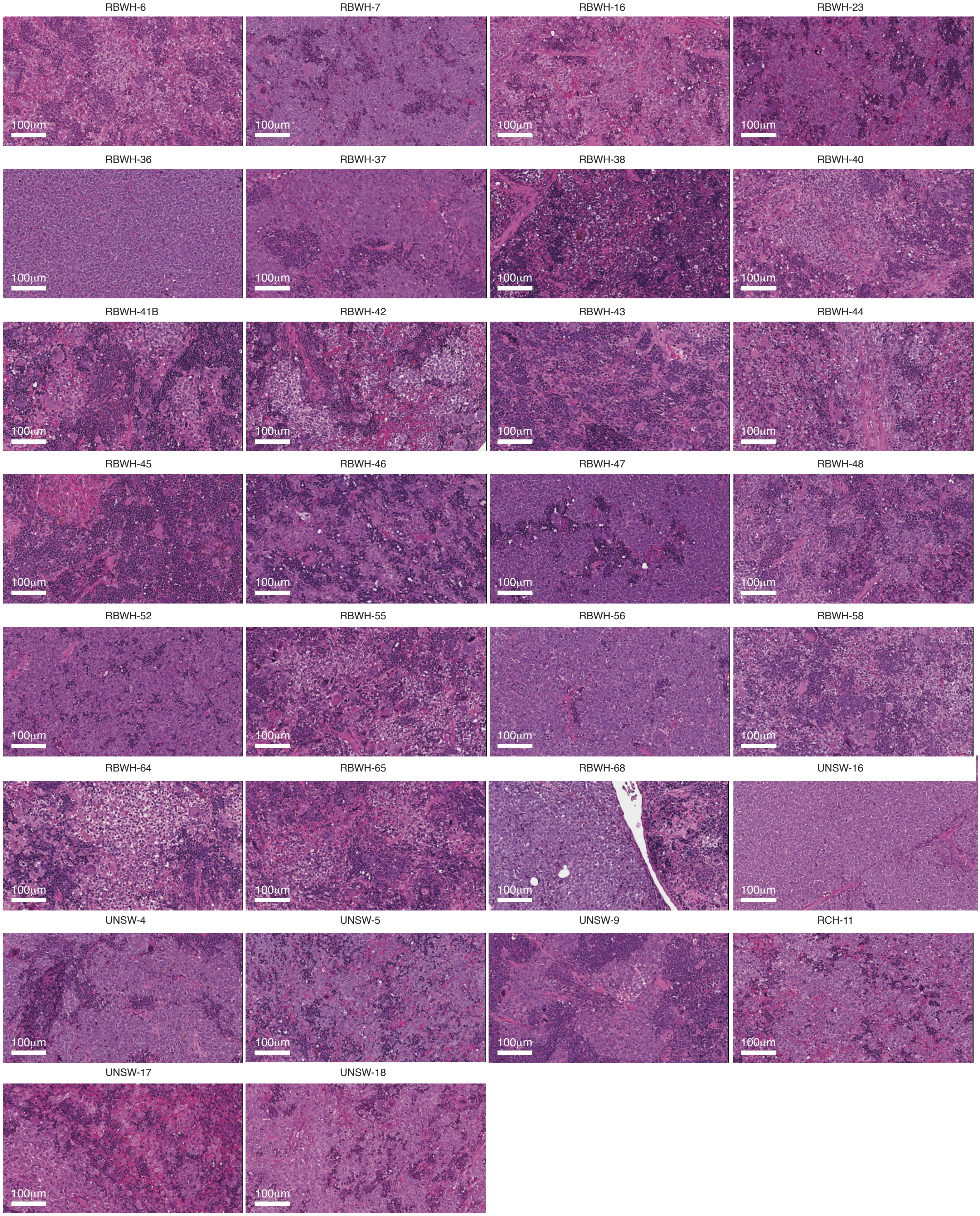

Supplementary Fig.2 | Splenic AML infiltration in successfully generated PDX.
Hematoxylin & Eosin stainings were performed on splenic sections from each of n = 30 individual AML PDX models.

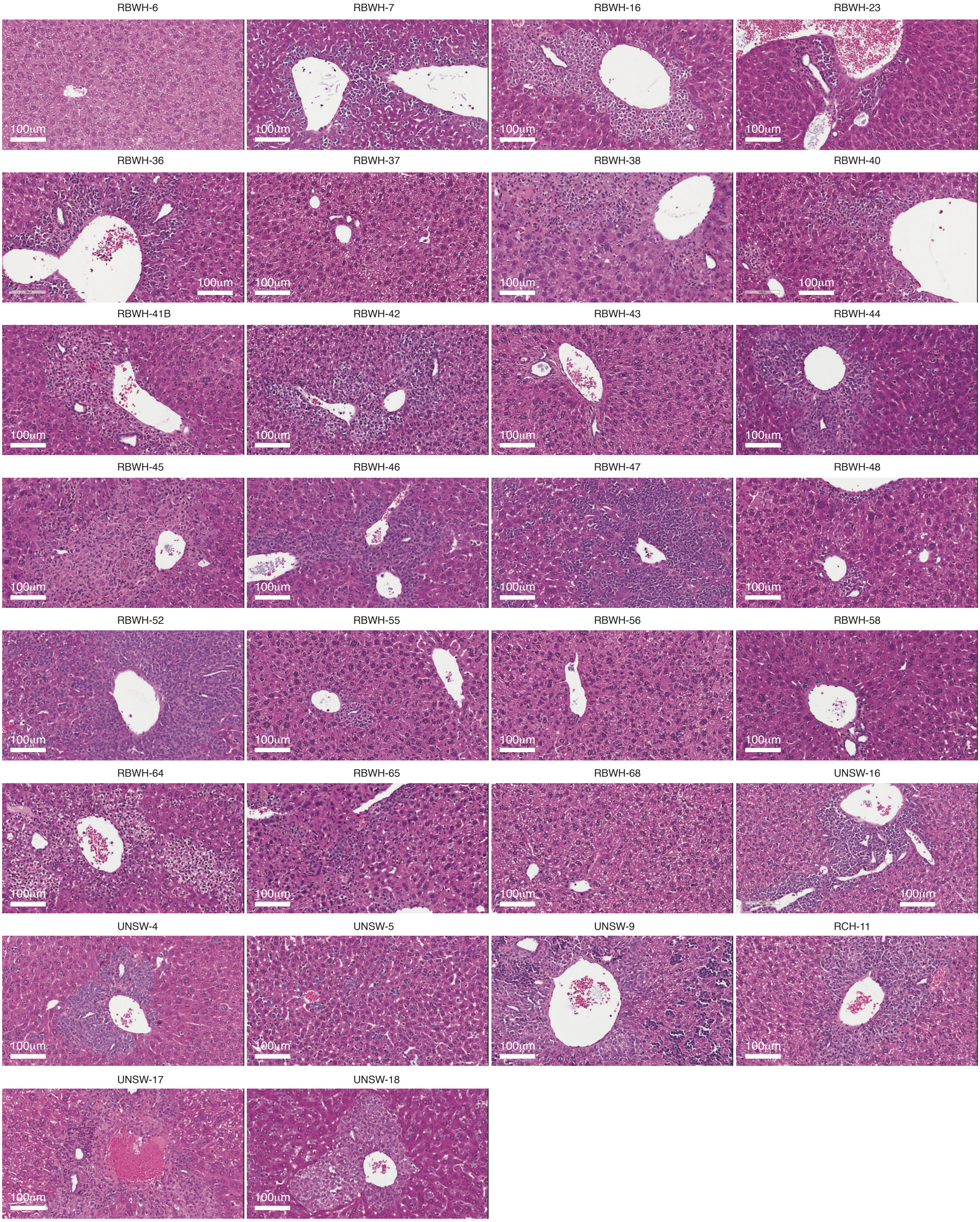

Supplementary Fig.3 | Hepatic AML infiltration in successfully generated PDX.
Hematoxylin & Eosin stainings were performed on liver sections from each of n = 30 individual AML PDX models.

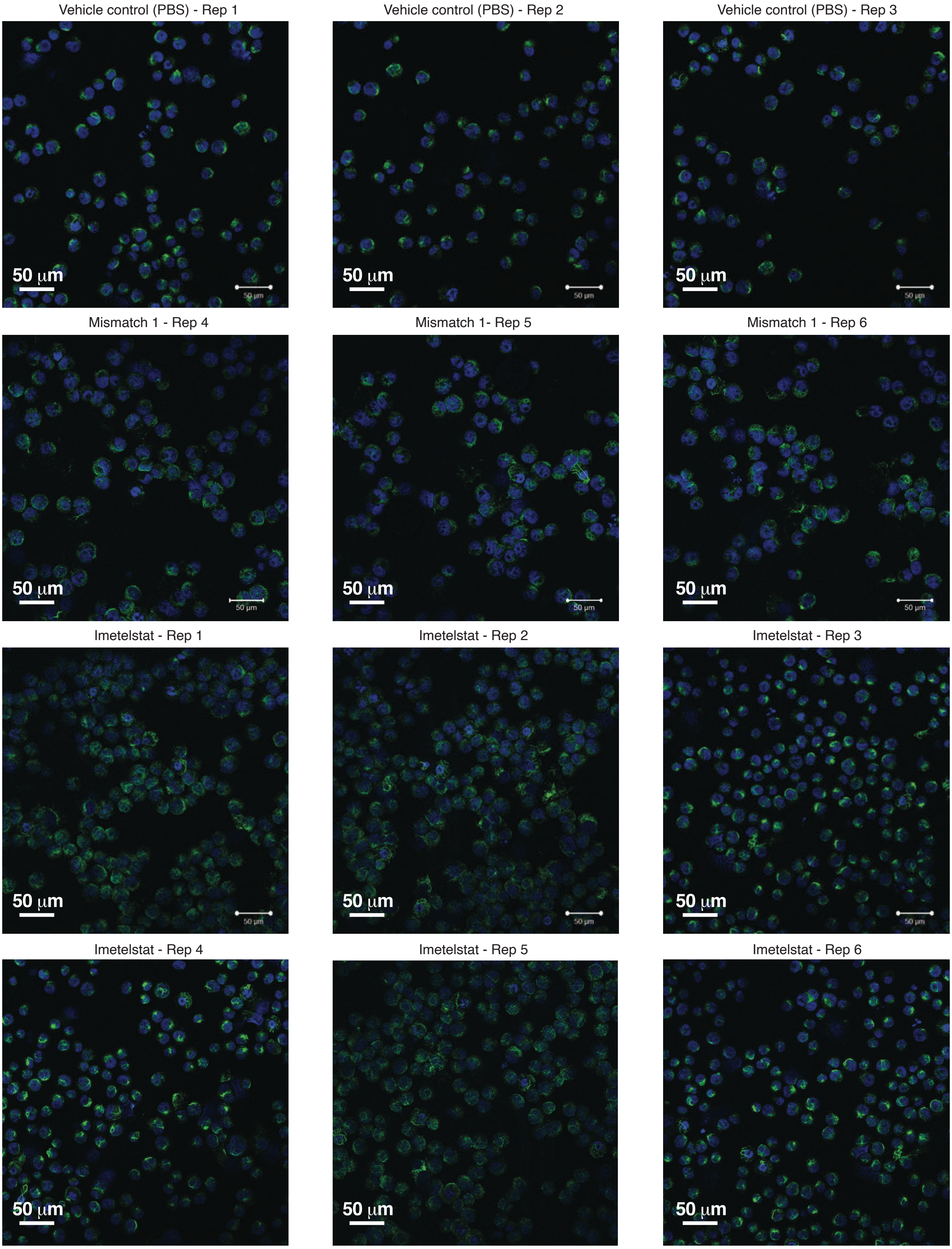

Supplementary Fig.4 | Vimentin expression in AML cell lines.
Confocal microscopy on vimentin in NB4 cells treated with vehicle control (PBS) or imetelstat (4 microM) for 24 hours. N = 6 independent biological replicates per condition.
