# Supplementary material for: Imetelstat-mediated alterations in fatty acid metabolism to induce ferroptosis as a therapeutic strategy for acute myeloid leukemia
Source: Nat Cancer. 2023 Oct 30;5(1):47–65. doi: 10.1038/s43018-023-00653-5 (PMC10824665; doi:10.1038/s43018-023-00653-5)
Supplement: Supplementary file 2 — Reporting Summary [file 43018_2023_653_MOESM2_ESM.pdf]

nature portfolio

Corresponding author(s): Claudia Bruedigam, Steven W. Lane

Last updated by author(s): Jul 22, 2023

Reporting Summary

Nature Portfolio wishes to improve the reproducibility of the work that we publish. This form provides structure for consistency and transparency in reporting. For further information on Nature Portfolio policies, see our Editorial Policies and the Editorial Policy Checklist.

Statistics

For all statistical analyses, confirm that the following items are present in the figure legend, table legend, main text, or Methods section.

n/a	Confirmed	
☐	☒	The exact sample size (*n*) for each experimental group/condition, given as a discrete number and unit of measurement
☐	☒	A statement on whether measurements were taken from distinct samples or whether the same sample was measured repeatedly
☐	☒	The statistical test(s) used AND whether they are one- or two-sided<br>*Only common tests should be described solely by name; describe more complex techniques in the Methods section.*
☒	☐	A description of all covariates tested
☐	☒	A description of any assumptions or corrections, such as tests of normality and adjustment for multiple comparisons
☐	☒	A full description of the statistical parameters including central tendency (e.g. means) or other basic estimates (e.g. regression coefficient) AND variation (e.g. standard deviation) or associated estimates of uncertainty (e.g. confidence intervals)
☐	☒	For null hypothesis testing, the test statistic (e.g. *F*, *t*, *r*) with confidence intervals, effect sizes, degrees of freedom and *P* value noted<br>*Give P values as exact values whenever suitable.*
☒	☐	For Bayesian analysis, information on the choice of priors and Markov chain Monte Carlo settings
☒	☐	For hierarchical and complex designs, identification of the appropriate level for tests and full reporting of outcomes
☒	☐	Estimates of effect sizes (e.g. Cohen's *d*, Pearson's *r*), indicating how they were calculated

Our web collection on statistics for biologists contains articles on many of the points above.

Software and code

Policy information about availability of computer code

Data collection

Blood analysis:
Peripheral blood samples were analyzed on a Hemavet 950 analyzer (Drew Scientific).

Histology:
Images of histological slides were obtained on a ScanScope FL (Aperio).

In vitro cell growth analysis:
Cells were analyzed with Celltiter 96 aqueous nonradioactive cell proliferation assay (MTS Systems) according to the manufacturer's instructions (Promega). Endpoint absorbance at 490 nm was detected using Biotek PowerWave.

Flow cytometry:
Data were acquired on a FACS LSR Fortessa (BD Biosciences).

Imaging flow cytometry:
Data were acquired using an Amnis® ImageStream®X Mark II Imaging Flow system.

Confocal microscopy:
Images were acquired on a Zeiss 780-NLO confocal microscope.

Terminal Restriction Fragment (TRF) analysis:
Telomeric signals were visualized by PhosphorImage analysis (Conomos et al., 2012).

Telomere length Q-PCR:
Telomere length was assessed by Q-PCR using an Applied Biosystem ViiA7 thermocycler.

Western Blotting:
Protein was detected using Immobilon chemiluminescent HRP substrate (WBKLS0500, Millipore) and imaged with the iBright CL1500 imaging system.

Brunello gRNA sequencing:
Samples were sequenced using the NextSeq 550 Illumina platform.

RNAseq analysis:
Libraries were sequenced using a High output, single-end, 75 cycle (version 2) sequencing kit on the Illumina Nextseq 550 platform.

Mutational sequencing:
Genomic alterations were profiled using the HemePACT assay (Integrated Mutation Profiling of Actionable Cancer Targets related to Hematological malignancies) at the Genomics Core Laboratory at MSKCC using the Illumina Hiseq 2500 platform.

Lipidomics:
Data were obtained from an LC/MS platform consisting of a 1290 Infinity II UHPLC coupled to a 6470 QQQ mass spectrometer via AJS ESI source (Agilent, Santa Clara, USA).

Data analysis

For all data analyses, either commercial or previously published open source packages were used: Rstudio version 1.0.143; Graphpad Prism version 9.4.0; Microsoft Excel version 16.75.2; Cytoscape version 3.5.1; GSEA version 4.0.3; IPA (Ingenuity pathway analysis software) version 01-19-00; JMP Pro, version 17.

Flow cytometry analysis:
Post-acquisition analyses were performed with FlowJo software V10.9.0 (Becton Dickinson & Company; BD).

Imaging flow cytometry:
Data were analyzed using IDEAS (Image Data Exploration and Analysis Software).

Terminal Restriction Fragment (TRF) analysis:
TRFs were processed by ImageJ 1.52a analysis software to quantitate mean telomere length.

Western blotting:
Protein expression was quantified by densitometric analysis using ImageJ 1.52a software.

In vitro cell growth and drug synergy analysis:
Absorbance data derived from celltiter assays were analyzed using Gen5 software. Synergism was estimated using the Synergyfinder 2.0 algorithm (http://synergyfinder.fimm.fi).

Brunello gRNA sequencing:
The sequenced gRNA insert from each read was mapped to a reference file of each gRNA in the library. The STARS and RIGER CRISPR screen analysis tools were applied to the sequencing results (Doench JG, Fusi N, et al. Nature Biotechnology. 2016;34(2):184-191) with analysis based on log2-transformed numbers of gRNA reads in 'End' samples after drug treatment compared to 'Input' samples.

RNAseq analysis:
Reads were trimmed for adapter sequences using Cutadapt (version 1.11) and aligned using Spliced Transcripts Alignment to a Reference (STAR) (version 2.5.2a; Dobin A, Davis CA, et al. Bioinformatics. 2013;29(1):15-21) to the GrCH37 assembly using the gene, transcript, and exon features of Ensembl (release 70) gene model. Expression was estimated using RNA-Seq by Expectation Maximization (RSEM) (version 1.2.30). Transcripts with zero read counts across all samples were removed prior to analysis. Normalization of read counts was performed by dividing by 1 million reads mapped to generate counts per million (CPM), followed by the trimmed mean of M-values (TMM) method from the edgeR package (version 3.14.0; Robinson MD, McCarthy DJ, et al. Bioinformatics. 2010;26(1):139-140).
For the differential expression (DE) analyses, the glmFit function was used to fit a negative binomial generalised log-linear model to the read counts for each transcript. Using the glmLRT function, we conducted transcriptwise likelihood ratio tests for each genotype comparison. Principal component analysis (PCA) was also performed on all DE transcripts with FDR <.05. Gene set enrichment analysis (GSEA) of transcriptomics data was performed using GSEA (version 4.1.0) from the Broad Institute (Subramanian A, Tamayo P, et al. Proceedings of the National Academy of Sciences. 2005;102(43):15545-15550). P-values were generated from 1000 gene set permutations, excluding gene sets with more than 3000 genes or less than 5 genes against custom made gene sets and the Broad Institute's Hallmark database. GSEA results were visualized using Cytoscape version 3.5.1 software using a node-cutoff of q<0.1.

Mutational sequencing:
Reads were aligned to the reference human genome (hg19) using the Burrows-Wheeler Alignment tool (Li H, Durbin R. Fast and accurate short read alignment with Burrows-Wheeler transform. Bioinformatics 2009;25:1754–60). Local realignment and quality score recalibration were conducted using the Genome Analysis Toolkit (GATK) according to GATK best practices (Braunschweig AB, Huo F, Mirkin CA. Molecular printing. Nat Chem 2009;1:353–8). Somatic alterations were identified (single-nucleotide variants, small insertions/deletions (indels), and copy number alterations). Single-nucleotide variants were identified using UnifiedGenotyper and mu Tect (Cibulskis, K. et al. Sensitive detection of somatic point mutations in impure and heterogeneous cancer samples. Nat Biotechnology (2013).doi:10.1038/nbt.2514). All samples were paired (Tumor/Normal) and candidate genomique alteration were reviewed manually in the Integrative Genomics Viewer (Robinson JT, Thorvaldsdottir H, Winckler W, Guttman M, Lander ES, Getz G, et al. Integrative genomics viewer. Nat Biotechnol 2011;29:24–6). Identified mutations were filtered based on the COSMIC database (Tate JG, Bamford S, Jubb HC, Sondka Z, Beare DM, Bindal N, et al. COSMIC: the Catalogue Of Somatic Mutations In Cancer. Nucleic Acids Res 2019;47(D1):D941-D7 doi 10.1093/nar/gky1015). The frequencies of genes with oncogenic mutations were visualized using cytoscape in the distinct imetelstat response groups. Co-occurring mutations within the same individual AML patient sample were highlighted by connecting lines.

Lipidomics:
Skyline-daily software (Adams et al., 2020) was used for lipid species assignment (precursor/product ion pairs and retention time) and chromatographic peak integration based on Huynh et al., 2019. An indexed retention time (iRT) calculator, which was generated using internal standards as well as lipid species assigned from the sample pool run, was employed for a retention time predictor to increase confidence of lipid assignment in subsequent samples. Peak picking was manually inspected using Skyline's retention times – replicate comparison pane and adjusted accordingly by comparing retention time and chromatographic peak profile to the sample pool QC run. Furthermore, all peaks were manually checked for correct integration. The downstream data processing and visualization was carried out with R package lipidr (version 2.14.1) (Mohamed et al., 2020). Skyline transition results and a file with sample annotation and grouping were imported into lipidr (version 2.14.1) . Raw data quality was assessed by plotting total lipid intensities of each sample as well as intensity and retention time distributions of internal standards across samples. Log2 transformation and probabilistic quotient normalization (PQN) was performed prior to statistical analysis. Data analysis was carried out using the R package lipidr. Sample variation was investigated by principal component analysis (PCA). A lipid set enrichment analysis was performed by plotting results for enriched lipid classes and lipid unsaturation either as boxplot or trend line. All p-values were adjusted for multiple comparisons.

For manuscripts utilizing custom algorithms or software that are central to the research but not yet described in published literature, software must be made available to editors and reviewers. We strongly encourage code deposition in a community repository (e.g. GitHub). See the Nature Portfolio guidelines for submitting code & software for further information.

Data

Policy information about availability of data

All manuscripts must include a data availability statement. This statement should provide the following information, where applicable:

- Accession codes, unique identifiers, or web links for publicly available datasets
- A description of any restrictions on data availability
- For clinical datasets or third party data, please ensure that the statement adheres to our policy

RNA–sequencing data have been deposited in the Gene Expression Omnibus (GEO) under accession codes GSE176522 and GSE176523.

Targeted lipidomics data have been deposited in Panorama Public under a permanent link (https://panoramaweb.org/ImetelstatLipidomics.url).

The following publicly available datasets generated by others have been used in this study:
Genome assembly GRCh37 in GenBank under accession code GCA_000001405.1, and COSMIC database version 80 (https://cancer.sanger.ac.uk/cosmic).

Source data for Fig. 1-8 and Extended Data Fig. 1-10 have been provided as Source Data files. All other data supporting the findings of this study are available from the corresponding authors on reasonable request.

Human research participants

Policy information about studies involving human research participants and Sex and Gender in Research.

Reporting on sex and gender	Bone marrow samples were collected from both female and male patients with Acute Myeloid Leukaemia. PDX for this study were generated from samples of 13 male, and 17 female patients. Gender/sex is listed in the patient characteristics and is representative of a standard AML cohort. Gender was assigned based on genetic analysis of the tumor and was concordant with self-reported gender in all cases.
Population characteristics	Patient ages ranged between 18 and 88. In addition, one pediatric AML patient sample was included in the PDX trial.
Recruitment	Consecutive patients with AML who presented via the Royal Brisbane and Women's Hospital and had bone marrow aspirate were consented for research sample collection and baseline clinical data according to p1382 human research ethics protocol (QIMR Berghofer HREC/14/QRBW/278). Written informed consent was obtained at the time of bone marrow aspiration from consenting patients. There was no predetermined bias, however samples were collected during standard working hours to facilitate optimal processing and viable cell storage.
Ethics oversight	Primary AML samples were obtained from patients with AML, after informed consent in accordance with the Declaration of Helsinki, and approved by the institutional (QIMR Berghofer) ethics committee protocol P1382 (HREC/14/QRBW/278).

Note that full information on the approval of the study protocol must also be provided in the manuscript.

Field-specific reporting

Please select the one below that is the best fit for your research. If you are not sure, read the appropriate sections before making your selection.

☒ Life sciences ☐ Behavioural & social sciences ☐ Ecological, evolutionary & environmental sciences

For a reference copy of the document with all sections, see nature.com/documents/nr-reporting-summary-flat.pdf

Life sciences study design

All studies must disclose on these points even when the disclosure is negative.

Sample size	Study design was based on sample sizes that proved to be adequate in previous experiments using similar approaches, and thus no statistical methods were used to pre-determine sample sizes for this study (Bruedigam et al., Cell Stem Cell 2014; Townsend et al., Cancer Cell 2016).
Data exclusions	We needed to prematurely terminate and subsequently repeat 6 PDX experiments from the imetelstat AML NSGS trial due to infection of the mice with alpha hemolytic streptococcus viridans. In some cases, PDX needed to be culled immediately without possible subsequent analysis of AML burden in bone marrow and peripheral organs. GPR56 flow cytometry was included in the PDX analysis at a later stage following its first publication as leukaemic stem cell marker. For the cell culture data presented in Fig. 5a, one out of three technical replicates from each control condition were excluded as cellrox green reagent was erroneously not added to the respective wells.<br>No other data were excluded from the analyses.

Replication

The Phase II-like randomized trial of imetelstat in AML NSGS PDX was initially performed and analyzed as two separate cohorts with n = 15 individual AML patient samples each (cohort 1: 2013-2017; cohort 2: 2016-2018). Information in cohort allocation is provided in Extended Data Fig. 9b, demonstrating equal distribution over all imetelstat response groups. Both cohorts demonstrated significant improvement in survival of imetelstat-treated PDX compared to vehicle control-treated PDX (survival cohort 1: 103 days (vehicle) and 152 days (imetelstat) with $p < 0.0001$; survival cohort 2: 110 days (vehicle) and 141 days (imetelstat) with $p = 0.0131$). PDX from these cohorts were treated, monitored and analyzed by independent research technicians. Moreover, the imetelstat monotherapy trial was also included in the combination treatment NRGS cohort (20 AML patient samples) and thus partially, independently replicated in an additional recipient strain.

Most cell culture experiments were replicated by independent researchers within the laboratory. Cell culture experiments were repeated at least once independently with cell lines from a different passage with similar results. We would like to point out the observed imetelstat inoculum effect (Extended Data Fig. 4a). All cell culture experiments included in this study were performed on low-density pre-cultures, passaged between 12-24h prior seeding at a density of ~1x10^5 cells per ml, with a maximum density of 5x10^5 cells per ml when cells were taken for experimental setup. We found this procedure to be absolutely essential for the reproducibility of the results presented in this study.

Detailed information on replication for each Figure:

Fig.1a-c: demonstrating descriptive baseline data of the AML patient sample resource. The RNAseq analysis (a) was performed simultaneously on all samples; HemePACT analysis was done on the samples split into two cohorts as described above. Cross-checking was carried out successfully between oncogenic mutations in highly expressed genes identified in HemePACT, RNAseq and clinical data where available.

Fig.2a-i: demonstrating data from the two combined preclinical trials on imetelstat monotherapy in AML PDX that were initially analyzed separately as described above, with similar results in each cohort. The graphs show data from n = 180 PDX per treatment group, with 6 PDX generated from each 30 individual AML patient samples per group.

Fig. 2j-i: demonstrating data from RNAseq analyses on n = 8 AML PDX models with n = 2 PDX per AML patient sample, and n = 2 PDX per treatment group. GSEA was performed on imetelstat vs. vehicle treated groups in which PDX from different paired AML patient samples were pooled (n = 16 PDX per treatment group). GSEA were also carried out between the treatment groups within individual AML PDX models with similar results.

Fig. 2m,n: demonstrating data from telomere length analyses using qPCR (m) in the same samples used as in 2j-i. Each dot shows the mean of two technical replicates. Data were calculated based on two distinct housekeeping genes. One sample was randomly chosen and verified using TRF.

Fig. 3a-b: demonstrating data from the Brunello CRISPR screen in which n = 3 replicates per each condition were passaged strictly separately over a timecourse of 45 days, resulting in cultures with specific combinations of enrichments of gRNAs from overlapping biochemical pathways, and distinct growth kinetics as demonstrated in Extended Data Fig. 4b,c, thus generating de-facto biological replicates. The individual hits from this CRISPR screen were validated in separate experiments using single gRNAs in four cell lines (Fig. 3c-d).

Fig. 3c-f: demonstrating data from competition assays of imetelstat versus vehicle control treated Cas9-expressing NB4, MV411, KO52, and TF1 cultures transduced with n = 2 independent single guide RNAs targeting FADS2 (top panel), n = 4 independent single gRNAs targeting ACSL4 (middle panel), and n = 2 controls (empty vector and gRNA targeting CD33). Three technical replicates per condition from two independent experiments were pooled. Each experiment analyzed separately revealed similar results.

Fig. 4a: Lipidomics analysis on NB4 cells with n = 3 biological replicates from distinct cell passages and independent experiments.

Fig. 4b-c: demonstrating data from CellROX-Green and C11-BODIPY analyses in ACSL4-edited (n = 4 independent gRNAs), FADS2-edited (n = 2 independent gRNAs) or non-edited (n = 2 independent replicates, i.e. Cas9, empty vector) NB4 or MV411 cell lines treated with imetelstat or PBS. Three technical replicates per condition were pooled. Data are presented as mean ± SEM. A repeat experiment was performed that replicated the results.

Fig. 5a,b: demonstrating data from CellROX-Green and C11-BODIPY flow-cytometry on NB4, MV411, KO52 and TF1 treated with imetelstat or vehicle-control with n = 6 replicates pooled from two experiments (with 3 technical replicates per condition). Similar results were obtained when both experiments were analyzed separately.

Fig. 5c: demonstrating data from C11-BODIPY and TERT-mRNA analyses on sorted viable CD45+ splenic cells from imetelstat- compared to PBS-treated PDX from the preclinical trial presented in Figure 2. C11-BODIPY data (n = 9 PDX from three individual AML samples with three PDX per patient sample) are presented. ACSL4 mRNA data (n = 6 PDX from the same three individual AML samples with two PDX per patient sample) are presented.

Fig. 5d-f: demonstrating data from AML PDX treated with vehicle, liproxstatin-1, imetelstat, or the combination of liproxstatin-1 with imetelstat with n = 12 PDX per treatment group, with six PDX each from two independent AML patient samples (and independent experiments) pooled. Separate analysis of the two experiments revealed similar results.

Fig. 6b: showing representative images from confocal microscopy of VIM protein in NB4 cells with n = 6 independent biological replicates per condition. Images from all replicates are provided as SourceImage file in the Supplemental Information document.

Fig. 6c: demonstrating data on VIM-editing in NB4 using n=4 independent single gRNAs, and thus four independent isogenic cell lines. Plots show data from one representative experiment. Two independent repeats were performed with similar results.

Fig. 6d: showing representative data from imaging flow cytometry of lipophagy in n = 4 independent VIM-edited (VIM-sg1, VIM-sg2, VIM-sg3, and VIM-sg4) or n = 4 independent editing-control (i.e. native, Cas9, empty vector, CD33-sg2) NB4 cell-lines. This experiment was repeated three times with similar results.

Fig. 6e: demonstrating celltiter-based cell growth data in 6 individual AML cell-lines: NB4 (n=3 technical replicates), MV411 (n=3 technical replicates), KO52 (n=3 technical replicates), TF1 (n=2 technical replicates): P=5.1×10-3; MOLM13 (n=3 technical replicates): P<1×10-4; and HEL (n=3 technical replicates). Each experiment was repeated once with similar results.

Fig. 7a: showing data from HemePACT analysis of AML patient samples at baseline (data presented in Fig. 1c), but here segregated into distinct imetelstat response groups with n = 14 sustained, n = 8 intermediate, and n = 8 poor responders to imetelstat. As described above, the identified mutations were cross-checked with data obtained from RNAseq and clinical analyses, and could be confirmed when VAF and read counts were sufficient.

Fig. 7b-c: showing data on AML burden and survival in imetelstat-treated normalized to vehicle control-treated PDX in relation to NRAS mutational status: NRAS wild-type (wt; n = 144 PDX from 24 individual AML patient samples with six PDX each), mutant NRAS (mut; n = 36 PDX from 6 individual AML patient samples with 6 PDX each). This is a descriptive analysis to be verified in future independent studies and clinical trials.

Fig. 7d-e: showing data from RNAseq experiments on AML patient samples baseline (data presented in Fig. 1a) segregated into distinct imetelstat response groups with n = 14 sustained, n = 8 intermediate, and n = 8 poor responders to imetelstat. GSEA was performed comparing RNAseq data from sustained versus poor responders to imetelstat. This is also a descriptive analysis to be verified in future independent work.

Fig. 7f: demonstrating simple linear regression analysis of baseline telomere length vs. imetelstat response in PDX with n = 30 AML patient samples in total. The imetelstat response scores were derived from PDX per individual AML patient sample.

Fig. 8a-b: demonstrating CellROX and sytox flow-cytometry on AML cells with n= 3 replicates from a representative experiment that was repeated independently showing similar results.

Fig. 8c-d: demonstrating data on C11-BODIPY and CellROX analysis on AML PDX (RBWH-44) treated with vehicle, Arac+Doxo, imetelstat, or Arac+Doxo followed by imetelstat. c, n = 5 PDX (vehicle), n = 6 (imetelstat), n = 6 PDX (Arac+Doxo) and n = 6 PDX (Arac+Doxo followed by imetelstat). d, n = 5 PDX (vehicle), n = 6 PDX (imetelstat), n = 5 PDX (Arac+Doxo), and n = 5 PDX (Arac+Doxo followed by imetelstat). The analyses were performed in separate batches on different days of approximately two PDX per treatment group. Separate analysis revealed similar trends for each analysis day.

Fig. 8f,g: showing data on the PDX trial on imetelstat consolidation following induction chemotherapy with n=120 PDX per treatment group, with 20 individual AML patient samples and 6 PDX per patient sample per group. The data from the imetelstat monotherapy arm were compared to the imetelstat monotherapy trial in NSGS, where individual AML patient samples showed similar imetelstat responses.

Extended Data Fig. 1a-p: presenting data on the initial AML PDX engraftment test experiments, transplanting 50 individual AML patient samples into approximately 6 NSGS recipients each. Histological analyses were carried out on AML PDX with confirmed AML donor chimerism from bone marrow, peripheral blood and spleen from n = 30 individual AML patient samples. A comprehensive collection of spleen and liver H&E as well as peripheral blood Wright-Giemsa stainings demonstrating blast morphology in all 30 cases is provided in SourceImage files in the Supplemental Information document. The purpose of data presentation in this figure is to validate the usefulness of our cut-offs used to define the successful generation of AML xenografts and onset of AML in these models.

Extended Data Fig. 2a-q: demonstrating data on humanized in vivo models of hematopoiesis generated by transplanting viable CD34+ mononuclear cells isolated from cord blood samples from two independent donors into NSG recipients (donor 1: n = 5 NSG per treatment group; donor 2: n = 6 NSG per treatment group). These experiments which show similar results were conducted independently over distinct time periods.

Extended Data Fig. 3a-d: showing data on the comparative analysis of AML PDX responses to imetelstat vs. standard induction chemotherapy. The datasets described in Fig. 1c (HemePACT data from AML patient samples at baseline) and preclinical combination trial data from Fig. 8e-g are shown here with the aim to focus on the differences between chemotherapy and imetelstat therapy efficacies, and potential mutational profiles that can predict preferential responses to each group. These are descriptive results to be validated in future work and clinical trials.

Extended Data Fig. 4a: demonstrating pooled IC50 results from n = 2 independent experiments. Both results revealed similar results when analyzed independently.

Extended Data Fig. 4b-g: demonstrating results from the Brunello screen. Please see details provided for Fig. 3a-b above in this reproducibility section.

Extended Data Fig. 4h,j: showing confirmation of single guide RNA mediated editing of ACSL4 by western blot (h) and FADS2 by TIDE analysis in n = 4 independent AML cell lines. Representative images are shown, please refer to SourceImage files in the Supplemental Information document for a compilation of images of all cell lines analyzed.

Extended Data Fig. 4i: showing intracellular flow cytometry on vimentin in editing-control or VIM-edited NB4 cells. Representative data from one experiment are shown. The analysis was repeated from cells of later passage and revealed similar results.

Extended Data Fig. 5a-c: showing results on lipidomics analysis in NB4 cells. Please refer to details provided under Fig. 4a in this reproducibility section above.

Extended Data Fig. 6: demonstrating data from celltiter-based cell growth analyses in a panel of n = 7 independent imetelstat - sensitive AML cell lines. The data were pooled from n = 2 independent experiments to plot BLISS and LOEWE synergy scores. For each hit, antagonistic scores were also obtained when the two independent experiments per drug and cell line were analyzed separately.

Extended Data Fig. 7a: showing celltiter-based growth analyses data on n = 14 human hematopoietic cell lines, of which data were pooled from three technical replicates per condition from n=2 independent experiments. Similar results were obtained when each experiment was analyzed separately.

Extended Data Fig. 7b: demonstrating data on DNA G-quadruplex flow cytometry in n=5 isogenic NB4 cell lines (native, Cas9, empty vector, CD33-sg1, CD33-sg2) per drug treatment group. Data are shown from a representative experiment from three independent experiments in total that revealed similar results.

Extended Data Fig. 7c: showing data from CellROX measurements of n=5 independent editing controls or n=2 independent FADS2-edited NB4 cell lines per treatment group. Each dot represents the mean of three technical replicates per cell line from one representative out of four independent experiments. Each repeat experiment showed similar results.

Extended Data Fig. 7d: demonstrating data on celltiter-based growth analysis with n = 3 technical replicates per condition from each one representative out of each two independent experiments in total. The repeat experiments revealed similar results.

Extended Data Fig. 7e: showing data on peripheral blood AML burden in AML PDX (RCH11) treated with PBS (n=6 PDX), MM1, (n=5 PDX), MM2 (n=5 PDX), or imetelstat (n=6 PDX). Due to limited availability of this particular mismatch control of interest (MM2), this experiment was performed only once but with multiple PDX / biological replicates in each group.

Extended Data Fig. 8: demonstrating peripheral blood donor chimerism timecourse data from the imetelstat monotherapy trial in AML PDX of n = 30 individual AML patient samples with n = 6 PDX per AML patient sample and treatment group. Please see above for details on reproducibility of the imetelstat AML PDX trial (beginning of this section, and also Fig. 2).

Extended Data Fig. 9a-d: Please see details above on the reproducibility of the imetelstat AML PDX trial (beginning of this section, and also Fig. 2).

Extended Data Fig. 10a-j: Please see details above on the reproducibility of the imetelstat AML PDX trial (beginning of this section, and also Fig. 2).

Randomization

Patient samples were allocated into the cohorts randomly, based on their availability and outcome of prior engraftment testing in NSGS recipients. PDX recipients were randomized into distinct treatment cages and ear-tagged by an independent research technician after transplantation without involvement of the researcher who had performed the transplantation.

For all other experiments, samples were allocated equally to ensure that covariates were identical between the compared groups.

Blinding

Blinding was not strictly imposed due to the high maintenance requirements of the PDX colonies requiring multiple researchers being able to collect and also analyze data, however, unbiased PDX monitoring and scoring was performed by independent animal technicians not intellectually involved in the study.

The investigators performing cell culture experiments and additional analyses were not blinded during allocation and outcome assessment, however, data collection and analyses were performed by independent researchers in the majority of cases.

Reporting for specific materials, systems and methods

We require information from authors about some types of materials, experimental systems and methods used in many studies. Here, indicate whether each material, system or method listed is relevant to your study. If you are not sure if a list item applies to your research, read the appropriate section before selecting a response.

Materials & experimental systems

n/a	Involved in the study	
☐	☒	Antibodies
☐	☒	Eukaryotic cell lines
☒	☐	Palaeontology and archaeology
☐	☒	Animals and other organisms
☒	☐	Clinical data
☒	☐	Dual use research of concern

Methods

n/a	Involved in the study	
☒	☐	ChIP-seq
☐	☒	Flow cytometry
☒	☐	MRI-based neuroimaging

Antibodies

Antibodies used

Antibodies used on western blots:
rabbit anti-ACSL4 antibody (abcam; cat. ab155282; clone EPR8640)
mouse anti-Cas9 (S. pyogenes) antibody (Cell Signaling; cat. #14697, clone 7A9-3A3)
mouse anti-Actin Ab-5 antibody (BD biosciences; cat. 612656; clone C4/actin (RUO))
polyclonal goat anti-rabbit immunoglobulins/HRP (Dako; cat. P0448)
polyclonal rabbit anti-mouse immunoglobulins/HRP (Dako; cat., P0260)

Antibodies used for confocal microscopy:
anti-human VIM (Vimentin (D21H3) XP® Rabbit mAb (Alexa Fluor® 488 Conjugate) (Cell Signaling; cat. #9854)
rabbit (DA1E) mAb IgG XP® Isotype Control (Alexa Fluor® 488 Conjugate) (Cell Signaling; cat. #2975)

Antibodies used in flow cytometry:
anti-human CD19 PerCP-Cy5.5 (Biolegend; cat. 302230 ; clone HIB19)
anti-human CD3 PE-Cy7 (Biolegend; cat. 344816; clone SK7)
anti-human CD33 APC (Biolegend; cat. 983902; clone WM53)
anti-human CD34 PE (Biolegend; cat. 343506; clone 581)
anti-human CD38 APC-Cy7 (Biolegend; cat. 303534; clone HIT2)
anti-human CD45 AF647 (Biolegend; cat. 304018 ; clone H130)
anti-human CD45 FITC (Biolegend; cat. 304006; clone H130)
anti-human GPR56 PE-Cy7 (Biolegend; cat. 358204; clone CG4)
anti-mouse Cd45.1 PE (Biolegend; cat. 110708; clone A20)
anti-mouse Cd45.1 PerCP-Cy5.5 (Biolegend; cat. 110728; clone A20)

For G-quadruplex analysis:
anti-DNA G-quadruplex (G4) (Merck Millipore; cat. MABE1126; clone 1H6)
anti-mouse-IgG2bκ FITC (Biolegend; cat. 402208; clone 27-35)

Antibody used for imaging flow cytometry:
anti-human CD107a (LAMP-1) AF647 (Biolegend; cat. 328612; clone H4A3)

Validation

Only validated antibodies were used with relevant supportive evidence and citations provided on the respective websites from Cell Signaling Technologies, Biolegend, BD Biosciences, Abcam and Merck Millipore:

rabbit anti-ACSL4 antibody (abcam; cat. ab155282; clone EPR8640):
raised against synthetic peptide within human ACSL4; validated by manufacturer using western blot analysis in HepG2, HeLa, HEK293 whole cell lysates, and human fetal kidney tissue lysate; immunohistochemistry on formalin/PFA-fixed paraffin-embedded sections of human hepatocellular carcinoma tissue comparatively stained with a secondary antibody only.

mouse anti-Cas9 (S. pyogenes) antibody (Cell Signaling; cat. #14697, clone 7A9-3A3):
raised against the amino terminus of Cas9 from Streptococcus pyogenes; validated by manufacturer using Western blot analysis of extracts from 293T cells, mock transfected (-) or transfected with a construct expressing Cas9 (+).

mouse anti-Actin Ab-5 antibody (BD biosciences; cat. 612656; clone C4/actin (RUO)):
raised against Chicken gizzard muscle Actin; verified by manufacturer using Western blot analysis on Jurkat cell lysate and immunofluorescent staining of Hs68 cells.

anti-human VIM (Vimentin (D21H3) XP® Rabbit mAb (Alexa Fluor® 488 Conjugate) (Cell Signaling; cat. #9854):
raised against synthetic peptide corresponding to residues surrounding Arg45 of human vimentin protein; validated by manufacturer using Flow cytometric analysis of MCF7 and HeLa cells comparatively stained with concentration-matched Rabbit (DA1E) mAb IgG XP® Isotype Control (Alexa Fluor® 488 Conjugate) #2975, as well as Confocal immunofluorescent analysis of SNB19 cells comparatively stained for Actin filaments using DY-554 phalloidin.

anti-human CD19 PerCP-Cy5.5 (Biolegend; cat. 302230; clone HIB19):
validated by manufacturer using flow cytometry on human peripheral blood lymphocytes comparatively stained with an isotype control.

anti-human CD3 PE-Cy7 (Biolegend; cat. 344816; clone SK7):
validated by manufacturer using flow cytometry on Human peripheral blood lymphocytes.

anti-human CD33 APC (Biolegend; cat. 983902; clone WM53):
raised against human myeloid leukaemia cells; validated by manufacturer using flow cytometry on human peripheral blood lymphocytes, monocytes, and granulocytes.

anti-human CD34 PE (Biolegend; cat. 343506; clone 581):
validated by manufacturer using flow cytometry on human peripheral blood mononuclear cells comparatively stained with PE mouse IgG1 isotype control and CD45 (HI30) PerCP (gated on CD14- cell population).

anti-human CD38 APC-Cy7 (Biolegend; cat. 303534; clone HIT2):
validated by manufacturer using flow cytometry on human peripheral blood lymphocytes comparatively stained with Mouse IgG1, APC/Cyanine7 isotype control.

anti-human CD45 AF647 (Biolegend; cat. 304018 ; clone H130):

validated by manufacturer using flow cytometry on human peripheral blood lymphocytes.

anti-human CD45 FITC (Biolegend; cat. 304006; clone H130):
validated by manufacturer using flow cytometry on human peripheral blood lymphocytes, monocytes and granulocytes.

anti-human GPR56 PE-Cy7 (Biolegend; cat. 358204; clone CG4):
raised against human GPR56-mouse Fc fusion protein; validated by manufacturer using flow cytometry on human peripheral blood lymphocytes comparatively stained with mouse IgG1, κ PE isotype control.

anti-mouse Cd45.1 PE (Biolegend; cat. 110708; clone A20):
raised against SJL mouse thymocytes and splenocytes; validated by manufacturer using flow cytometry on SJL and C57BL/6 splenocytes stained.

anti-mouse Cd45.1 PerCP-Cy5.5 (Biolegend; cat. 110728; clone A20):
raised against SJL mouse thymocytes and splenocytes; validated by manufacturer using flow cytometry on SJL mouse splenocytes.

anti-DNA G-quadruplex (G4) Antibody, clone 1H6 (Merck Millipore; cat. MABE1126):
raised against KLH-conjugated G-quadruplex DNA derived from oligonucleotides with telomeric repeats; validated by manufacturer using immunocytochemistry in HeLa cells.

anti-human CD107a (LAMP-1) AF647 (Biolegend; cat. 328612; clone H4A3):
raised against human adult adherent peripheral blood cells; validated by manufacturer using flow cytometry on thrombin-activated human peripheral blood platelets comparatively stained with mouse IgG1, κ Alexa Fluor® 647.

Eukaryotic cell lines

Policy information about cell lines and Sex and Gender in Research

Cell line source(s)

The following cell lines were purchased from DSMZ:
SKM1 (CVCL_0098; cat. ACC 547)
Monomac6 (CVCL_1426; cat. ACC 124)
MOLM13 (CVCL_2119; cat. ACC 554)
HEL (CVCL_0001; cat. ACC 11)
NB4 (CVCL_0005; cat. ACC 207)
PL21 (CVCL_2161; cat. ACC 536)
OCIAML3 (CVCL_1844; cat. ACC 582).

The following cell lines were purchased from ATCC:
K562 (CVCL_0004; cat. CCL-243)
HL60 (CVCL_0002; cat. CCL-240)
U937 (CVCL_0007; cat. CRL-1593.2)
MV411 (CVCL_0064; cat. CRL-9591)
THP1 (CVCL_0006; cat. TIB-202)
TF1 (CVCL_0559; cat. CRL-2003)
3T3 (cat. CRL-1658)
HEK 293T (cat. CRL-3216)

The following cell line was purchased from Cellbank Australia:
KO52 (CVCL_ CVCL_1321; cat. JCRB0123)

Additional human hematopoietic cell lines were kindly donated by Prof. Wallace Langdon (UWA, Perth, Australia):
MOLM13 (CVCL_2119)
MV411 (CVCL_0064)
PL21 (CVCL_2161)

The following cell line was provided by Dr. Stefan. Froehling:
MM6 (Monomac6, CVCL_1426)

Authentication

Each stocks used of all cell lines were authenticated by STR profiling at early passage before the first culture experiment, performed by the QIMR Berghofer Analytical Core facility.

Mycoplasma contamination

All cell lines tested negative for mycoplasma during regular monthly testing using the biochemical MycoAlertTM Mycoplasma Detection Kit (Lonza) by QIMR Berghofer core facility.

Commonly misidentified lines
(See ICLAC register)

None of the cell lines used have been known as misidentified cell lines according to version 12 of the cross-contamination database maintained by the International Cell Line Authentication Committee (https://iclac.org/databases/cross-contaminations/).

Animals and other research organisms

Policy information about studies involving animals; ARRIVE guidelines recommended for reporting animal research, and Sex and Gender in Research

Laboratory animals	NSG (i.e., NOD.Cg-Prkdcscid Il2rgtm1Wjz /SzJ), NSGS (i.e., NOD.Cg-Prkdcscid Il2rgtm1Wjl Tg[CMV-IL3,CSF2,KITLG]1Eav/MloySzJ), and NRGS mice (i.e., NOD.Cg-Rag1tm1Mom Il2rgtm1Wjl Tg[CMV-IL3,CSF2,KITLG]1Eav/J) were imported from Jackson Laboratories.<br><br>Six to eight week old mice were used for transplantation experiments in this study.
Wild animals	No wild animals were used in this study.
Reporting on sex	Female mice were used as recipients for all experiments in this study.
Field-collected samples	No field-collected samples were used in this study.
Ethics oversight	All mouse experiments were approved by the institutional (QIMR Berghofer) ethics committee protocol A11605M.<br><br>Animals were monitored daily, and always immediately euthanized as soon as a cumulative clinical score of 3 or above was reached, based on weight loss (score 1: > 10 – 20%; score 2: > 20% or > 15% that is maintained for > 72 hours), posture (score 1: hunching noted only at rest; score 2: severe hunching), activity (score 1: mild to moderately decreased; score 2: stationary unless stimulated, hind limb paralysis), and white cell count (score 1: 10-60×106/ml; score 2: > 60×106/ml).

Note that full information on the approval of the study protocol must also be provided in the manuscript.

Flow Cytometry

Plots

Confirm that:

☒ The axis labels state the marker and fluorochrome used (e.g. CD4-FITC).

☒ The axis scales are clearly visible. Include numbers along axes only for bottom left plot of group (a 'group' is an analysis of identical markers).

☒ All plots are contour plots with outliers or pseudocolor plots.

☒ A numerical value for number of cells or percentage (with statistics) is provided.

Methodology

Sample preparation	Flow Cytometry Analysis of AML PDX:<br>For live- dead cell discrimination, Sytox blue (Invitrogen) was used. For monitoring human AML cell engraftment, 25-50 ul of PB was stained after red blood cell lysis (BD Pharmlyse, BD Biosciences) with anti-human CD45 (H130) and anti-mouse Cd45.1 (A20). For AML phenotyping, cell populations were purified from BM (both femurs and tibiae) or SPL after red blood cell lysis and stained with anti-human CD45 (H130), anti-mouse Cd45.1 (A20), anti-human CD34 (581), anti-human CD33 (WM53), anti-human CD38 (HIT2), and anti-human GPR56 (CG4). For the analysis of normal hematopoiesis, PB and SPL cells were stained after red blood cell lysis with anti-human CD45 (H130), anti-mouse Cd45.1 (A20), anti-human CD19 (HIB19), anti-human CD33 (WM53), and anti-human CD3 (SK7). BM cells were isolated from both femurs and tibiae, red cell lysed, and stained with anti-human CD45 (H130), anti-mouse Cd45.1 (A20), anti-human CD34 (581), anti-human CD38 (HIT2), and anti-CD33 (WM53). For apoptosis analysis, cells were stained with Annexin V (BD Biosciences) in Annexin V binding buffer according to the manufacturer's recommendations. For cell-cycle and DNA damage analysis, cells were fixed and permeabilized (Fix & Perm, GAS-004; Invitrogen) according to the manufacturer's instructions and incubated with Ki-67 (B56) and gH2AX (20E3; Cell Signaling Technology). Hoechst 33342 (Invitrogen) was used at 20 mg/ml in PBS containing 2% fetal bovine serum.<br><br>Flow cytometry analysis of AML cell lines:<br>Before staining, 2x10^5 cells were washed with phosphate-buffered saline (PBS) with 2% FCS. For G-quadruplex analysis, cells were then fixed and permeabilized using FIX & PERM Cell Permeabilization Kit (GAS-004; Invitrogen) and incubated with Anti-DNA G-quadruplex structures Antibody, clone BG4 (MABE917; Merck Millipore), and 0.2 mg/mL Hoechst 33342 (Invitrogen).
Instrument	FACS LSR Fortessa (BD Biosciences) for analysis; FACS Aria IIIu (BD Biosciences) for sorting
Software	Flowjo version 10.9.0 (Becton Dickinson & Company (BD)
Cell population abundance	For RNAseq analysis of AML cells from imetelstat or vehicle control-treated PDX, human CD45+ murine Cd45.1- viable singlet populations were between 1-90%, with a confirmed purity of at least 90% after sorting.<br><br>For RNAseq analysis, mutational sequencing and TRF analysis of primary AML patient samples, human CD45+ human CD3- cell populations were between 20-95% with confirmed purity of at least 90% after sorting.
Gating strategy	Peripheral blood donor chimerism flow cytometry:<br>Frequencies of cell populations containing viable singlets (sytox blue-) having stained either positive for CD45 AF647 (patient

donor cells) or Cd45.1 PE (recipient mouse cells) were obtained and used to calculate donor chimerism percentages.

AML phenotyping flow cytometry:
CD45 FITC+ viable singlets were gated on CD33 APC to assess the percentages of myeloid cells, CD34 PE and CD38 APC-Cy7 to assess AML stemness/differentiation, and GPR56 PE-Cy7 as recently described AML stemness marker.

☒ Tick this box to confirm that a figure exemplifying the gating strategy is provided in the Supplementary Information.
