# Supplementary material for: Imetelstat-mediated alterations in fatty acid metabolism to induce ferroptosis as a therapeutic strategy for acute myeloid leukemia
Source: Nat Cancer. 2023 Oct 30;5(1):47–65. doi: 10.1038/s43018-023-00653-5 (PMC10824665; doi:10.1038/s43018-023-00653-5)
Supplement: Supplementary file 3 — Supplementary protocols. [file 43018_2023_653_MOESM3_ESM.pdf]

SUPPLEMENTARY NOTE

Animal housing and feeding conditions

Gamma-irradiated feed and bedding was used. Mouse pellets (cat. 126578) were purchased from Specialty Feeds (https://www.specialtyfeeds.com/) and were provided ad libitum. Optimice mouse IVC cages with standard fine aspen bedding were used. These included a protective filter to prevent the introduction of dust, dander, dirt and potential disease-causing organisms into the cage. Cages were replaced three times per week to maintain clean, dry housing for the animals. Animal rooms were maintained at ventilation (min 16 air changes per hour), temperature (18-24C) and humidity (40-70%). All rooms were maintained on a 12:12 light dark cycle. (7:30am to 7:30pm).

Flow cytometry analysis of cord blood transplants

For the analysis of normal hematopoiesis, PB and SPL cells were stained after red blood cell lysis with anti-human CD45-FITC (H130), anti-mouse Cd45.1-PE (A20), anti-human CD19-PerCP/Cy5.5 (HIB19), anti-human CD33-APC (WM53), and anti-human CD3-APC/Cy7 (SK7). BM cells were isolated from both femurs and tibiae, red cell lysed, and stained with anti-human CD45-FITC (H130), anti-mouse Cd45.1-PerCP/Cy5.5 (A20), anti-human CD34-PE (581), anti-human CD38-APC/Cy7 (HIT2), and anti-CD33-APC (WM53).

Cell lines

The following cell lines were purchased from DSMZ: SKM1 (ACC 547), Monomac6 (ACC 124), MOLM13 (ACC 554), HEL (ACC 11), NB4 (ACC 207), PL21 (ACC 536), and OCIAML3 (ACC 582). K562 (CCL-243), HL60 (CCL-240), U937 (CRL-

1593.2), MV411 (CRL-9591), THP1 (TIB-202), TF1 (CRL-2003), 3T3 (CRL-1658), and HEK 293T (CRL-3216) were purchased from ATCC. KO52 (JCRB0123) was purchased from Cellbank Australia. Additional human hematopoietic cell lines were kindly donated by Prof. Wallace Langdon (MOLM13 (CVCL_2119), MV411 (CVCL_0064), PL21 (CVCL_2161)), and Dr. Stefan. Froehling (Monomac6; MM6; CVCL_1426).

Telomere Length Q-PCR

Samples were purified using the DNeasy Blood and Tissue Kit (QIAGEN). DNA isolation was performed as described previously [61], including degassing of buffers and supplementation with 50 microM of phenyl-tert-butyl nitrone to minimise oxidative damage. Telomere length was assessed using Q-PCR [62-64]. A synthetic oligonucleotide of known length was serially diluted and utilized as a reference standard for telomere length and single copy gene to determine absolute telomere length[61]. Haemoglobin (HBB) and albumin were utilised as single-copy genes to calculate relative telomere/single copy gene (T/S) values and normalize telomere length per cell. Telomere length was validated by terminal restriction fragment (TRF) analysis, and four controls from TRF were included in each run to detect variance, including a long telomere control cell line.

The primers used for amplification were teloF, teloR [61], albu and albd [64] and HBB-f, 5' TGT GCT GGC CCA TCA CTT TG 3' and HBB-r 5' ACC AGC CAC CAC TTT CTG CTA GG 3'. For each sample 5-10 ng of sample DNA were loaded in triplicate. Primers were used at concentrations of 2 microM of forward primer and 18 microM of reverse primer for telomere amplification and 6 microM and 14 microM respectively for single copy genes. DNA was amplified using Quantitect SYBR Green

PCR Kit (QIAGEN) in a total volume of 20uL, and analyzed using an Applied Biosystem ViiA7 thermocycler.

Cloning single guide RNAs into Plko5 vector

Primers (Supplementary Table 1) were phosphorylated by using T4 polynucleotide kinase (EK0031; Thermo Scientific), T4 DNA ligase buffer (46300018; Invitrogen) and were incubated at 37°C for 45 minutes, inactivated at 70°C for 5 minutes and cooled down at 10°C. Plko5 vector was digested by using 10X Tango Yellow Buffer (BY5; Thermo Scientific), BsmBI (ER0451; Thermo Scientific) and 10 mM DTT (Thermo Scientific) and incubated at 37°C for 2 hours. FastAP Thermosensitive Alkaline Phosphatase (EF0654; Thermo Scientific) was added and vector was incubated at 37°C for 20 minutes, followed by 65°C for 20 minutes. Gel electrophoresis was performed and DNA was extracted by QIAQuick DNA Extraction Kit (QIAGEN). Then, 1:500 diluted, phosphorylated primers were ligated into digested Plko5 vector using T4 DNA ligase (New England BioLabs) and T4 DNA ligase buffer (New England BioLabs) by incubating at 22°C for 20 minutes and 65°C for 10 minutes. Vector without insert oligo was set up as a control. Vectors were transformed into E. coli and cultured on ampicillin plates and plasmid DNA was then purified by using Plasmid Maxi Kit (QIAGEN). Insertion of sgRNAs into Plko5 vector was confirmed by sequencing. Single guide RNA target sequences are displayed in Supplementary Table 2. CD33 guide RNAs were used as described previously [67].

Transfection and viral titer determination

Lentiviral particles were produced by transfection of 75% confluent HEK293T cells using Fugene (Promega) with pMD2.G (12559; Addgene) and psPAX2 (12260; Addgene) and Plko5 containing sgRNAs or empty backbone. Viral supernatants were harvested at 42 and 54 hours after transfection and filtered (45 nm pore size). Viral titer was determined by transduction of serial 1:10 dilutions of lentivirus into 3T3 cells using 1:1000 polybrene (Sigma Aldrich).

Generation of Cas9-expressing cell lines

Streptococcus pyogenes Cas9 was stably introduced into the NB4, MV411, KO52 and TF1 cell lines by lentiviral infection. For Cas9/Blasticidin clones, a vector containing a human codon-optimised *Strep. Pyogenes* Cas9 and blasticidin resistance construct expressed from an EFS promoter (pFUGWb) was obtained from Addgene (lentiCas9-Blast, plasmid #52962). 0.5 x 10^6 cells per well were seeded into a 6-well plate in a volume of 1.2 mL. 300 µL of concentrated virus was added to each well with polybrene at a final concentration of 8 µg/mL and centrifuged at 2,500 rpm for 90 minutes at 37°C. Cells were then incubated at 37°C for 48 hours with media replaced at 24 hours.

Lentiviral transduction and sorting

NB4-, MV411-, KO52-, and TF1- expressing Cas9 cells were selected by incubation with blasticidin S HCl (Thermo Scientific; 5 µg/mL for NB4, MV411 and TF1; 1.25 µg/mL for KO52 cells) for two weeks. Cells were then transduced twice with lentivirus using 8 microgram/mL polybrene (Sigma-Aldrich) and 0.01 M Hepes (Thermo Scientific), and were incubated overnight. Then, medium was refreshed and cells were incubated for 48 hours. Final multiplicity of infection (MOI) was 8. In

order to select transduced cells, mCherry+ and Sytox blue- cells were sorted using a FACS AriaIIIu (BD Biosciences), when transduction efficiencies were <96%. CD33, VIM and mCherry expression were analyzed by flow cytometry after passage one. Cells were then stained with 0.6 µg/mL PerCPCy5.5 anti-human CD33 (303414; BioLegend) and 0.05 µM Sytox Blue (S34857; Invitrogen), or anti-human VIM (Vimentin (D21H3) XP® Rabbit mAb (Alexa Fluor® 488 Conjugate) #9854; Cell Signaling) after fixation and permeabilization using the FIX & PERM Cell Permeabilization Kit (GAS-004; Invitrogen). Flow cytometry analysis was performed on FACS LSR Fortessa (BD Biosciences) using DIVA software (BD Biosciences). Analyses were performed using FlowJo software V10.9.0 (Becton Dickinson & Company; BD).

Tracking of indels by decomposition

DNA was extracted using the DNeasy Blood and Tissue Kit (QIAGEN) and amplified using GoTaqGreen Master Mix (Promega, M7123) and primers displayed in Supplementary Table 3. PCR cycling conditions were: 2 minutes 95°C; 30 cycles of 30 seconds at 95°C, 30 seconds at 65°C and 90 seconds at 72°C; and a 5-minute final extension phase at 72°C. The PCR product was analyzed by agarose gel electrophoresis and extracted using the QIAquick gel extraction kit (QIAGEN, 28704) as per manufacturer's protocol. DNA was sequenced with a big dye terminator sequencer and editing efficiency was estimated using TIDE analysis (*https://tide.nki.nl/*). PCR product sizes and sequencing primers are shown in Supplementary Table 4.

Brunello genome-wide CRISPR screen

Brunello library

The Brunello genome-wide gRNA library contains 76,441 gRNAs targeting 19,114 genes and was obtained from Addgene (Cat# 73178). Lentivirus containing the Brunello library was generated.

Infection of cells with Brunello guide RNA library for pooled screens

Optimal infection conditions were determined for each batch of virus in the cell line of interest aiming to achieve 30-50% infection efficiency (corresponding to multiplicity of infection (MOI) of ~0.5-1). Infections of the Brunello library were performed in 12-well plates, seeding 3.0 x 10^6 NB4 + Cas9 cells per well in a total volume of 2 mL. Cells were infected with 25 μL concentrated Brunello library virus with polybrene at a final concentration of 4 μg/mL by centrifuging plates at 2500 rpm for 90 minutes at 37°C. Infection efficiency was determined after 24 hours of incubation at 37°C.

To calculate infection efficiency, cells were replated in puromycin at 2 μg/mL selection dose. Infection efficiency was calculated after 48 hours in puromycin (at the time when non-infected controls were killed by puromycin) by comparing survival of cells with and without puromycin and calculated by the equation:

$$\left(\frac{\#\,Infected\ with\ Puro}{\#\,Infected\ without\ Puro} - \frac{\#\,Uninfected\ with\ Puro}{\#\,Uninfected\ without\ Puro}\right) \times 100$$
$$= Infection\ Rate\ (\%)$$

Cell populations ~30-50% infection efficiency underwent selection for gRNA-containing cells in 2 μg/mL of puromycin for 48 hours before being used for screening.

DNA preparation for guide RNA sequencing

Genomic DNA was extracted using Qiagen DNA kits (appropriate to cell number) according to the manufacturer's protocol. PCR of DNA was performed to attach Illumina sequencing adaptors and barcodes during the amplification of the gRNA region from each cell. Each 100 µL reaction contained a maximum of 10 µg of DNA plus H_2O to a total volume of 50 µL, PCR master mix (40 µL) and 10 µL of a uniquely barcoded P7 primer (5 µM stock) allocated to each replicate. For each reaction, the 40 µL PCR master mix consisted of 0.75 µL of ExTaq DNA Polymerase (Clontech), 10 µL of 10x ExTaq buffer, 8 µL of deoxyribonucleotide triphosphate (dNTP) mix provided with the enzyme, 0.5 µL of P5 stagger primer mix (100 µM stock) and 20.75 µL H_20. The P5 primers attached to a common sequence of the gRNA vector 5' to the 20 nt gRNA insert with a stagger region integrated to increase diversity of the reads allowing the sequencer to recognize each read (Supplementary Table 5). P7 primers bound 3' to the gRNA insert and contain a unique 8 nt barcode to allow assignment of each read to a condition (Supplementary Table 6). PCR cycling conditions were: 1 minute 95°C; 28 cycles of 30 seconds at 94°C, 30 seconds at 52.5°C and 30 seconds at 72°C; and a 10-minute final extension phase at 72°C. The expected product size was 354 bp. P5/P7 primers were synthesized at IDT. Samples were purified with AMPure XP beads (Beckman Coulter, Cat# A63880) using the manufacturer's protocol for right-side selection (to remove residual genomic DNA) and left-side selection (to remove excess primers and dNTPs) and sequenced using the NextSeq 550 Illumina platform.

Guide RNA sequencing analysis

The sequenced gRNA insert from each read was mapped to a reference file of each gRNA in the library. The STARS and RIGER CRISPR screen analysis tools were

applied to the sequencing results with analysis based on log2-transformed numbers of gRNA reads in 'End' samples after drug treatment compared to 'Input' samples.

RNA sequencing: Library preparation

RNA was isolated from a maximum of 0.5 x 10^6 cells using the Qiagen RNeasy Micro kit according to the manufacturer's instructions. Replicates were taken from at least 2 different experiments. RNA samples were quantitated using the Nanodrop Spectrophotometer (Thermo Fischer Scientific) and the Qubit Fluorometer using the Qubit RNA HS Assay Kit (Molecular Probes) according to the manufacturer's recommendations. RNA integrity was confirmed using the RNA 6000 PICO Kit (Agilent Technologies) with analysis using the Agilent 2100 Bioanalyser (Agilent Technologies). Total RNA (100 µg) was used for NGS and prepared according to the NEBNext Ultra II RNA Library Prep Kit for Illumina (New England Biolabs, NEB; Cat# E7770S). Briefly, following mRNA isolation, fragmentation and priming, first and second strand cDNA were synthesised. The double-stranded cDNA was purified, A-tailed and ligated to adaptors. Following purification, the adaptor ligated DNA was enriched by PCR, purified and assessed for quality using the High Sensitivity DNA Kit (Agilent) on the Agilent 2100 Bioanalyser. Libraries were sequenced using a high-output, single-end, 75 cycle (version 2) sequencing kit on the Illumina Nextseq 550 platform.

Lipidomics: Sample preparation

Cells were harvested and washed twice with cold PBS. Cell pellets of one Million cells were used. Eighteen samples were randomized and a blank negative control extraction was included (Supplementary Table 7). All steps were performed on ice.

Cell pellets were re-suspended in 10 µL of pre-chilled milliQ water before adding 200 µL of ice-cold butanol/methanol (1:1) containing 10 mM ammonium formate and 50 µg/mL antioxidant 2,6-di-tert-butyl-4-methylphenol (BHT) to extract lipids. An aliquot of 10 µL of a 1/10-diluted SPLASH Lipidomix (Avanti, pn 330707) was spiked into each sample to assess sample preparation and retention time variation. Samples were incubated in a Thermomixer for 1 hr at 4°C and 850 rpm, followed by centrifugation for 15 min at 16,000 rcf (4°C). Supernatants were removed and dried down using a vacuum concentrator. For LC/MS analysis, dried samples were re-suspended in 50 µL of ice-cold methanol/toluene (9:1, v/v) containing 100 ng/mL 12-[[(cyclohexylamino)carbonyl]amino]-dodecanoic acid (CUDA). The CUDA standard was used to flag autosampler inaccuracies. A sample pool was prepared by combining 5 µL of each sample.

Lipidomics: Targeted LC/MS analysis

Lipidomics was performed according to a method by Huynh and co-workers [76] with slight modifications. The LC/MS platform consisted of a 1290 Infinity II UHPLC coupled to a 6470 QQQ mass spectrometer via AJS ESI source (Agilent, Santa Clara, USA). The mass spectrometer was operated in positive ionization mode acquiring data in scheduled multiple reaction monitoring (MRM). Quadrupoles 1 and 2 were set to unit resolution. The MRM transition list (Supplementary Table 8) contained 20 lipid classes and 593 lipid species (excluding internal standards CUDA and SPLASH Lipidomix) with the lipid naming convention used here also adopted from Huynh and co-workers [76].

Separation was performed on a Zorbax Eclipse Plus C18 RRHD (1.8 µm, 95 Å, 2.1 x 100 mm) analytical column connected to a 2.1 x 5 mm guard column of the same

resin. The autosampler and column temperature were set to 4°C and 60°C, respectively. Solvent system and gradient were used as described previously [76] and sample injection was 3 μL.

Source conditions were as follows: Gas temperature 175°C, gas flow 11 L/min, sheath gas temperature and flow at 250°C and 10 L/min, respectively, nebulizer 20 psi, fragmentor 135, capillary voltage at +4750 V, nozzle voltage was zero.

Prior to running samples, the originally published MRM transition list (20 lipid classes and 636 lipid species) was assessed for the presence/absence of lipid species in the current sample matrix using the sample pool. This was performed by splitting the transition list, running 3 scheduled MRM methods with a larger retention time window of 5 min. Following the analysis, the final scheduled MRM assay contained 609 transitions from 606 lipid species (including 13 ISTD lipid species and 16 transitions) with retention time windows of 1.5-4 min (depending on the lipid class and species, and a cycle time of 1 s resulting in a maximum of 177 concurrent MRMs and a minimum dwell time of 3.2 ms. All samples were run in a randomized order bracketed by a sample pool as a quality control.

Lipidomics: Data analysis

Skyline-daily software [77] was used for lipid species assignment (precursor/product ion pairs and retention time) and chromatographic peak integration based on Huynh and co-workers [76] (Supplementary Table 9). An indexed retention time (iRT) calculator, which was generated using internal standards as well as lipid species assigned from the sample pool run, was employed for a retention time predictor to increase confidence of lipid assignment in subsequent samples. Peak picking was manually inspected using Skyline's retention times – replicate comparison pane and adjusted

accordingly by comparing retention time and chromatographic peak profile to the sample pool QC run. Furthermore, all peaks were manually checked for correct integration.

The downstream data processing and visualization was carried out with R package lipidr (version 2.14.1) [78] (Supplementary Table 10). Raw data quality was assessed by plotting total lipid intensities of each sample as well as intensity and retention time distributions of internal standards across samples. Log2 transformation and probabilistic quotient normalization (PQN) was performed prior to statistical analysis. Sample variation was investigated by principal component analysis (PCA). A lipid set enrichment analysis was performed by ranking fold changes, calculating enrichment scores and estimating the significance of enrichment using a permutation algorithm [78]. The enrichment results were plotted as boxplot or trend line.
