# Supplementary material for: Imetelstat-mediated alterations in fatty acid metabolism to induce ferroptosis as a therapeutic strategy for acute myeloid leukemia
Source: Nat Cancer. 2023 Oct 30;5(1):47–65. doi: 10.1038/s43018-023-00653-5 (PMC10824665; doi:10.1038/s43018-023-00653-5)

Western blot analysis of ACSL4 in loss-of-function edited NB4 cells

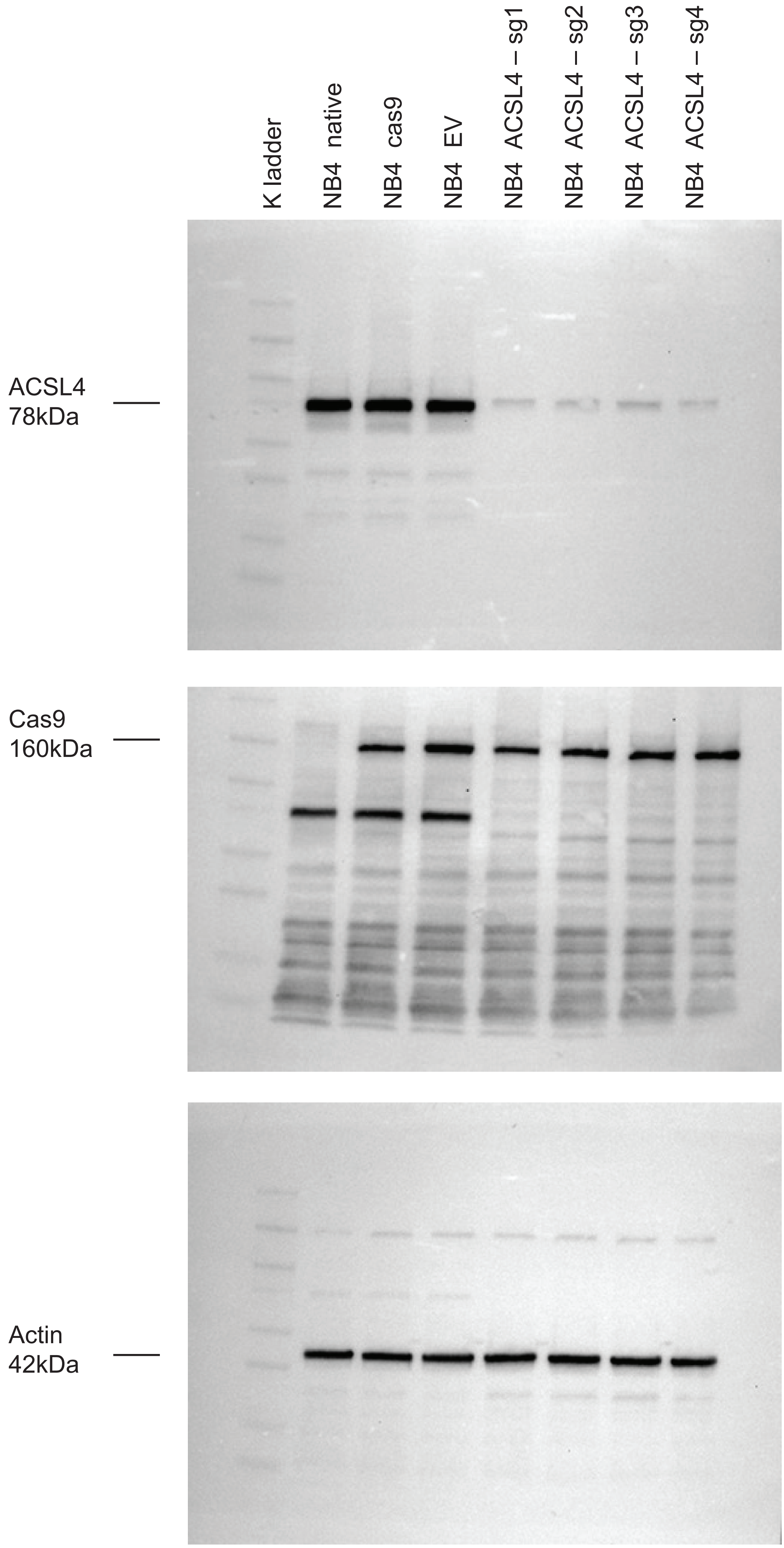

Western blot analysis of ACSL4 in loss-of-function edited TF1 cells

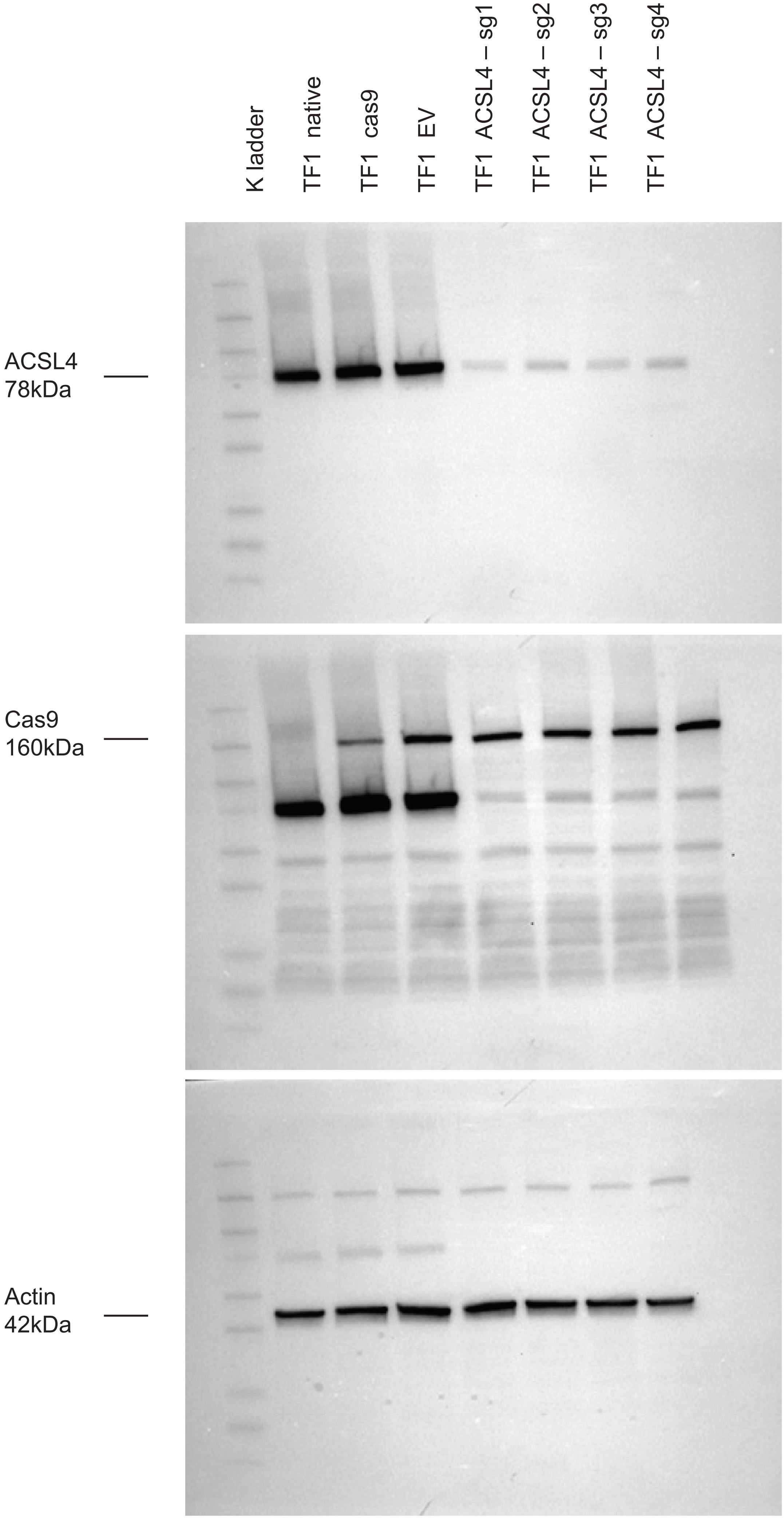

Western blot analysis of ACSL4 in loss-of-function edited KO52 cells

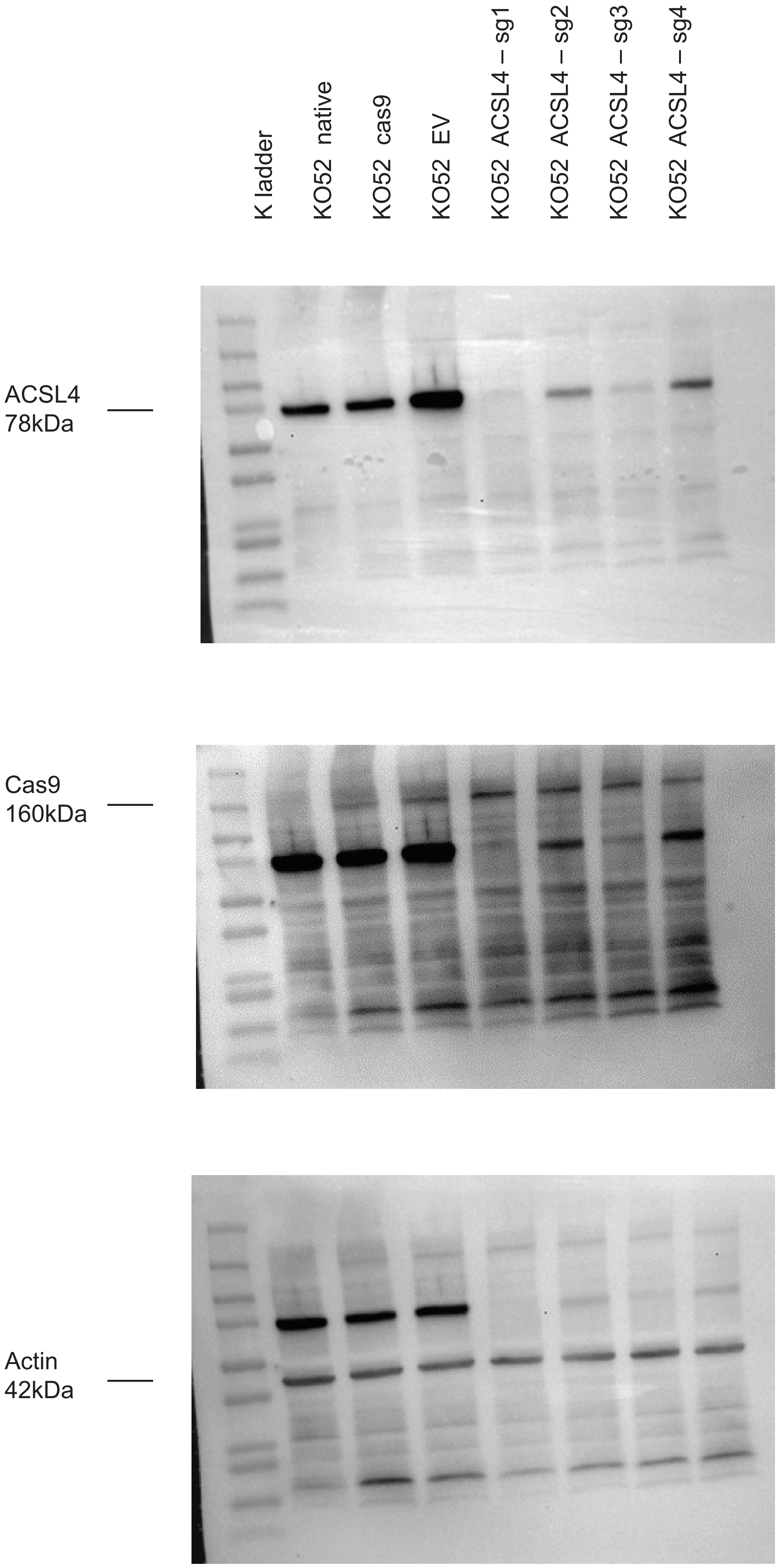

Western blot analysis of ACSL4 in loss-of-function edited MV411 cells

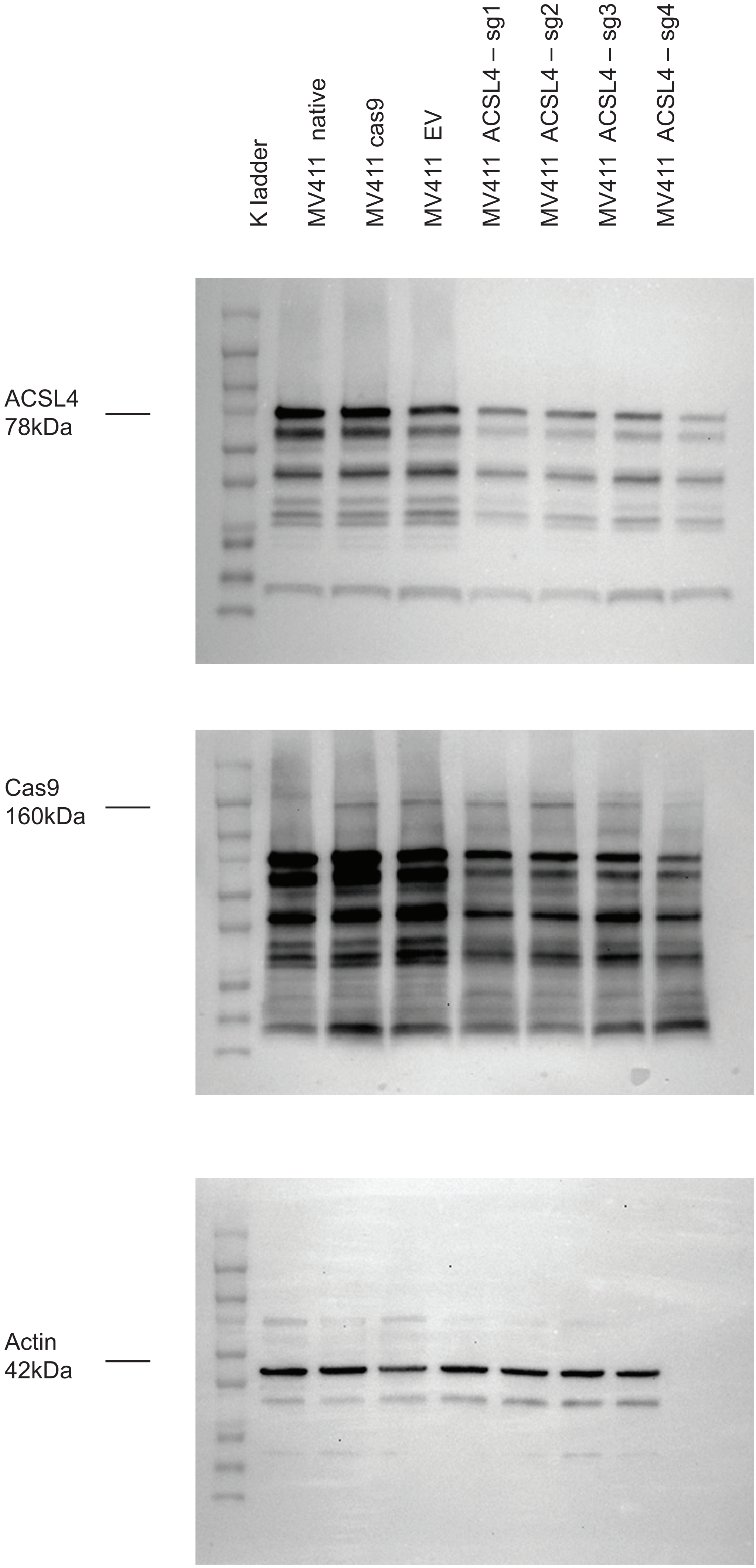

Supplement: Supplementary file 17 — Uncropped blots. [file 43018_2023_653_MOESM17_ESM.pdf]
